# Perceptions and challenges of online teaching and learning amidst the COVID-19 pandemic in India: a cross-sectional study with dental students and teachers

**DOI:** 10.1186/s12909-024-05340-2

**Published:** 2024-06-06

**Authors:** Lakshmi Nidhi Rao, Aditya Shetty, Varun Pai, Srikant Natarajan, Manjeshwar Shrinath Baliga, Dian Agustin Wahjuningrum, Heeresh Shetty, Irmaleny Irmaleny, Ajinkya M. Pawar

**Affiliations:** 1 Department of Conservative Dentistry and Endodontics, AB Shetty Memorial Institute of Dental Sciences, NITTE Deemed to be University, Mangalore, Karnataka India; 2 Forensic Medicine Unit, Faculty of Medicine, AIMST University, Jalan Semeling, Bedong, Kedah Malaysia; 3 Department of Oral Pathology and Microbiology, Manipal College of Dental Sciences, Mangalore, Karnataka India; 4 Research Unit, Mangarole Institute of Oncology, Pumpwell, Mangalore, Karnataka 575002 India; 5 Department of Conservative Dentistry, Faculty of Dental Medicine, Universitas Airlangga, Surabaya, Indonesia; 6 Department of Conservative Dentistry and Endodontics, Nair Hospital Dental College, Mumbai, Maharashtra 400008 India; 7 Department of Conservative Dentistry, Universitas Padjadjaran, Bandung, Indonesia

**Keywords:** Coronavirus pandemic, COVID-19, Face-to-face-instruction, Online education, Education and training, Worldwide web technology

## Abstract

**Background:**

Online education has emerged as a crucial tool for imparting knowledge and skills to students in the twenty-first century, especially in developing nations like India, which previously relied heavily on traditional teaching methods.

**Methods:**

This study delved into the perceptions and challenges experienced by students and teachers in the context of online education during the COVID-19 pandemic. Data were collected from a sample of 491 dental students and 132 teachers utilizing a cross-sectional research design and an online-validated survey questionnaire.

**Results:**

The study’s findings revealed significant insights. Internet accessibility emerged as a major impediment for students, with online instruction proving more effective for theoretical subjects compared to practical ones. Although most teachers expressed comfort with online teaching, they highlighted the absence of classroom interaction as a significant challenge.

**Conclusion:**

This study comprehensively examines the perspectives of both students and teachers regarding online education during the pandemic. The results carry substantial implications for the academic community, underscoring the need to address internet access issues and explore ways to enhance engagement and interaction in online learning environments.

**Supplementary Information:**

The online version contains supplementary material available at 10.1186/s12909-024-05340-2.

## Introduction

The COVID-19 pandemic has undeniably reshaped the global educational landscape, forcing a rapid shift towards online learning methodologies. While some disciplines have transitioned relatively smoothly, dental education presents unique challenges. Unlike fields with a primarily theoretical foundation, dental education hinges on the development of practical skills and direct patient interaction [1, 2]. This inherent need for hands-on clinical experience necessitates a critical examination of online learning’s suitability for dental education [1].

Research across diverse international contexts underscores the limitations of online learning alone in fostering essential technical skills in dentistry [3, 4]. Recognizing this reality, the Indian dental education model prioritizes hands-on learning as a core curricular element. However, the pre-clinical phases often incorporate simulations using mannequins, hinting at the potential for blended learning approaches. In this scenario, online platforms could be strategically utilized to deliver theoretical knowledge, thereby freeing up valuable classroom time for instructors to conduct in-person skill development sessions with students [5, 6].

Despite advancements in technology, digitalization efforts in the Indian dental sector have primarily focused on practical training tools like computer-aided design/computer-aided manufacturing (CAD/CAM) and 3D printing technologies [7, 8]. Traditional face-to-face lectures remained the dominant method for knowledge delivery, with online learning remaining largely unexplored within the Indian dental education curriculum before the pandemic [9].

The COVID-19 pandemic has disrupted this status quo, propelling online learning to the forefront of dental education [10]. This unprecedented situation necessitates a comprehensive assessment of its impact on the perceptions and experiences of both dental students and educators across India. This leads us to our central research question: “To what extent has the COVID-19 pandemic impacted the perceptions and challenges of online learning among dental students and teachers in India?”

By delving into this question, we aim to shed light on the strengths, weaknesses, and areas for improvement in online learning within the context of Indian dental education. These findings will inform future curricular development, allowing for a well-considered and strategic integration of online and traditional approaches. Ultimately, this research seeks to enhance the overall educational experience for dental students. By ensuring a balanced curriculum that leverages the strengths of both online and offline learning, we can equip future dentists with the essential knowledge and practical skills necessary to thrive in a rapidly evolving healthcare landscape.

## Methods

### Study design

The study utilized a cross-sectional research design to collect data from 500 dental students and 150 teachers in India. An online-validated survey questionnaire was employed to gather quantitative data. The study population consisted of undergraduate and postgraduate dental students and teachers from diverse dental colleges across India. Participants were selected through purposive sampling based on their willingness and availability during the study period. Ethical principles were strictly followed, including obtaining informed consent, ensuring confidentiality of participant data, and safeguarding participant privacy. This sampling method was chosen due to practical reasons, as randomly sampling would have been resource intensive. Leveraging existing networks and professional contacts facilitated access to a varied participant pool, ensuring engagement and data quality. To enhance representativeness, participants from various dental colleges, urban and rural locations, academic levels, and age groups were included in the sample.

### Questionnaire

A self-administered, English-language questionnaire developed using Google Forms was utilized to evaluate perceptions and challenges of online dental education during the COVID-19 pandemic in India [11]. The questionnaire was structured around three main domains: satisfaction with online teaching, encountered problems, and comparisons between online and traditional classroom learning experiences.

In order to ensure the validity and reliability of this questionnaire within the unique context of Indian dental education, a thorough validation process was undertaken. Face validity was established through evaluation by a qualified researcher and questionnaire design specialist. Their assessment focused on the clarity, comprehensiveness, and relevance of the questions, resulting in revisions to improve clarity and minimize ambiguity in terminology, phrasing, and structure.

Content validity was ensured through the input of two subject-matter experts (SMEs) with significant experience in Indian dental education. These SMEs, who were independent of the study, assessed the questionnaire against the defined research objectives. Their feedback ensured that the questionnaire comprehensively covered the intended constructs, leading to further refinements.

Pilot testing was then conducted with a representative sample of 20 dental students and 10 teachers. This phase aimed to identify and address any remaining issues with the questionnaire’s understandability, flow, and length. Based on the feedback received from the pilot test participants, minor adjustments were made to optimize the user experience.

### Data analysis

Following data collection, survey responses were entered into a Microsoft Excel spreadsheet and then imported into the Statistical Package for Social Sciences (SPSS) version 25 for analysis. Descriptive statistics were employed to summarize participant characteristics such as age, course of study (undergraduate, postgraduate), place of study (town, village), and self-reported familiarity with e-learning skills. These characteristics were presented as frequencies (N) and percentages (%) to provide an overview of the sample composition.

Chi-square tests were conducted to assess potential associations between categorical variables. However, the use of the Chi-square test is contingent upon meeting the assumption of expected cell frequencies being greater than 5. In instances where expected cell frequencies fell below 5, Fisher’s exact test was employed as a more appropriate alternative. Statistical significance was established at a *p*-value of 0.05 or less.

## Results

### Participant characteristics and survey completion

A total of 500 students initiated the online survey, with a completion rate of 81.8% (*n* = 409). Similarly, among the 150 teachers who began the survey, 132 completed it (completion rate: 88%). To ensure a sufficient sample size for analysis, the survey period was extended beyond its original timeframe, potentially introducing a selection bias. This decision aligns with the purposive sampling methodology employed in this study.

### Student perceptions

#### Satisfaction with online learning

A significant portion (44.7%, *n* = 183) of students aged 18–21 reported satisfaction with online instruction. Interestingly, age did not significantly influence satisfaction levels. Undergraduates expressed higher satisfaction compared to other course levels (*p* = 0.001). Location also played a role, with students from both urban (41.8%, *n* = 79) and rural areas (45.6%, *n* = 73) reporting similar contentment levels (*p*-value = 0.034). Notably, students with advanced e-learning skills reported significantly higher satisfaction (*p*-value = 0.001).

#### Evaluation of specific aspects

Students across various age groups, locations, and course levels expressed satisfaction with the topics covered (*p* = 0.032 for undergraduate students, *p* = 0.002 for those knowledgeable about e-learning) and the instructors’ efforts (particularly those aged 18–21, *p* = 0.001, undergraduates, *p* = 0.010, and students with e-learning skills, *p* = 0.001). However, no significant difference was observed in self-reported understanding of the subject matter based on demographics or e-learning skills. Overall, students aged 18–21 (42.7%, *p* = 0.001) and those with e-learning knowledge (*p* = 0.006) exhibited greater appreciation for the quality of teaching.

#### Engagement and flexibility

Among participants familiar with e-learning (specific number not provided), a significant proportion (42.9%, *p*-value of 0.019) felt they could effectively engage with instructors during and after online sessions, regardless of age, location, or course level. Additionally, a notable number of undergraduate students with e-learning skills (*p*-values of 0.039 and 0.001, respectively) appreciated the flexibility of attending online classes at their convenience. Furthermore, 40.2% of participants with e-learning skills (*p*-value = 0.054) found online learning beneficial, particularly for theoretical subjects lacking practical components. Notably, a majority of participants across demographics agreed that online teaching could be valuable for future mass education initiatives (data presented in Table 1).

**Table 1:** Satisfaction of students with respect to online teaching regarding teaching through online during the Covid 19 pandemic in India

	Categories (*n* = 409)	Age		Chi square/Fishers exact *P* value	Course of study		Chi square/Fishers exact *P* value	Place			Chi square/Fishers exact *P* value	E learning skills		Chi square/Fishers exact *P* value
		18–21 years 300 (N %)	> 21 years 109 (N %)		UG (N %)	PG (N %)		Village (N %)	Town (N %)	City (N %)		yes (N %)	no (N %)	
**I was satisfied with online teaching by my teacher**	Strongly disagree	20 (6.7)	7 (6.4)	4.824 0.306	20 (6.2)	7 (8.1)	18.13 **0.001**	6 (3.8)	6 (10)	15 (7.9)	16.624 **0.034**	13 (4)	14 (16.9)	21.298 **< 0.001**
	Disagree	37 (12.3)	13 (11.9)		30 (9.3)	20 (23.3)		27 (16.9)	6 (10)	17 (9)		38 (11.7)	12 (14.5)	
	Neutral	61 (20.3)	33 (30.3)		70 (21.7)	24 (27.9)		34 (21.2)	20 (33.3)	40 (21.2)		75 (23)	19 (22.9)	
	Agree	131 (43.7)	42 (38.5)		148 (45.8)	25 (29.1)		73 (45.6)	21 (35)	79 (41.8)		142 (43.6)	31 (37.3)	
	Strongly agree	51 (17)	14 (12.8)		55 (17)	10 (11.6)		20 (12.5)	7 (11.7)	38 (20.1)		58 (17.8)	7 (8.4)	
**I was satisfied with discussion on the topic**	Strongly disagree	11 (3.7)	5 (4.6)	6.209 0.184	12 (3.7)	4 (4.7)	10.593 **0.032**	2 (1.2)	4 (6.7)	10 (5.3)	29.217 **< 0.001**	8 (2.5)	8 (9.6)	16.999 **0.002**
	Disagree	51 (17)	11 (10.1)		43 (13.3)	19 (22.1)		27 (16.9)	10 (16.7)	25 (13.2)		47 (14.4)	15 (18.1)	
	Neutral	55 (18.3)	29 (26.6)		60 (18.6)	24 (27.9)		41 (25.6)	19 (31.7)	24 (12.7)		61 (18.7)	23 (27.7)	
	Agree	145 (48.3)	54 (49.5)		168 (52)	31 (36)		80 (50)	22 (36.7)	97 (51.3)		167 (51.2)	32 (38.6)	
	Strongly agree	38 (12.7)	10 (9.2)		40 (12.4)	8 (9.3)		10 (6.2)	5 (8.3)	33 (17.5)		43 (13.2)	5 (6)	
**I was satisfied with efforts the teacher put in**	Strongly disagree	6 (2)	4 (3.7)	14.524 **0.004**	8 (2.5)	2 (2.3)	12.606 **0.010**	1 (0.6)	1 (1.7)	8 (4.2)	24.206 **0.001**	6 (1.8)	4 (4.8)	25.307 **< 0.001**
	Disagree	13 (4.3)	0 (0)		12 (3.7)	1 (1.2)		2 (1.2)	5 (8.3)	6 (3.2)		6 (1.8)	7 (8.4)	
	Neutral	25 (8.3)	9 (8.3)		22 (6.8)	12 (14)		13 (8.1)	10 (16.7)	11 (5.8)		25 (7.7)	9 (10.8)	
	Agree	136 (45.3)	67 (61.5)		152 (47.1)	51 (59.3)		86 (53.8)	32 (53.3)	85 (45)		154 (47.2)	49 (59)	
	Strongly agree	120 (40)	29 (26.6)		129 (39.9)	20 (23.3)		58 (36.2)	12 (20)	79 (41.8)		135 (41.4)	14 (16.9)	
**Online teaching helped me understand the subject better**	Strongly disagree	47 (15.7)	12 (11)	3.363 0.499	48 (14.9)	11 (12.8)	5.495 0.240	18 (11.2)	10 (16.7)	31 (16.4)	11.478 0.176	40 (12.3)	19 (22.9)	8.94 0.071
	Disagree	63 (21)	21 (19.3)		61 (18.9)	23 (26.7)		35 (21.9)	19 (31.7)	30 (15.9)		68 (20.9)	16 (19.3)	
	Neutral	91 (30.3)	42 (38.5)		105 (32.5)	28 (32.6)		54 (33.8)	15 (25)	64 (33.9)		105 (32.2)	28 (33.7)	
	Agree	77 (25.7)	28 (25.7)		83 (25.7)	22 (25.6)		45 (28.1)	12 (20)	48 (25.4)		87 (26.7)	18 (21.7)	
	Strongly agree	22 (7.3)	6 (5.5)		26 (8)	2 (2.3)		8 (5)	4 (6.7)	16 (8.5)		26 (8)	2 (2.4)	
**Overall, I was satisfied with the quality of learning I had from online teaching**	Strongly disagree	38 (12.7)	6 (5.5)	18.885 **0.001**	38 (11.8)	6 (7)	8.238 0.083	14 (8.8)	9 (15)	21 (11.1)	13.295 0.102	31 (9.5)	13 (15.7)	14.412 **0.006**
	Disagree	57 (19)	28 (25.7)		61 (18.9)	24 (27.9)		40 (25)	15 (25)	30 (15.9)		61 (18.7)	24 (28.9)	
	Neutral	52 (17.3)	35 (32.1)		64 (19.8)	23 (26.7)		37 (23.1)	14 (23.3)	36 (19)		65 (19.9)	22 (26.5)	
	Agree	128 (42.7)	37 (33.9)		135 (41.8)	30 (34.9)		62 (38.8)	20 (33.3)	83 (43.9)		144 (44.2)	21 (25.3)	
	Strongly agree	25 (8.3)	3 (2.8)		25 (7.7)	3 (3.5)		7 (4.4)	2 (3.3)	19 (10.1)		25 (7.7)	3 (3.6)	
**It was possible to interact with the teacher privately in multiple ways during or after online teaching**	Strongly disagree	20 (6.7)	4 (3.7)	4.778 0.311	19 (5.9)	5 (5.8)	3.02 0.555	8 (5)	3 (5)	13 (6.9)	4.029 0.854	16 (4.9)	8 (9.6)	11.817 **0.019**
	Disagree	56 (18.7)	14 (12.8)		53 (16.4)	17 (19.8)		26 (16.2)	14 (23.3)	30 (15.9)		49 (15)	21 (25.3)	
	Neutral	82 (27.3)	34 (31.2)		87 (26.9)	29 (33.7)		43 (26.9)	15 (25)	58 (30.7)		90 (27.6)	26 (31.3)	
	Agree	113 (37.7)	49 (45)		133 (41.2)	29 (33.7)		68 (42.5)	24 (40)	70 (37)		140 (42.9)	22 (26.5)	
	Strongly agree	29 (9.7)	8 (7.3)		31 (9.6)	6 (7)		15 (9.4)	4 (6.7)	18 (9.5)		31 (9.5)	6 (7.2)	
**It was possible for me to access online classes anytime and from anywhere**	Strongly disagree	32 (10.7)	14 (12.8)	15.127 **0.004**	32 (9.9)	14 (16.3)	10.116 **0.039**	20 (12.5)	4 (6.7)	22 (11.6)	17.422 **0.026**	28 (8.6)	18 (21.7)	26.006 **< 0.001**
	Disagree	48 (16)	32 (29.4)		56 (17.3)	24 (27.9)		35 (21.9)	13 (21.7)	32 (16.9)		55 (16.9)	25 (30.1)	
	Neutral	55 (18.3)	11 (10.1)		53 (16.4)	13 (15.1)		32 (20)	12 (20)	22 (11.6)		52 (16)	14 (16.9)	
	Agree	107 (35.7)	41 (37.6)		122 (37.8)	26 (30.2)		57 (35.6)	23 (38.3)	68 (36)		128 (39.3)	20 (24.1)	
	Strongly agree	58 (19.3)	11 (10.1)		60 (18.6)	9 (10.5)		16 (10)	8 (13.3)	45 (23.8)		63 (19.3)	6 (7.2)	
**Online is useful for theory subjects**	Strongly disagree	28 (9.3)	7 (6.4)	2.017 0.733	26 (8)	9 (10.5)	2.592 0.628	13 (8.1)	4 (6.7)	18 (9.5)	13.947 0.083	24 (7.4)	11 (13.3)	9.32 0.054
	Disagree	52 (17.3)	23 (21.1)		56 (17.3)	19 (22.1)		40 (25)	5 (8.3)	30 (15.9)		53 (16.3)	22 (26.5)	
	Neutral	59 (19.7)	18 (16.5)		61 (18.9)	16 (18.6)		28 (17.5)	17 (28.3)	32 (16.9)		66 (20.2)	11 (13.3)	
	Agree	117 (39)	43 (39.4)		132 (40.9)	28 (32.6)		56 (35)	27 (45)	77 (40.7)		131 (40.2)	29 (34.9)	
	Strongly agree	44 (14.7)	18 (16.5)		48 (14.9)	14 (16.3)		23 (14.4)	7 (11.7)	32 (16.9)		52 (16)	10 (12)	
**Online teaching will be useful in mass teaching (by one teacher)**	Strongly disagree	40 (13.3)	11 (10.1)	3.734 0.443	39 (12.1)	12 (14)	4.75 0.314	14 (8.8)	8 (13.3)	29 (15.3)	14.257 0.075	34 (10.4)	17 (20.5)	6.865 0.143
	Disagree	56 (18.7)	27 (24.8)		59 (18.3)	24 (27.9)		44 (27.5)	8 (13.3)	31 (16.4)		65 (19.9)	18 (21.7)	
	Neutral	65 (21.7)	24 (22)		72 (22.3)	17 (19.8)		32 (20)	16 (26.7)	41 (21.7)		73 (22.4)	16 (19.3)	
	Agree	100 (33.3)	38 (34.9)		114 (35.3)	24 (27.9)		53 (33.1)	24 (40)	61 (32.3)		114 (35)	24 (28.9)	
	Strongly agree	39 (13)	9 (8.3)		39 (12.1)	9 (10.5)		17 (10.6)	4 (6.7)	27 (14.3)		40 (12.3)	8 (9.6)	

#### Challenges with online learning

Despite some advantages, participants with e-learning skills (48.2%) also reported internet connectivity and speed issues. Slow internet hindered video streaming for students across all age groups (*p* = 0.005). Only 20.6% of participants with e-learning skills disagreed with this finding.

#### Interaction and collaboration

Except for those residing in rural and semi-urban areas (*p* = 0.022), participants did not report significant concerns about general interaction problems. However, challenges emerged regarding sound quality and group study. Poor internet connections caused sound issues for students above 21 years old (55%, *p* = 0.02) and those without e-learning skills (27.7%, *p* = 0.03). Similarly, joint or group study proved difficult for participants over 21 (55%) and those residing in rural areas (48.8%, *p* = 0.025).

#### Subject suitability

A significant portion (41%) of participants unfamiliar with e-learning skills expressed concerns about the effectiveness of online learning for subjects like mathematics, accounting, and laboratory-based courses (*p* = 0.077). This suggests that students perceive these subjects as requiring a more hands-on or interactive approach that may be challenging to replicate in an online environment.

#### Learning environment

Across all demographics, a consistent trend emerged: most participants reported feelings of isolation and a lack of belonging when learning online (data presented in Table 2). This indicates that online learning environments may not adequately foster the sense of community and social interaction typically found in traditional classrooms. Students generally favoured classroom settings for the increased engagement and interaction with teachers and classmates, qualities perceived as lacking in online environments. This preference was further supported by students with limited e-learning skills (33.7%), who agreed that classroom learning was superior and considered online teaching/learning to be less beneficial (*p* = 0.045).

**Table 2:** Problems faced by students regarding teaching through online during the Covid 19 pandemic in India

	Categories (*n* = 409)	Age		Chi square/Fishers exact *P* value	Course of study		Chi square/Fishers exact *P* value	Place			Chi square/Fishers exact *P* value	E learning skills		Chi square/Fishers exact *P* value
		18–21 years 300 (N %)	> 21 years 109 (N %)		UG (%)	PG (N %)		Village (N %)	Town (N %)	City (N %)		yes (N %)	no (N %)	
**I had problems with the internet connection and speed**	Strongly disagree	14 (4.7)	0 (0)	8.778 0.064	13 (4)	1 (1.2)	3.968 0.409	5 (3.1)	0 (0)	9 (4.8)	13.384 0.099	10 (3.1)	4 (4.8)	13.26 **0.01**
	Disagree	46 (15.3)	16 (14.7)		48 (14.9)	14 (16.3)		20 (12.5)	8 (13.3)	34 (18)		52 (16)	10 (12)	
	Neutral	43 (14.3)	10 (9.2)		45 (13.9)	8 (9.3)		23 (14.4)	13 (21.7)	17 (9)		46 (14.1)	7 (8.4)	
	Agree	133 (44.3)	56 (51.4)		143 (44.3)	46 (53.5)		70 (43.8)	27 (45)	92 (48.7)		157 (48.2)	32 (38.6)	
	Strongly agree	64 (21.3)	27 (24.8)		74 (22.9)	17 (19.8)		42 (26.2)	12 (20)	37 (19.6)		61 (18.7)	30 (36.1)	
**I used to have problems with the video streaming due to slow internet**	Strongly disagree	13 (4.3)	0 (0)	14.37 **0.005**	13 (4)	0 (0)	6.455 0.160	3 (1.9)	1 (1.7)	9 (4.8)	4.103 0.854	10 (3.1)	3 (3.6)	8.766 0.067
	Disagree	50 (16.7)	25 (22.9)		58 (18)	17 (19.8)		28 (17.5)	9 (15)	38 (20.1)		67 (20.6)	8 (9.6)	
	Neutral	38 (12.7)	4 (3.7)		37 (11.5)	5 (5.8)		18 (11.2)	7 (11.7)	17 (9)		34 (10.4)	8 (9.6)	
	Agree	143 (47.7)	59 (54.1)		155 (48)	47 (54.7)		79 (49.4)	32 (53.3)	91 (48.1)		161 (49.4)	41 (49.4)	
	Strongly agree	56 (18.7)	21 (19.3)		60 (18.6)	17 (19.8)		32 (20)	11 (18.3)	34 (18)		54 (16.6)	23 (27.7)	
**I had problems in interacting with the teacher**	Strongly disagree	21 (7)	3 (2.8)	4.07 **0.397**	20 (6.2)	4 (4.7)	4.729 0.316	5 (3.1)	1 (1.7)	18 (9.5)	17.866 **0.022**	18 (5.5)	6 (7.2)	6.121 0.19
	Disagree	97 (32.3)	31 (28.4)		104 (32.2)	24 (27.9)		42 (26.2)	20 (33.3)	66 (34.9)		111 (34)	17 (20.5)	
	Neutral	66 (22)	25 (22.9)		76 (23.5)	15 (17.4)		42 (26.2)	14 (23.3)	35 (18.5)		71 (21.8)	20 (24.1)	
	Agree	92 (30.7)	41 (37.6)		97 (30)	36 (41.9)		61 (38.1)	21 (35)	51 (27)		102 (31.3)	31 (37.3)	
	Strongly agree	24 (8)	9 (8.3)		26 (8)	7 (8.1)		10 (6.2)	4 (6.7)	19 (10.1)		24 (7.4)	9 (10.8)	
**I had confusion with logging in and operating the online process**	Strongly disagree	57 (19)	5 (4.6)	15.522 **0.004**	59 (18.3)	3 (3.5)	12.03 **0.017**	15 (9.4)	4 (6.7)	43 (22.8)	28.002 **< 0.001**	54 (16.6)	8 (9.6)	18.738 **0.001**
	Disagree	139 (46.3)	54 (49.5)		149 (46.1)	44 (51.2)		73 (45.6)	28 (46.7)	92 (48.7)		162 (49.7)	31 (37.3)	
	Neutral	43 (14.3)	21 (19.3)		47 (14.6)	17 (19.8)		27 (16.9)	15 (25)	22 (11.6)		51 (15.6)	13 (15.7)	
	Agree	51 (17)	21 (19.3)		54 (16.7)	18 (20.9)		38 (23.8)	12 (20)	22 (11.6)		50 (15.3)	22 (26.5)	
	Strongly agree	10 (3.3)	8 (7.3)		14 (4.3)	4 (4.7)		7 (4.4)	1 (1.7)	10 (5.3)		9 (2.8)	9 (10.8)	
**I had problems with sound due to slow internet**	Strongly disagree	22 (7.3)	1 (0.9)	16.812 **0.002**	21 (6.5)	2 (2.3)	7.013 0.135	6 (3.8)	3 (5)	14 (7.4)	12.164 0.144	16 (4.9)	7 (8.4)	10.749 **0.03**
	Disagree	62 (20.7)	21 (19.3)		65 (20.1)	18 (20.9)		29 (18.1)	11 (18.3)	43 (22.8)		69 (21.2)	14 (16.9)	
	Neutral	51 (17)	8 (7.3)		51 (15.8)	8 (9.3)		18 (11.2)	14 (23.3)	27 (14.3)		51 (15.6)	8 (9.6)	
	Agree	113 (37.7)	60 (55)		128 (39.6)	45 (52.3)		81 (50.6)	22 (36.7)	70 (37)		142 (43.6)	31 (37.3)	
	Strongly agree	52 (17.3)	19 (17.4)		58 (18)	13 (15.1)		26 (16.2)	10 (16.7)	35 (18.5)		48 (14.7)	23 (27.7)	
**Group study is difficult in online learning**	Strongly disagree	14 (4.7)	1 (0.9)	9.760 **0.042**	12 (3.7)	3 (3.5)	4.498 0.340	2 (1.2)	1 (1.7)	12 (6.3)	17.494 **0.025**	11 (3.4)	4 (4.8)	6.211 0.184
	Disagree	61 (20.3)	14 (12.8)		62 (19.2)	13 (15.1)		27 (16.9)	15 (25)	33 (17.5)		64 (19.6)	11 (13.3)	
	Neutral	52 (17.3)	14 (12.8)		56 (17.3)	10 (11.6)		17 (10.6)	12 (20)	37 (19.6)		56 (17.2)	10 (12)	
	Agree	119 (39.7)	53 (48.6)		135 (41.8)	37 (43)		78 (48.8)	21 (35)	73 (38.6)		137 (42)	35 (42.2)	
	Strongly agree	54 (18)	27 (24.8)		58 (18)	23 (26.7)		36 (22.5)	11 (18.3)	34 (18)		58 (17.8)	23 (27.7)	
**Online learning is not suitable for subjects like mathematics, accounts, laboratory based subjects**	Strongly disagree	14 (4.7)	1 (0.9)	6.836 0.142	13 (4)	2 (2.3)	3.923 0.416	5 (3.1)	1 (1.7)	9 (4.8)	5.506 0.705	11 (3.4)	4 (4.8)	8.418 0.077
	Disagree	40 (13.3)	12 (11)		44 (13.6)	8 (9.3)		22 (13.8)	8 (13.3)	22 (11.6)		48 (14.7)	4 (4.8)	
	Neutral	58 (19.3)	16 (14.7)		62 (19.2)	12 (14)		26 (16.2)	12 (20)	36 (19)		61 (18.7)	13 (15.7)	
	Agree	92 (30.7)	45 (41.3)		105 (32.5)	32 (37.2)		62 (38.8)	19 (31.7)	56 (29.6)		109 (33.4)	28 (33.7)	
	Strongly agree	96 (32)	35 (32.1)		99 (30.7)	32 (37.2)		45 (28.1)	20 (33.3)	66 (34.9)		97 (29.8)	34 (41)	
**Online learning can cause isolation and lack of belonging and support**	Strongly disagree	17 (5.7)	3 (2.8)	4.225 0.376	14 (4.3)	6 (7)	5.864 0.21	5 (3.1)	1 (1.7)	14 (7.4)	8.086 0.425	15 (4.6)	5 (6)	3.067 0.547
	Disagree	45 (15)	16 (14.7)		50 (15.5)	11 (12.8)		25 (15.6)	11 (18.3)	25 (13.2)		53 (16.3)	8 (9.6)	
	Neutral	79 (26.3)	22 (20.2)		85 (26.3)	16 (18.6)		42 (26.2)	15 (25)	44 (23.3)		82 (25.2)	19 (22.9)	
	Agree	99 (33)	45 (41.3)		106 (32.8)	38 (44.2)		60 (37.5)	22 (36.7)	62 (32.8)		111 (34)	33 (39.8)	
	Strongly agree	60 (20)	23 (21.1)		68 (21.1)	15 (17.4)		28 (17.5)	11 (18.3)	44 (23.3)		65 (19.9)	18 (21.7)	

#### Impact on learning

The majority of participants believed online classes had minimal impact on developing students’ overall personalities and communication skills. Students with limited e-learning skills (50.6%) likened online learning to watching YouTube lectures (*p* = 0.061), implying a passive learning experience. This suggests online learning may not be as effective as traditional classroom settings in fostering these crucial soft skills.

Despite concerns about suitability and learning environment, a significant portion of participants, particularly undergraduates (42.7%, *p* = 0.001), expressed satisfaction with the topics covered and the instructors’ efforts in the online environment. This highlights a potential disconnect between student concerns and their actual experience with well-designed online learning.

Most undergraduates strongly agreed (49.2%, *p* = 0.04) that online teaching/learning is extremely useful during disasters such as the coronavirus pandemic (Table 3). This emphasizes the potential of online learning as a contingency measure for educational continuity during unforeseen circumstances.

**Table 3:** Comparison of online verses class room teaching by the students who participated in the study during the Covid 19 pandemic in India

	Categories	Age		Chi square *P* value	Course of study		Chi square *P* value	Place			Chi square *P* value	E learning skills		Chi square *P* value
		18–21 years (N %)	> 21 years (N %)		UG (%)	PG (N %)		Village (N %)	Town (N %)	City (N %)		yes (N %)	no (N %)	
I prefer the classroom teaching over online teaching because there is more involvement with teachers and classmates. This is not there in online teaching	Strongly disagree	12 (4)	3 (2.8)	3.629 0.459	12 (3.7)	3 (3.5)	1.792 0.774	7 (4.4)	4 (6.7)	4 (2.1)	5.655 0.686	11 (3.4)	4 (4.8)	1.144 0.887
	Disagree	15 (5)	10 (9.2)		18 (5.6)	7 (8.1)		7 (4.4)	5 (8.3)	13 (6.9)		21 (6.4)	4 (4.8)	
	Neutral	33 (11)	15 (13.8)		39 (12.1)	9 (10.5)		20 (12.5)	8 (13.3)	20 (10.6)		40 (12.3)	8 (9.6)	
	Agree	133 (44.3)	47 (43.1)		139 (43)	41 (47.7)		69 (43.1)	26 (43.3)	85 (45)		143 (43.9)	37 (44.6)	
	Strongly agree	107 (35.7)	34 (31.2)		115 (35.6)	26 (30.2)		57 (35.6)	17 (28.3)	67 (35.4)		111 (34)	30 (36.1)	
I am very much used to classroom studying and feel online teaching/learning is not very useful.	Strongly disagree	16 (5.3)	5 (4.6)	4.047 0.4	15 (4.6)	6 (7)	5.587 0.232	7 (4.4)	3 (5)	11 (5.8)	5.068 0.75	15 (4.6)	6 (7.2)	9.729 **0.045**
	Disagree	54 (18)	29 (26.6)		66 (20.4)	17 (19.8)		29 (18.1)	14 (23.3)	40 (21.2)		72 (22.1)	11 (13.3)	
	Neutral	63 (21)	18 (16.5)		71 (22)	10 (11.6)		29 (18.1)	15 (25)	37 (19.6)		70 (21.5)	11 (13.3)	
	Agree	70 (23.3)	25 (22.9)		74 (22.9)	21 (24.4)		36 (22.5)	12 (20)	47 (24.9)		68 (20.9)	27 (32.5)	
	Strongly agree	97 (32.3)	32 (29.4)		97 (30)	32 (37.2)		59 (36.9)	16 (26.7)	54 (28.6)		101 (31)	28 (33.7)	
Unlike in classroom, online teaching will not help develop a student’s overall personality and communication	Strongly disagree	10 (3.3)	5 (4.6)	0.742 0.946	11 (3.4)	4 (4.7)	0.39 0.983	4 (2.5)	3 (5)	8 (4.2)	3.36 0.91	11 (3.4)	4 (4.8)	4.352 0.36
	Disagree	40 (13.3)	14 (12.8)		42 (13)	12 (14)		21 (13.1)	6 (10)	27 (14.3)		48 (14.7)	6 (7.2)	
	Neutral	43 (14.3)	17 (15.6)		48 (14.9)	12 (14)		27 (16.9)	10 (16.7)	23 (12.2)		45 (13.8)	15 (18.1)	
	Agree	95 (31.7)	36 (33)		104 (32.2)	27 (31.4)		52 (32.5)	19 (31.7)	60 (31.7)		102 (31.3)	29 (34.9)	
	Strongly agree	112 (37.3)	37 (33.9)		118 (36.5)	31 (36)		56 (35)	22 (36.7)	71 (37.6)		120 (36.8)	29 (34.9)	
Online learning is very similar to watching you tube lectures. Only difference is, your teacher is taking class	Strongly disagree	10 (3.3)	3 (2.8)	2.54 0.638	9 (2.8)	4 (4.7)	5.463 0.243	5 (3.1)	1 (1.7)	7 (3.7)	12.757 0.12	10 (3.1)	3 (3.6)	9.016 0.061
	Disagree	44 (14.7)	16 (14.7)		52 (16.1)	8 (9.3)		22 (13.8)	4 (6.7)	34 (18)		55 (16.9)	5 (6)	
	Neutral	52 (17.3)	14 (12.8)		56 (17.3)	10 (11.6)		34 (21.2)	8 (13.3)	24 (12.7)		56 (17.2)	10 (12)	
	Agree	61 (20.3)	29 (26.6)		68 (21.1)	22 (25.6)		29 (18.1)	19 (31.7)	42 (22.2)		67 (20.6)	23 (27.7)	
	Strongly agree	133 (44.3)	47 (43.1)		138 (42.7)	42 (48.8)		70 (43.8)	28 (46.7)	82 (43.4)		138 (42.3)	42 (50.6)	
Online teaching/ learning is extremely useful during calamities like the coronavirus pandemic	Strongly disagree	12 (4)	4 (3.7)	5.098 0.277	10 (3.1)	6 (7)	10.03 **0.04**	6 (3.8)	2 (3.3)	8 (4.2)	9.166 0.329	11 (3.4)	5 (6)	7.867 0.097
	Disagree	11 (3.7)	3 (2.8)		11 (3.4)	3 (3.5)		7 (4.4)	3 (5)	4 (2.1)		9 (2.8)	5 (6)	
	Neutral	31 (10.3)	19 (17.4)		34 (10.5)	16 (18.6)		24 (15)	7 (11.7)	19 (10.1)		35 (10.7)	15 (18.1)	
	Agree	101 (33.7)	40 (36.7)		109 (33.7)	32 (37.2)		43 (26.9)	22 (36.7)	76 (40.2)		118 (36.2)	23 (27.7)	
	Strongly agree	145 (48.3)	43 (39.4)		159 (49.2)	29 (33.7)		80 (50)	26 (43.3)	82 (43.4)		153 (46.9)	35 (42.2)	

Overall, student perceptions regarding the suitability and learning outcomes of online learning were mixed. While some found it beneficial for specific situations and expressed satisfaction with well-designed online courses, concerns existed about its effectiveness in fostering a sense of community, developing soft skills, and replicating the interactive nature of traditional classroom settings.

### Teacher perceptions

#### Advantages of online teaching

A considerable number of teachers (40%) viewed online classes as a more adaptable alternative to traditional classroom settings. Similarly, nearly half (49.2%) expressed this view regarding student accessibility. Additionally, a significant majority (59.8%) believed online teaching offered students improved 24/7 access to learning materials.

#### Challenges of online teaching

Teachers reported a significant decrease (50%) in the use of standardized coursework compared to traditional classrooms. While they strongly disagreed (38%) that online teaching eliminates the need for proper lesson planning, they overwhelmingly felt it hindered creating a good interactive environment with students (87%). Furthermore, teachers believed students were less likely to ask questions in an online setting (61%) compared to a physical classroom. However, most teachers (79%) appreciated the elimination of physical travel associated with online teaching.

#### Suitability and effectiveness

In terms of learner level, online teaching was perceived as more suitable for advanced learners (58%) than beginners. Teachers also believed online teaching was better suited for theory-based subjects (87%) compared to laboratory-based ones. Opinions were divided regarding the optimal use of online teaching for knowledge transfer, with 39% disagreeing and 24% remaining neutral. The teachers concurred that online teaching was a valuable tool during crises like the COVID-19 pandemic, but they generally preferred face-to-face teaching under normal circumstances. Data pertaining to these findings is presented in Table 4.

**Table 4:** Opinion of teachers regarding Online teaching during the Covid 19 pandemic in India

Subject related questions	Choices	Total Number *n* = 132 N(%)	Professional position			Chi square/Fishers exact *P* value
			**Assistant Professor**	**Associate Professor**	Professor	
When compared to conventional class room teaching I feel online classes are flexible for teachers	Strongly Agree	5 (3.8)	1 (2.9)	1 (2.8)	3 (4.9)	3.411 0.929
	Agree	53 (40.2)	16 (45.7)	16 (44.4)	21 (34.4)	
	Neither Agree Nor Disagree	23 (17.4)	7 (20)	5 (13.9)	11 (18)	
	Disagree	42 (31.8)	10 (28.6)	12 (33.3)	20 (32.8)	
	Strongly Disagree	9 (6.8)	1 (2.9)	2 (5.6)	6 (9.8)	
When compared to conventional class room teaching I feel online classes are flexible for students	Strongly Agree	16 (12.1)	5 (14.3)	5 (13.9)	6 (9.8)	7.832 0.448
	Agree	65 (49.2)	21 (60)	17 (47.2)	27 (44.3)	
	Neither Agree Nor Disagree	19 (14.4)	2 (5.7)	8 (22.2)	9 (14.8)	
	Disagree	23 (17.4)	6 (17.1)	4 (11.1)	13 (21.3)	
	Strongly Disagree	9 (6.8)	1 (2.9)	2 (5.6)	6 (9.8)	
When compared to conventional class room teaching I feel student has 24/7 access to online teaching materials	Strongly Agree	6 (4.5)	0 (0)	1 (2.8)	5 (8.2)	9.023 0.290
	Agree	79 (59.8)	18 (51.4)	24 (66.7)	37 (60.7)	
	Neither Agree Nor Disagree	20 (15.2)	7 (20)	7 (19.4)	6 (9.8)	
	Disagree	24 (18.2)	9 (25.7)	4 (11.1)	11 (18)	
	Strongly Disagree	3 (2.3)	1 (2.9)	0 (0)	2 (3.3)	
When compared to conventional class room teaching I feel the standardized coursework is reduced for the students	Strongly Agree	14 (10.6)	3 (8.6)	2 (5.6)	9 (14.8)	10.613 0.192
	Agree	66 (50)	14 (40)	20 (55.6)	32 (52.5)	
	Neither Agree Nor Disagree	19 (14.4)	6 (17.1)	8(22.2)	5(8.2)	
	Disagree	29 (22)	9 (25.7)	6 (16.7)	14 (23)	
	Strongly Disagree	4 (3)	3 (8.6)	0 (0)	1 (1.6)	
When compared to conventional class room teaching I feel there is no need for lesson planning	Strongly Agree	0 (0)	0 (0)	0 (0)	0 (0)	3.167 0.819
	Agree	3 (2.3)	2 (5.7)	0 (0)	1 (1.6)	
	Neither Agree Nor Disagree	9 (6.8)	2 (5.7)	3 (8.3)	4 (6.6)	
	Disagree	69 (52.3)	16 (45.7)	20 (55.6)	33 (54.1)	
	Strongly Disagree	51 (38.6)	15 (42.9)	13 (36.1)	23 (37.7)	
When compared to conventional class room teaching I feel online classes helps better interaction with students	Strongly Agree	1 (0.8)	0 (0)	1 (2.8)	0 (0)	5.537 0.742
	Agree	6 (4.5)	3 (8.6)	1 (2.8)	2 (3.3)	
	Neither Agree Nor Disagree	10 (7.6)	2 (5.7)	2 (5.6)	6 (9.8)	
	Disagree	58 (43.9)	15 (42.9)	14 (38.9)	29 (47.5)	
	Strongly Disagree	57 (43.2)	15 (42.9)	18 (50)	24 (39.3)	
When compared to conventional class room teaching I feel students are more comfortable to ask questions	Strongly Agree	2 (1.5)	1 (2.9)	0 (0)	1 (1.6)	9.449 0.263
	Agree	30 (22.7)	10 (28.6)	8 (22.2)	12 (19.7)	
	Neither Agree Nor Disagree	20 (15.2)	4 (11.4)	9 (25)	7 (11.5)	
	Disagree	53 (40.2)	13 (37.1)	16 (44.4)	24 (39.3)	
	Strongly Disagree	27 (20.5)	7 (20)	3 (8.3)	17 (27.9)	
When compared to conventional class room teaching I feel teacher can conduct classes without physical travel	Strongly Agree	27 (20.5)	8 (22.9)	6 (16.7)	13 (21.3)	4.303 0.866
	Agree	80 (60.6)	21 (60)	25 (69.4)	34 (55.7)	
	Neither Agree Nor Disagree	18 (13.6)	5 (14.3)	3 (8.3)	10 (16.4)	
	Disagree	4 (3)	0 (0)	1 (2.8)	3 (4.9)	
	Strongly Disagree	3 (2.3)	1 (2.9)	1 (2.8)	1 (1.6)	
When compared to conventional class room teaching I feel learners have opportunity to hear lectures of experts	Strongly Agree	24 (18.2)	7 (20)	7 (19.4)	10 (16.4)	5.568 0.705
	Agree	80 (60.6)	21 (60)	24 (66.7)	35 (57.4)	
	Neither Agree Nor Disagree	16 (12.1)	3 (8.6)	2(5.6)	11 (18)	
	Disagree	9 (6.8)	3 (8.6)	3 (8.3)	3 (4.9)	
	Strongly Disagree	3 (2.3)	1 (2.9)	0 (0)	2 (3.3)	
When compared to conventional class room teaching I feel online classes is useful only for advanced learning and not for beginners	Strongly Agree	23 (17.4)	7 (20)	6 (16.7)	10 (16.4)	6.239 0.617
	Agree	54 (40.9)	15 (42.9)	14 (38.9)	25 (41)	
	Neither Agree Nor Disagree	21 (15.9)	8 (22.9)	6(16.7)	7 (11.5)	
	Disagree	31 (23.5)	4 (11.4)	9 (25)	18 (29.5)	
	Strongly Disagree	3 (2.3)	1 (2.9)	1(2.8)	1 (1.6)	
When compared to conventional class room teaching I feel online classes is useful only for theory based topics and not for laboratory emphasized topics where practical skills are to be learnt from a teacher	Strongly Agree	74 (56.1)	18 (51.4)	21 (58.3)	35 (57.4)	4.263 0.897
	Agree	49 (37.1)	13(37.1)	14 (38.9)	22 (36.1)	
	Neither Agree Nor Disagree	3 (2.3)	1 (2.9)	0 (0)	2 (3.3)	
	Disagree	3 (2.3)	2 (5.7)	0 (0)	1 (1.6)	
	Strongly Disagree	3 (2.3)	1 (2.9)	1 (2.8)	1 (1.6)	
When compared to conventional class room teaching I feel there is optimal knowledge transfer from teacher to student in online teaching?	Strongly Agree	6 (4.5)	1 (2.9)	3 (8.3)	2 (3.3)	10.022 0.243
	Agree	30 (22.7)	8 (22.9)	5 (13.9)	17 (27.9)	
	Neither Agree Nor Disagree	32 (24.2)	5 (14.3)	14 (38.9)	13 (21.3)	
	Disagree	52 (39.4)	16 (45.7)	12 (33.3)	24 (39.3)	
	Strongly Disagree	12 (9.1)	5 (14.3)	2 (5.6)	5 (8.2)	
Online classes are useful only during natural calamities and pandemics not on a regular basis.	Strongly Agree	53 (40.2)	16 (45.7)	11 (30.6)	26 (42.6)	10.772 0.182
	Agree	46 (34.8)	12 (34.3)	14 (38.9)	20 (32.8)	
	Neither Agree Nor Disagree	12 (9.1)	0 (0)	5 (13.9)	7 (11.5)	
	Disagree	17 (12.9)	5 (14.3)	4 (11.1)	8 (13.1)	
	Strongly Disagree	4 (3)	2 (5.7)	2 (5.6)	0 (0)	

## Discussion

The COVID-19 pandemic necessitated a rapid shift to online learning platforms in dental education globally, including India. While this transition aimed to maintain educational continuity [12], it presented unique challenges for a country grappling with limited internet infrastructure [13]. Existing disparities in access were exacerbated by the pandemic’s suddenness, highlighting the need for innovative solutions tailored to the Indian context [14].

Our study aimed to understand the perceptions and challenges of online dental education among students and educators. Our findings resonate with existing research, highlighting both the advantages and limitations of online learning. Similar to previous studies, both students and educators in our research acknowledged the benefits of flexibility, improved online teaching skills, and efficient time management [15–19]. Additionally, the significant role of online resources and social media platforms in fostering learning and interaction, as emphasized by Azer et al. (2023) and Wimardhani et al. (2023), was evident in our findings [17, 19].

This research explored factors influencing student satisfaction with online learning. Consistent with Shaheen et al. (2023), our results indicated higher satisfaction among younger students (aged 18–21) and those with stronger e-learning skills, suggesting a correlation with technological comfort [18]. However, unlike Schlenz et al. (2023) who observed a general preference for online learning, our study did not find significant variations in satisfaction based on age, location, or field of study [15]. Notably, students with advanced e-learning skills reported higher dissatisfaction with internet connectivity and speed, suggesting a potential link between heightened expectations and increased frustration with technical limitations. This aligns with observations made by Pratheebha & Jayaraman (2022), Chang et al. (2021), and Wimardhani et al. (2023) regarding student challenges in online learning environments [16, 19, 20]. While acknowledging the quality of online instruction, many students in our study, similar to those in Chang et al. (2021), expressed feelings of isolation and a preference for the interactive elements of traditional classroom settings [20].

The transition to online learning presented specific challenges in dental education, particularly for subjects requiring hands-on experience. Deery (2020) emphasizes the need for dental schools to adapt their curricula and policies to incorporate effective distance learning methods [21]. Our research reinforces this notion by highlighting the importance of a strong educator-student connection for successful online learning. In the face of these challenges, educators and administrators remain committed to creating a conducive learning environment that prioritizes adaptability.

Online learning platforms offer unique advantages. E-learning technologies empower learners to personalize their learning pace, sequence, and content, leading to improved engagement [22]. Additionally, recorded online lectures provide flexibility for students to access learning materials at their convenience [23]. Our research, building upon prior work by Pham (2022) and Chang et al. (2021), demonstrated a weaker association between peer-to-peer interactions and student satisfaction, consistent with findings in other online learning environments [20, 24].

Several factors influence the success of online education, including educator willingness to share content online, student capacity for online learning, and the quality of available digital resources [25]. Political, economic, and cultural factors also significantly influence the transition from traditional to online learning [25]. While acknowledging the potential for academic collaboration and remote work, many educators recognize the opportunity to integrate blended learning models into future curriculum development [26].

“Internet self-efficacy” – an individual’s confidence in navigating online tasks – plays a crucial role in online learning success [27]. In India, internet connectivity disparities between urban and rural areas present a challenge for both students and teachers. These connectivity issues, along with software problems and audio/video functionalities, can disrupt learning and create a frustrating experience. Institutions can mitigate these challenges by offering comprehensive internet skills training to enhance students’ and educators’ internet self-efficacy before implementing online courses [24]. However, the pandemic’s swift implementation of remote learning may have limited the availability of such training protocols.

### Challenges and innovations in clinical skills development

While online learning offers numerous advantages, it presents unique challenges in dental education, particularly for subjects requiring hands-on clinical experience with patients. The absence of direct patient interaction remains a significant hurdle [21]. However, several institutions are actively addressing this limitation by adopting diverse e-learning tools like flash multimedia, digitized images, virtual patient simulations, and virtual reality (VR) simulators. Research has shown the effectiveness of these tools in teaching various clinical skills, including examination, palpation, surgical procedures, and resuscitation [28]. Notably, VR simulators have been found to be equally effective as live patient interactions in achieving learning objectives, offering a promising solution for overcoming limitations in online dental education.

### The rise of virtual interaction and blended learning models

The COVID-19 pandemic has significantly transformed the educational landscape in dental education by introducing virtual teaching platforms. This shift has reshaped interactions between educators and students, impacting how they learn and assess progress. The rise of web-based resources has facilitated the emergence of innovative virtual interaction methods, such as student-patient simulations and peer mentoring programs. Research suggests these methods can be effective in enhancing medical students’ knowledge and psychological well-being [29]. However, this transition to online learning has also encountered obstacles, including technical difficulties, privacy concerns, reduced student engagement, and potential exacerbation of mental health issues due to social isolation [27, 29, 30].

### Optimizing blended learning for future dental education

The unique circumstances of the COVID-19 pandemic have highlighted the importance of exploring student preferences and technical challenges to optimize blended learning models in dental education. By addressing the diverse needs of students and effectively integrating online and offline learning components, educators can foster successful learning outcomes in an ever-evolving educational environment [30]. This research underscores the multifaceted nature of online dental education and emphasizes the necessity for collaborative efforts to leverage its advantages while mitigating limitations.

### Building educational resilience and adaptability

The significance of these studies extends beyond immediate pandemic adaptations. They contribute to a broader understanding of learning adaptations, hybrid learning environments, digital literacy, pedagogical innovation, mental health and well-being, policy implications, and the continuous enhancement of educational practices [30]. Reflecting on experiences and lessons learned during the pandemic can assist educational institutions in refining their teaching and learning approaches, ensuring greater resilience and adaptability in the face of future challenges [29]. Therefore, the insights from these studies offer valuable guidance for shaping the future of dental education and broader educational practices in a post-pandemic world.

### Limitations and future research directions

We acknowledge limitations in our study. Employing random sampling methods in future research would be crucial to draw more widely applicable conclusions regarding perceptions and challenges in online dental education in India. Additionally, we recognize the challenges associated with relying on self-reported data, including potential social desirability bias. While acknowledging these limitations, our study adopted a people-centred approach, employing a diverse questionnaire, contextual analysis, and insightful techniques to gain a profound understanding of participants’ experiences with digital instruction. However, these limitations underscore the need for further exploration, particularly in understanding the potential misalignment between outcomes of digital and in-person events from instructors’ perspectives. This area warrants additional research through targeted interviews, subgroup analyses, and consideration of contextual factors, aiming to enhance our understanding of effective teaching modes and benefitting student learning outcomes.

In conclusion, the COVID-19 pandemic has accelerated the adoption of online and virtual teaching platforms in dental education, offering both opportunities and challenges. By exploring student preferences and addressing technical obstacles, educators can refine blended learning models to better cater to diverse student needs. The insights gleaned from pandemic experiences provide valuable direction for bolstering the resilience and adaptability of educational practices in a post-pandemic era.

### Supplementary Information

**Supplementary Material 1.**

## Data Availability

The datasets used and/or analysed throughout the current investigation are attainable from the corresponding author following a justifiable request.
