# Supplementary material for: Perceptions and challenges of online teaching and learning amidst the COVID-19 pandemic in India: a cross-sectional study with dental students and teachers
Source: BMC Med Educ. 2024 Jun 6;24:637. doi: 10.1186/s12909-024-05340-2 (PMC11157739; doi:10.1186/s12909-024-05340-2)
Supplement: Supplementary file 1 — Supplementary Material 1. [file 12909_2024_5340_MOESM1_ESM.doc]

**Crosstabs**

[DataSet3] C:\Users\NEEVAN\Desktop\Untitled4 students aditya.sav

**1. I was satisfied with online teaching by my teacher * Age**

| **Crosstab** | | | | | |
| --- | --- | --- | --- | --- | --- |
|  | | | Age | | Total |
|  |  |  | 1 | 2 |  |
| 1. I was satisfied with online teaching by my teacher | 1 | Count | 20 | 7 | 27 |
|  |  | % within Age | 6.7% | 6.4% | 6.6% |
|  | 2 | Count | 37 | 13 | 50 |
|  |  | % within Age | 12.3% | 11.9% | 12.2% |
|  | 3 | Count | 61 | 33 | 94 |
|  |  | % within Age | 20.3% | 30.3% | 23.0% |
|  | 4 | Count | 131 | 42 | 173 |
|  |  | % within Age | 43.7% | 38.5% | 42.3% |
|  | 5 | Count | 51 | 14 | 65 |
|  |  | % within Age | 17.0% | 12.8% | 15.9% |
| Total | | Count | 300 | 109 | 409 |
|  |  | % within Age | 100.0% | 100.0% | 100.0% |

| **Chi-Square Tests** | | | | | | |
| --- | --- | --- | --- | --- | --- | --- |
|  | Value | df | Asymp. Sig. (2-sided) | Exact Sig. (2-sided) | Exact Sig. (1-sided) | Point Probability |
| Pearson Chi-Square | 4.824^a^ | 4 | .306 | .308 |  |  |
| Likelihood Ratio | 4.680 | 4 | .322 | .331 |  |  |
| Fisher's Exact Test | 4.654 |  |  | .324 |  |  |
| Linear-by-Linear Association | 1.040^b^ | 1 | .308 | .310 | .166 | .024 |
| N of Valid Cases | 409 |  |  |  |  |  |

| a. 0 cells (0.0%) have expected count less than 5. The minimum expected count is 7.20. |
| --- |
| b. The standardized statistic is -1.020. |

**1. I was satisfied with online teaching by my teacher * Course of study**

| **Crosstab** | | | | | |
| --- | --- | --- | --- | --- | --- |
|  | | | Course of study | | Total |
|  |  |  | 1 | 2 |  |
| 1. I was satisfied with online teaching by my teacher | 1 | Count | 20 | 7 | 27 |
|  |  | % within Course of study | 6.2% | 8.1% | 6.6% |
|  | 2 | Count | 30 | 20 | 50 |
|  |  | % within Course of study | 9.3% | 23.3% | 12.2% |
|  | 3 | Count | 70 | 24 | 94 |
|  |  | % within Course of study | 21.7% | 27.9% | 23.0% |
|  | 4 | Count | 148 | 25 | 173 |
|  |  | % within Course of study | 45.8% | 29.1% | 42.3% |
|  | 5 | Count | 55 | 10 | 65 |
|  |  | % within Course of study | 17.0% | 11.6% | 15.9% |
| Total | | Count | 323 | 86 | 409 |
|  |  | % within Course of study | 100.0% | 100.0% | 100.0% |

| **Chi-Square Tests** | | | | | | |
| --- | --- | --- | --- | --- | --- | --- |
|  | Value | df | Asymp. Sig. (2-sided) | Exact Sig. (2-sided) | Exact Sig. (1-sided) | Point Probability |
| Pearson Chi-Square | 18.130^a^ | 4 | .001 | .001 |  |  |
| Likelihood Ratio | 16.968 | 4 | .002 | .002 |  |  |
| Fisher's Exact Test | 17.144 |  |  | .002 |  |  |
| Linear-by-Linear Association | 11.565^b^ | 1 | .001 | .001 | .000 | .000 |
| N of Valid Cases | 409 |  |  |  |  |  |

| a. 0 cells (0.0%) have expected count less than 5. The minimum expected count is 5.68. |
| --- |
| b. The standardized statistic is -3.401. |

**1. I was satisfied with online teaching by my teacher * Place**

| **Crosstab** | | | | | | |
| --- | --- | --- | --- | --- | --- | --- |
|  | | | Place | | | Total |
|  |  |  | 1 | 2 | 3 |  |
| 1. I was satisfied with online teaching by my teacher | 1 | Count | 6 | 6 | 15 | 27 |
|  |  | % within Place | 3.8% | 10.0% | 7.9% | 6.6% |
|  | 2 | Count | 27 | 6 | 17 | 50 |
|  |  | % within Place | 16.9% | 10.0% | 9.0% | 12.2% |
|  | 3 | Count | 34 | 20 | 40 | 94 |
|  |  | % within Place | 21.2% | 33.3% | 21.2% | 23.0% |
|  | 4 | Count | 73 | 21 | 79 | 173 |
|  |  | % within Place | 45.6% | 35.0% | 41.8% | 42.3% |
|  | 5 | Count | 20 | 7 | 38 | 65 |
|  |  | % within Place | 12.5% | 11.7% | 20.1% | 15.9% |
| Total | | Count | 160 | 60 | 189 | 409 |
|  |  | % within Place | 100.0% | 100.0% | 100.0% | 100.0% |

| **Chi-Square Tests** | | | | | |
| --- | --- | --- | --- | --- | --- |
|  | Value | df | Asymp. Sig. (2-sided) | Exact Sig. (2-sided) | Exact Sig. (1-sided) |
| Pearson Chi-Square | 16.624^a^ | 8 | .034 | .^b^ |  |
| Likelihood Ratio | 16.395 | 8 | .037 | .^b^ |  |
| Fisher's Exact Test | .^b^ |  |  | .^b^ |  |
| Linear-by-Linear Association | .941 | 1 | .332 | .^b^ | .^b^ |
| N of Valid Cases | 409 |  |  |  |  |

| a. 1 cells (6.7%) have expected count less than 5. The minimum expected count is 3.96. |
| --- |
| b. Cannot be computed because there is insufficient memory. |

**1. I was satisfied with online teaching by my teacher * E learning skills**

| **Crosstab** | | | | | |
| --- | --- | --- | --- | --- | --- |
|  | | | E learning skills | | Total |
|  |  |  | 1 | 2 |  |
| 1. I was satisfied with online teaching by my teacher | 1 | Count | 13 | 14 | 27 |
|  |  | % within E learning skills | 4.0% | 16.9% | 6.6% |
|  | 2 | Count | 38 | 12 | 50 |
|  |  | % within E learning skills | 11.7% | 14.5% | 12.2% |
|  | 3 | Count | 75 | 19 | 94 |
|  |  | % within E learning skills | 23.0% | 22.9% | 23.0% |
|  | 4 | Count | 142 | 31 | 173 |
|  |  | % within E learning skills | 43.6% | 37.3% | 42.3% |
|  | 5 | Count | 58 | 7 | 65 |
|  |  | % within E learning skills | 17.8% | 8.4% | 15.9% |
| Total | | Count | 326 | 83 | 409 |
|  |  | % within E learning skills | 100.0% | 100.0% | 100.0% |

| **Chi-Square Tests** | | | | | | |
| --- | --- | --- | --- | --- | --- | --- |
|  | Value | df | Asymp. Sig. (2-sided) | Exact Sig. (2-sided) | Exact Sig. (1-sided) | Point Probability |
| Pearson Chi-Square | 21.298^a^ | 4 | .000 | .000 |  |  |
| Likelihood Ratio | 18.413 | 4 | .001 | .001 |  |  |
| Fisher's Exact Test | 18.419 |  |  | .001 |  |  |
| Linear-by-Linear Association | 15.625^b^ | 1 | .000 | .000 | .000 | .000 |
| N of Valid Cases | 409 |  |  |  |  |  |

| a. 0 cells (0.0%) have expected count less than 5. The minimum expected count is 5.48. |
| --- |
| b. The standardized statistic is -3.953. |

**2. I was satisfied with discussion on the topic * Age**

| **Crosstab** | | | | | |
| --- | --- | --- | --- | --- | --- |
|  | | | Age | | Total |
|  |  |  | 1 | 2 |  |
| 2. I was satisfied with discussion on the topic | 1 | Count | 11 | 5 | 16 |
|  |  | % within Age | 3.7% | 4.6% | 3.9% |
|  | 2 | Count | 51 | 11 | 62 |
|  |  | % within Age | 17.0% | 10.1% | 15.2% |
|  | 3 | Count | 55 | 29 | 84 |
|  |  | % within Age | 18.3% | 26.6% | 20.5% |
|  | 4 | Count | 145 | 54 | 199 |
|  |  | % within Age | 48.3% | 49.5% | 48.7% |
|  | 5 | Count | 38 | 10 | 48 |
|  |  | % within Age | 12.7% | 9.2% | 11.7% |
| Total | | Count | 300 | 109 | 409 |
|  |  | % within Age | 100.0% | 100.0% | 100.0% |

| **Chi-Square Tests** | | | | | | |
| --- | --- | --- | --- | --- | --- | --- |
|  | Value | df | Asymp. Sig. (2-sided) | Exact Sig. (2-sided) | Exact Sig. (1-sided) | Point Probability |
| Pearson Chi-Square | 6.209^a^ | 4 | .184 | .184 |  |  |
| Likelihood Ratio | 6.329 | 4 | .176 | .185 |  |  |
| Fisher's Exact Test | 6.198 |  |  | .181 |  |  |
| Linear-by-Linear Association | .004^b^ | 1 | .950 | .956 | .495 | .044 |
| N of Valid Cases | 409 |  |  |  |  |  |

| a. 1 cells (10.0%) have expected count less than 5. The minimum expected count is 4.26. |
| --- |
| b. The standardized statistic is -.063. |

**2. I was satisfied with discussion on the topic * Course of study**

| **Crosstab** | | | | | |
| --- | --- | --- | --- | --- | --- |
|  | | | Course of study | | Total |
|  |  |  | 1 | 2 |  |
| 2. I was satisfied with discussion on the topic | 1 | Count | 12 | 4 | 16 |
|  |  | % within Course of study | 3.7% | 4.7% | 3.9% |
|  | 2 | Count | 43 | 19 | 62 |
|  |  | % within Course of study | 13.3% | 22.1% | 15.2% |
|  | 3 | Count | 60 | 24 | 84 |
|  |  | % within Course of study | 18.6% | 27.9% | 20.5% |
|  | 4 | Count | 168 | 31 | 199 |
|  |  | % within Course of study | 52.0% | 36.0% | 48.7% |
|  | 5 | Count | 40 | 8 | 48 |
|  |  | % within Course of study | 12.4% | 9.3% | 11.7% |
| Total | | Count | 323 | 86 | 409 |
|  |  | % within Course of study | 100.0% | 100.0% | 100.0% |

| **Chi-Square Tests** | | | | | | |
| --- | --- | --- | --- | --- | --- | --- |
|  | Value | df | Asymp. Sig. (2-sided) | Exact Sig. (2-sided) | Exact Sig. (1-sided) | Point Probability |
| Pearson Chi-Square | 10.593^a^ | 4 | .032 | .031 |  |  |
| Likelihood Ratio | 10.361 | 4 | .035 | .041 |  |  |
| Fisher's Exact Test | 10.611 |  |  | .028 |  |  |
| Linear-by-Linear Association | 7.120^b^ | 1 | .008 | .008 | .005 | .001 |
| N of Valid Cases | 409 |  |  |  |  |  |

| a. 1 cells (10.0%) have expected count less than 5. The minimum expected count is 3.36. |
| --- |
| b. The standardized statistic is -2.668. |

**2. I was satisfied with discussion on the topic * Place**

| **Crosstab** | | | | | | |
| --- | --- | --- | --- | --- | --- | --- |
|  | | | Place | | | Total |
|  |  |  | 1 | 2 | 3 |  |
| 2. I was satisfied with discussion on the topic | 1 | Count | 2 | 4 | 10 | 16 |
|  |  | % within Place | 1.2% | 6.7% | 5.3% | 3.9% |
|  | 2 | Count | 27 | 10 | 25 | 62 |
|  |  | % within Place | 16.9% | 16.7% | 13.2% | 15.2% |
|  | 3 | Count | 41 | 19 | 24 | 84 |
|  |  | % within Place | 25.6% | 31.7% | 12.7% | 20.5% |
|  | 4 | Count | 80 | 22 | 97 | 199 |
|  |  | % within Place | 50.0% | 36.7% | 51.3% | 48.7% |
|  | 5 | Count | 10 | 5 | 33 | 48 |
|  |  | % within Place | 6.2% | 8.3% | 17.5% | 11.7% |
| Total | | Count | 160 | 60 | 189 | 409 |
|  |  | % within Place | 100.0% | 100.0% | 100.0% | 100.0% |

| **Chi-Square Tests** | | | | | |
| --- | --- | --- | --- | --- | --- |
|  | Value | df | Asymp. Sig. (2-sided) | Exact Sig. (2-sided) | Exact Sig. (1-sided) |
| Pearson Chi-Square | 29.217^a^ | 8 | .000 | .^b^ |  |
| Likelihood Ratio | 30.599 | 8 | .000 | .^b^ |  |
| Fisher's Exact Test | .^b^ |  |  | .^b^ |  |
| Linear-by-Linear Association | 3.395 | 1 | .065 | .^b^ | .^b^ |
| N of Valid Cases | 409 |  |  |  |  |

| a. 1 cells (6.7%) have expected count less than 5. The minimum expected count is 2.35. |
| --- |
| b. Cannot be computed because there is insufficient memory. |

**2. I was satisfied with discussion on the topic * E learning skills**

| **Crosstab** | | | | | |
| --- | --- | --- | --- | --- | --- |
|  | | | E learning skills | | Total |
|  |  |  | 1 | 2 |  |
| 2. I was satisfied with discussion on the topic | 1 | Count | 8 | 8 | 16 |
|  |  | % within E learning skills | 2.5% | 9.6% | 3.9% |
|  | 2 | Count | 47 | 15 | 62 |
|  |  | % within E learning skills | 14.4% | 18.1% | 15.2% |
|  | 3 | Count | 61 | 23 | 84 |
|  |  | % within E learning skills | 18.7% | 27.7% | 20.5% |
|  | 4 | Count | 167 | 32 | 199 |
|  |  | % within E learning skills | 51.2% | 38.6% | 48.7% |
|  | 5 | Count | 43 | 5 | 48 |
|  |  | % within E learning skills | 13.2% | 6.0% | 11.7% |
| Total | | Count | 326 | 83 | 409 |
|  |  | % within E learning skills | 100.0% | 100.0% | 100.0% |

| **Chi-Square Tests** | | | | | | |
| --- | --- | --- | --- | --- | --- | --- |
|  | Value | df | Asymp. Sig. (2-sided) | Exact Sig. (2-sided) | Exact Sig. (1-sided) | Point Probability |
| Pearson Chi-Square | 16.999^a^ | 4 | .002 | .002 |  |  |
| Likelihood Ratio | 15.630 | 4 | .004 | .004 |  |  |
| Fisher's Exact Test | 15.755 |  |  | .003 |  |  |
| Linear-by-Linear Association | 13.086^b^ | 1 | .000 | .000 | .000 | .000 |
| N of Valid Cases | 409 |  |  |  |  |  |

| a. 1 cells (10.0%) have expected count less than 5. The minimum expected count is 3.25. |
| --- |
| b. The standardized statistic is -3.617. |

**3. I was satisfied with efforts the teacher put in * Age**

| **Crosstab** | | | | | |
| --- | --- | --- | --- | --- | --- |
|  | | | Age | | Total |
|  |  |  | 1 | 2 |  |
| 3. I was satisfied with efforts the teacher put in | 1 | Count | 6 | 4 | 10 |
|  |  | % within Age | 2.0% | 3.7% | 2.4% |
|  | 2 | Count | 13 | 0 | 13 |
|  |  | % within Age | 4.3% | 0.0% | 3.2% |
|  | 3 | Count | 25 | 9 | 34 |
|  |  | % within Age | 8.3% | 8.3% | 8.3% |
|  | 4 | Count | 136 | 67 | 203 |
|  |  | % within Age | 45.3% | 61.5% | 49.6% |
|  | 5 | Count | 120 | 29 | 149 |
|  |  | % within Age | 40.0% | 26.6% | 36.4% |
| Total | | Count | 300 | 109 | 409 |
|  |  | % within Age | 100.0% | 100.0% | 100.0% |

| **Chi-Square Tests** | | | | | | |
| --- | --- | --- | --- | --- | --- | --- |
|  | Value | df | Asymp. Sig. (2-sided) | Exact Sig. (2-sided) | Exact Sig. (1-sided) | Point Probability |
| Pearson Chi-Square | 13.766^a^ | 4 | .008 | .008 |  |  |
| Likelihood Ratio | 17.111 | 4 | .002 | .002 |  |  |
| Fisher's Exact Test | 14.524 |  |  | .004 |  |  |
| Linear-by-Linear Association | .962^b^ | 1 | .327 | .342 | .179 | .031 |
| N of Valid Cases | 409 |  |  |  |  |  |

| a. 2 cells (20.0%) have expected count less than 5. The minimum expected count is 2.67. |
| --- |
| b. The standardized statistic is -.981. |

**3. I was satisfied with efforts the teacher put in * Course of study**

| **Crosstab** | | | | | |
| --- | --- | --- | --- | --- | --- |
|  | | | Course of study | | Total |
|  |  |  | 1 | 2 |  |
| 3. I was satisfied with efforts the teacher put in | 1 | Count | 8 | 2 | 10 |
|  |  | % within Course of study | 2.5% | 2.3% | 2.4% |
|  | 2 | Count | 12 | 1 | 13 |
|  |  | % within Course of study | 3.7% | 1.2% | 3.2% |
|  | 3 | Count | 22 | 12 | 34 |
|  |  | % within Course of study | 6.8% | 14.0% | 8.3% |
|  | 4 | Count | 152 | 51 | 203 |
|  |  | % within Course of study | 47.1% | 59.3% | 49.6% |
|  | 5 | Count | 129 | 20 | 149 |
|  |  | % within Course of study | 39.9% | 23.3% | 36.4% |
| Total | | Count | 323 | 86 | 409 |
|  |  | % within Course of study | 100.0% | 100.0% | 100.0% |

| **Chi-Square Tests** | | | | | | |
| --- | --- | --- | --- | --- | --- | --- |
|  | Value | df | Asymp. Sig. (2-sided) | Exact Sig. (2-sided) | Exact Sig. (1-sided) | Point Probability |
| Pearson Chi-Square | 12.806^a^ | 4 | .012 | .013 |  |  |
| Likelihood Ratio | 13.129 | 4 | .011 | .013 |  |  |
| Fisher's Exact Test | 12.606 |  |  | .010 |  |  |
| Linear-by-Linear Association | 2.921^b^ | 1 | .087 | .098 | .053 | .013 |
| N of Valid Cases | 409 |  |  |  |  |  |

| a. 2 cells (20.0%) have expected count less than 5. The minimum expected count is 2.10. |
| --- |
| b. The standardized statistic is -1.709. |

**3. I was satisfied with efforts the teacher put in * Place**

| **Crosstab** | | | | | | |
| --- | --- | --- | --- | --- | --- | --- |
|  | | | Place | | | Total |
|  |  |  | 1 | 2 | 3 |  |
| 3. I was satisfied with efforts the teacher put in | 1 | Count | 1 | 1 | 8 | 10 |
|  |  | % within Place | 0.6% | 1.7% | 4.2% | 2.4% |
|  | 2 | Count | 2 | 5 | 6 | 13 |
|  |  | % within Place | 1.2% | 8.3% | 3.2% | 3.2% |
|  | 3 | Count | 13 | 10 | 11 | 34 |
|  |  | % within Place | 8.1% | 16.7% | 5.8% | 8.3% |
|  | 4 | Count | 86 | 32 | 85 | 203 |
|  |  | % within Place | 53.8% | 53.3% | 45.0% | 49.6% |
|  | 5 | Count | 58 | 12 | 79 | 149 |
|  |  | % within Place | 36.2% | 20.0% | 41.8% | 36.4% |
| Total | | Count | 160 | 60 | 189 | 409 |
|  |  | % within Place | 100.0% | 100.0% | 100.0% | 100.0% |

| **Chi-Square Tests** | | | | | | |
| --- | --- | --- | --- | --- | --- | --- |
|  | Value | df | Asymp. Sig. (2-sided) | Exact Sig. (2-sided) | Exact Sig. (1-sided) | Point Probability |
| Pearson Chi-Square | 25.613^a^ | 8 | .001 | .^b^ |  |  |
| Likelihood Ratio | 24.952 | 8 | .002 | .^b^ |  |  |
| Fisher's Exact Test | 24.206 |  |  | .001 |  |  |
| Linear-by-Linear Association | .385^c^ | 1 | .535 | .543 | .278 | .020 |
| N of Valid Cases | 409 |  |  |  |  |  |

| a. 5 cells (33.3%) have expected count less than 5. The minimum expected count is 1.47. |
| --- |
| b. Cannot be computed because there is insufficient memory. |
| c. The standardized statistic is -.621. |

**3. I was satisfied with efforts the teacher put in * E learning skills**

| **Crosstab** | | | | | |
| --- | --- | --- | --- | --- | --- |
|  | | | E learning skills | | Total |
|  |  |  | 1 | 2 |  |
| 3. I was satisfied with efforts the teacher put in | 1 | Count | 6 | 4 | 10 |
|  |  | % within E learning skills | 1.8% | 4.8% | 2.4% |
|  | 2 | Count | 6 | 7 | 13 |
|  |  | % within E learning skills | 1.8% | 8.4% | 3.2% |
|  | 3 | Count | 25 | 9 | 34 |
|  |  | % within E learning skills | 7.7% | 10.8% | 8.3% |
|  | 4 | Count | 154 | 49 | 203 |
|  |  | % within E learning skills | 47.2% | 59.0% | 49.6% |
|  | 5 | Count | 135 | 14 | 149 |
|  |  | % within E learning skills | 41.4% | 16.9% | 36.4% |
| Total | | Count | 326 | 83 | 409 |
|  |  | % within E learning skills | 100.0% | 100.0% | 100.0% |

| **Chi-Square Tests** | | | | | | |
| --- | --- | --- | --- | --- | --- | --- |
|  | Value | df | Asymp. Sig. (2-sided) | Exact Sig. (2-sided) | Exact Sig. (1-sided) | Point Probability |
| Pearson Chi-Square | 25.045^a^ | 4 | .000 | .000 |  |  |
| Likelihood Ratio | 24.691 | 4 | .000 | .000 |  |  |
| Fisher's Exact Test | 25.307 |  |  | .000 |  |  |
| Linear-by-Linear Association | 21.186^b^ | 1 | .000 | .000 | .000 | .000 |
| N of Valid Cases | 409 |  |  |  |  |  |

| a. 2 cells (20.0%) have expected count less than 5. The minimum expected count is 2.03. |
| --- |
| b. The standardized statistic is -4.603. |

**4. Online teaching helped me understand the subject better * Age**

| **Crosstab** | | | | | |
| --- | --- | --- | --- | --- | --- |
|  | | | Age | | Total |
|  |  |  | 1 | 2 |  |
| 4. Online teaching helped me understand the subject better | 1 | Count | 47 | 12 | 59 |
|  |  | % within Age | 15.7% | 11.0% | 14.4% |
|  | 2 | Count | 63 | 21 | 84 |
|  |  | % within Age | 21.0% | 19.3% | 20.5% |
|  | 3 | Count | 91 | 42 | 133 |
|  |  | % within Age | 30.3% | 38.5% | 32.5% |
|  | 4 | Count | 77 | 28 | 105 |
|  |  | % within Age | 25.7% | 25.7% | 25.7% |
|  | 5 | Count | 22 | 6 | 28 |
|  |  | % within Age | 7.3% | 5.5% | 6.8% |
| Total | | Count | 300 | 109 | 409 |
|  |  | % within Age | 100.0% | 100.0% | 100.0% |

| **Chi-Square Tests** | | | | | | |
| --- | --- | --- | --- | --- | --- | --- |
|  | Value | df | Asymp. Sig. (2-sided) | Exact Sig. (2-sided) | Exact Sig. (1-sided) | Point Probability |
| Pearson Chi-Square | 3.363^a^ | 4 | .499 | .502 |  |  |
| Likelihood Ratio | 3.395 | 4 | .494 | .502 |  |  |
| Fisher's Exact Test | 3.179 |  |  | .528 |  |  |
| Linear-by-Linear Association | .336^b^ | 1 | .562 | .591 | .298 | .033 |
| N of Valid Cases | 409 |  |  |  |  |  |

| a. 0 cells (0.0%) have expected count less than 5. The minimum expected count is 7.46. |
| --- |
| b. The standardized statistic is .580. |

**4. Online teaching helped me understand the subject better * Course of study**

| **Crosstab** | | | | | |
| --- | --- | --- | --- | --- | --- |
|  | | | Course of study | | Total |
|  |  |  | 1 | 2 |  |
| 4. Online teaching helped me understand the subject better | 1 | Count | 48 | 11 | 59 |
|  |  | % within Course of study | 14.9% | 12.8% | 14.4% |
|  | 2 | Count | 61 | 23 | 84 |
|  |  | % within Course of study | 18.9% | 26.7% | 20.5% |
|  | 3 | Count | 105 | 28 | 133 |
|  |  | % within Course of study | 32.5% | 32.6% | 32.5% |
|  | 4 | Count | 83 | 22 | 105 |
|  |  | % within Course of study | 25.7% | 25.6% | 25.7% |
|  | 5 | Count | 26 | 2 | 28 |
|  |  | % within Course of study | 8.0% | 2.3% | 6.8% |
| Total | | Count | 323 | 86 | 409 |
|  |  | % within Course of study | 100.0% | 100.0% | 100.0% |

| **Chi-Square Tests** | | | | | | |
| --- | --- | --- | --- | --- | --- | --- |
|  | Value | df | Asymp. Sig. (2-sided) | Exact Sig. (2-sided) | Exact Sig. (1-sided) | Point Probability |
| Pearson Chi-Square | 5.495^a^ | 4 | .240 | .241 |  |  |
| Likelihood Ratio | 6.224 | 4 | .183 | .190 |  |  |
| Fisher's Exact Test | 5.513 |  |  | .236 |  |  |
| Linear-by-Linear Association | 1.214^b^ | 1 | .270 | .289 | .147 | .023 |
| N of Valid Cases | 409 |  |  |  |  |  |

| a. 0 cells (0.0%) have expected count less than 5. The minimum expected count is 5.89. |
| --- |
| b. The standardized statistic is -1.102. |

**4. Online teaching helped me understand the subject better * Place**

| **Crosstab** | | | | | | |
| --- | --- | --- | --- | --- | --- | --- |
|  | | | Place | | | Total |
|  |  |  | 1 | 2 | 3 |  |
| 4. Online teaching helped me understand the subject better | 1 | Count | 18 | 10 | 31 | 59 |
|  |  | % within Place | 11.2% | 16.7% | 16.4% | 14.4% |
|  | 2 | Count | 35 | 19 | 30 | 84 |
|  |  | % within Place | 21.9% | 31.7% | 15.9% | 20.5% |
|  | 3 | Count | 54 | 15 | 64 | 133 |
|  |  | % within Place | 33.8% | 25.0% | 33.9% | 32.5% |
|  | 4 | Count | 45 | 12 | 48 | 105 |
|  |  | % within Place | 28.1% | 20.0% | 25.4% | 25.7% |
|  | 5 | Count | 8 | 4 | 16 | 28 |
|  |  | % within Place | 5.0% | 6.7% | 8.5% | 6.8% |
| Total | | Count | 160 | 60 | 189 | 409 |
|  |  | % within Place | 100.0% | 100.0% | 100.0% | 100.0% |

| **Chi-Square Tests** | | | | | |
| --- | --- | --- | --- | --- | --- |
|  | Value | df | Asymp. Sig. (2-sided) | Exact Sig. (2-sided) | Exact Sig. (1-sided) |
| Pearson Chi-Square | 11.478^a^ | 8 | .176 | .^b^ |  |
| Likelihood Ratio | 11.360 | 8 | .182 | .^b^ |  |
| Fisher's Exact Test | .^b^ |  |  | .^b^ |  |
| Linear-by-Linear Association | .002 | 1 | .966 | .^b^ | .^b^ |
| N of Valid Cases | 409 |  |  |  |  |

| a. 1 cells (6.7%) have expected count less than 5. The minimum expected count is 4.11. |
| --- |
| b. Cannot be computed because there is insufficient memory. |

**4. Online teaching helped me understand the subject better * E learning skills**

| **Crosstab** | | | | | |
| --- | --- | --- | --- | --- | --- |
|  | | | E learning skills | | Total |
|  |  |  | 1 | 2 |  |
| 4. Online teaching helped me understand the subject better | 1 | Count | 40 | 19 | 59 |
|  |  | % within E learning skills | 12.3% | 22.9% | 14.4% |
|  | 2 | Count | 68 | 16 | 84 |
|  |  | % within E learning skills | 20.9% | 19.3% | 20.5% |
|  | 3 | Count | 105 | 28 | 133 |
|  |  | % within E learning skills | 32.2% | 33.7% | 32.5% |
|  | 4 | Count | 87 | 18 | 105 |
|  |  | % within E learning skills | 26.7% | 21.7% | 25.7% |
|  | 5 | Count | 26 | 2 | 28 |
|  |  | % within E learning skills | 8.0% | 2.4% | 6.8% |
| Total | | Count | 326 | 83 | 409 |
|  |  | % within E learning skills | 100.0% | 100.0% | 100.0% |

| **Chi-Square Tests** | | | | | | |
| --- | --- | --- | --- | --- | --- | --- |
|  | Value | df | Asymp. Sig. (2-sided) | Exact Sig. (2-sided) | Exact Sig. (1-sided) | Point Probability |
| Pearson Chi-Square | 8.940^a^ | 4 | .063 | .062 |  |  |
| Likelihood Ratio | 9.165 | 4 | .057 | .061 |  |  |
| Fisher's Exact Test | 8.541 |  |  | .071 |  |  |
| Linear-by-Linear Association | 6.489^b^ | 1 | .011 | .011 | .006 | .002 |
| N of Valid Cases | 409 |  |  |  |  |  |

| a. 0 cells (0.0%) have expected count less than 5. The minimum expected count is 5.68. |
| --- |
| b. The standardized statistic is -2.547. |

**5. Overall, I was satisfied with the quality of learning I had from online teaching * Age**

| **Crosstab** | | | | | |
| --- | --- | --- | --- | --- | --- |
|  | | | Age | | Total |
|  |  |  | 1 | 2 |  |
| 5. Overall, I was satisfied with the quality of learning I had from online teaching | 1 | Count | 38 | 6 | 44 |
|  |  | % within Age | 12.7% | 5.5% | 10.8% |
|  | 2 | Count | 57 | 28 | 85 |
|  |  | % within Age | 19.0% | 25.7% | 20.8% |
|  | 3 | Count | 52 | 35 | 87 |
|  |  | % within Age | 17.3% | 32.1% | 21.3% |
|  | 4 | Count | 128 | 37 | 165 |
|  |  | % within Age | 42.7% | 33.9% | 40.3% |
|  | 5 | Count | 25 | 3 | 28 |
|  |  | % within Age | 8.3% | 2.8% | 6.8% |
| Total | | Count | 300 | 109 | 409 |
|  |  | % within Age | 100.0% | 100.0% | 100.0% |

| **Chi-Square Tests** | | | | | | |
| --- | --- | --- | --- | --- | --- | --- |
|  | Value | df | Asymp. Sig. (2-sided) | Exact Sig. (2-sided) | Exact Sig. (1-sided) | Point Probability |
| Pearson Chi-Square | 18.885^a^ | 4 | .001 | .001 |  |  |
| Likelihood Ratio | 19.479 | 4 | .001 | .001 |  |  |
| Fisher's Exact Test | 18.570 |  |  | .001 |  |  |
| Linear-by-Linear Association | .919^b^ | 1 | .338 | .353 | .182 | .025 |
| N of Valid Cases | 409 |  |  |  |  |  |

| a. 0 cells (0.0%) have expected count less than 5. The minimum expected count is 7.46. |
| --- |
| b. The standardized statistic is -.959. |

**5. Overall, I was satisfied with the quality of learning I had from online teaching * Course of study**

| **Crosstab** | | | | | |
| --- | --- | --- | --- | --- | --- |
|  | | | Course of study | | Total |
|  |  |  | 1 | 2 |  |
| 5. Overall, I was satisfied with the quality of learning I had from online teaching | 1 | Count | 38 | 6 | 44 |
|  |  | % within Course of study | 11.8% | 7.0% | 10.8% |
|  | 2 | Count | 61 | 24 | 85 |
|  |  | % within Course of study | 18.9% | 27.9% | 20.8% |
|  | 3 | Count | 64 | 23 | 87 |
|  |  | % within Course of study | 19.8% | 26.7% | 21.3% |
|  | 4 | Count | 135 | 30 | 165 |
|  |  | % within Course of study | 41.8% | 34.9% | 40.3% |
|  | 5 | Count | 25 | 3 | 28 |
|  |  | % within Course of study | 7.7% | 3.5% | 6.8% |
| Total | | Count | 323 | 86 | 409 |
|  |  | % within Course of study | 100.0% | 100.0% | 100.0% |

| **Chi-Square Tests** | | | | | | |
| --- | --- | --- | --- | --- | --- | --- |
|  | Value | df | Asymp. Sig. (2-sided) | Exact Sig. (2-sided) | Exact Sig. (1-sided) | Point Probability |
| Pearson Chi-Square | 8.238^a^ | 4 | .083 | .082 |  |  |
| Likelihood Ratio | 8.447 | 4 | .077 | .082 |  |  |
| Fisher's Exact Test | 7.903 |  |  | .092 |  |  |
| Linear-by-Linear Association | 1.149^b^ | 1 | .284 | .289 | .154 | .024 |
| N of Valid Cases | 409 |  |  |  |  |  |

| a. 0 cells (0.0%) have expected count less than 5. The minimum expected count is 5.89. |
| --- |
| b. The standardized statistic is -1.072. |

**5. Overall, I was satisfied with the quality of learning I had from online teaching * Place**

| **Crosstab** | | | | | | |
| --- | --- | --- | --- | --- | --- | --- |
|  | | | Place | | | Total |
|  |  |  | 1 | 2 | 3 |  |
| 5. Overall, I was satisfied with the quality of learning I had from online teaching | 1 | Count | 14 | 9 | 21 | 44 |
|  |  | % within Place | 8.8% | 15.0% | 11.1% | 10.8% |
|  | 2 | Count | 40 | 15 | 30 | 85 |
|  |  | % within Place | 25.0% | 25.0% | 15.9% | 20.8% |
|  | 3 | Count | 37 | 14 | 36 | 87 |
|  |  | % within Place | 23.1% | 23.3% | 19.0% | 21.3% |
|  | 4 | Count | 62 | 20 | 83 | 165 |
|  |  | % within Place | 38.8% | 33.3% | 43.9% | 40.3% |
|  | 5 | Count | 7 | 2 | 19 | 28 |
|  |  | % within Place | 4.4% | 3.3% | 10.1% | 6.8% |
| Total | | Count | 160 | 60 | 189 | 409 |
|  |  | % within Place | 100.0% | 100.0% | 100.0% | 100.0% |

| **Chi-Square Tests** | | | | | |
| --- | --- | --- | --- | --- | --- |
|  | Value | df | Asymp. Sig. (2-sided) | Exact Sig. (2-sided) | Exact Sig. (1-sided) |
| Pearson Chi-Square | 13.295^a^ | 8 | .102 | .^b^ |  |
| Likelihood Ratio | 13.446 | 8 | .097 | .^b^ |  |
| Fisher's Exact Test | .^b^ |  |  | .^b^ |  |
| Linear-by-Linear Association | 3.122 | 1 | .077 | .^b^ | .^b^ |
| N of Valid Cases | 409 |  |  |  |  |

| a. 1 cells (6.7%) have expected count less than 5. The minimum expected count is 4.11. |
| --- |
| b. Cannot be computed because there is insufficient memory. |

**5. Overall, I was satisfied with the quality of learning I had from online teaching * E learning skills**

| **Crosstab** | | | | | |
| --- | --- | --- | --- | --- | --- |
|  | | | E learning skills | | Total |
|  |  |  | 1 | 2 |  |
| 5. Overall, I was satisfied with the quality of learning I had from online teaching | 1 | Count | 31 | 13 | 44 |
|  |  | % within E learning skills | 9.5% | 15.7% | 10.8% |
|  | 2 | Count | 61 | 24 | 85 |
|  |  | % within E learning skills | 18.7% | 28.9% | 20.8% |
|  | 3 | Count | 65 | 22 | 87 |
|  |  | % within E learning skills | 19.9% | 26.5% | 21.3% |
|  | 4 | Count | 144 | 21 | 165 |
|  |  | % within E learning skills | 44.2% | 25.3% | 40.3% |
|  | 5 | Count | 25 | 3 | 28 |
|  |  | % within E learning skills | 7.7% | 3.6% | 6.8% |
| Total | | Count | 326 | 83 | 409 |
|  |  | % within E learning skills | 100.0% | 100.0% | 100.0% |

| **Chi-Square Tests** | | | | | | |
| --- | --- | --- | --- | --- | --- | --- |
|  | Value | df | Asymp. Sig. (2-sided) | Exact Sig. (2-sided) | Exact Sig. (1-sided) | Point Probability |
| Pearson Chi-Square | 14.412^a^ | 4 | .006 | .006 |  |  |
| Likelihood Ratio | 14.798 | 4 | .005 | .006 |  |  |
| Fisher's Exact Test | 14.623 |  |  | .005 |  |  |
| Linear-by-Linear Association | 12.418^b^ | 1 | .000 | .000 | .000 | .000 |
| N of Valid Cases | 409 |  |  |  |  |  |

| a. 0 cells (0.0%) have expected count less than 5. The minimum expected count is 5.68. |
| --- |
| b. The standardized statistic is -3.524. |

**6. It was possible to interact with the teacher privately in multiple ways during or after online teaching * Age**

| **Crosstab** | | | | | |
| --- | --- | --- | --- | --- | --- |
|  | | | Age | | Total |
|  |  |  | 1 | 2 |  |
| 6. It was possible to interact with the teacher privately in multiple ways during or after online teaching | 1 | Count | 20 | 4 | 24 |
|  |  | % within Age | 6.7% | 3.7% | 5.9% |
|  | 2 | Count | 56 | 14 | 70 |
|  |  | % within Age | 18.7% | 12.8% | 17.1% |
|  | 3 | Count | 82 | 34 | 116 |
|  |  | % within Age | 27.3% | 31.2% | 28.4% |
|  | 4 | Count | 113 | 49 | 162 |
|  |  | % within Age | 37.7% | 45.0% | 39.6% |
|  | 5 | Count | 29 | 8 | 37 |
|  |  | % within Age | 9.7% | 7.3% | 9.0% |
| Total | | Count | 300 | 109 | 409 |
|  |  | % within Age | 100.0% | 100.0% | 100.0% |

| **Chi-Square Tests** | | | | | | |
| --- | --- | --- | --- | --- | --- | --- |
|  | Value | df | Asymp. Sig. (2-sided) | Exact Sig. (2-sided) | Exact Sig. (1-sided) | Point Probability |
| Pearson Chi-Square | 4.778^a^ | 4 | .311 | .314 |  |  |
| Likelihood Ratio | 4.987 | 4 | .289 | .297 |  |  |
| Fisher's Exact Test | 4.522 |  |  | .339 |  |  |
| Linear-by-Linear Association | 1.541^b^ | 1 | .214 | .217 | .117 | .020 |
| N of Valid Cases | 409 |  |  |  |  |  |

| a. 0 cells (0.0%) have expected count less than 5. The minimum expected count is 6.40. |
| --- |
| b. The standardized statistic is 1.241. |

**6. It was possible to interact with the teacher privately in multiple ways during or after online teaching * Course of study**

| **Crosstab** | | | | | |
| --- | --- | --- | --- | --- | --- |
|  | | | Course of study | | Total |
|  |  |  | 1 | 2 |  |
| 6. It was possible to interact with the teacher privately in multiple ways during or after online teaching | 1 | Count | 19 | 5 | 24 |
|  |  | % within Course of study | 5.9% | 5.8% | 5.9% |
|  | 2 | Count | 53 | 17 | 70 |
|  |  | % within Course of study | 16.4% | 19.8% | 17.1% |
|  | 3 | Count | 87 | 29 | 116 |
|  |  | % within Course of study | 26.9% | 33.7% | 28.4% |
|  | 4 | Count | 133 | 29 | 162 |
|  |  | % within Course of study | 41.2% | 33.7% | 39.6% |
|  | 5 | Count | 31 | 6 | 37 |
|  |  | % within Course of study | 9.6% | 7.0% | 9.0% |
| Total | | Count | 323 | 86 | 409 |
|  |  | % within Course of study | 100.0% | 100.0% | 100.0% |

| **Chi-Square Tests** | | | | | | |
| --- | --- | --- | --- | --- | --- | --- |
|  | Value | df | Asymp. Sig. (2-sided) | Exact Sig. (2-sided) | Exact Sig. (1-sided) | Point Probability |
| Pearson Chi-Square | 3.020^a^ | 4 | .555 | .561 |  |  |
| Likelihood Ratio | 3.029 | 4 | .553 | .568 |  |  |
| Fisher's Exact Test | 3.007 |  |  | .557 |  |  |
| Linear-by-Linear Association | 1.589^b^ | 1 | .207 | .221 | .115 | .021 |
| N of Valid Cases | 409 |  |  |  |  |  |

| a. 0 cells (0.0%) have expected count less than 5. The minimum expected count is 5.05. |
| --- |
| b. The standardized statistic is -1.261. |

**6. It was possible to interact with the teacher privately in multiple ways during or after online teaching * Place**

| **Crosstab** | | | | | | |
| --- | --- | --- | --- | --- | --- | --- |
|  | | | Place | | | Total |
|  |  |  | 1 | 2 | 3 |  |
| 6. It was possible to interact with the teacher privately in multiple ways during or after online teaching | 1 | Count | 8 | 3 | 13 | 24 |
|  |  | % within Place | 5.0% | 5.0% | 6.9% | 5.9% |
|  | 2 | Count | 26 | 14 | 30 | 70 |
|  |  | % within Place | 16.2% | 23.3% | 15.9% | 17.1% |
|  | 3 | Count | 43 | 15 | 58 | 116 |
|  |  | % within Place | 26.9% | 25.0% | 30.7% | 28.4% |
|  | 4 | Count | 68 | 24 | 70 | 162 |
|  |  | % within Place | 42.5% | 40.0% | 37.0% | 39.6% |
|  | 5 | Count | 15 | 4 | 18 | 37 |
|  |  | % within Place | 9.4% | 6.7% | 9.5% | 9.0% |
| Total | | Count | 160 | 60 | 189 | 409 |
|  |  | % within Place | 100.0% | 100.0% | 100.0% | 100.0% |

| **Chi-Square Tests** | | | | | |
| --- | --- | --- | --- | --- | --- |
|  | Value | df | Asymp. Sig. (2-sided) | Exact Sig. (2-sided) | Exact Sig. (1-sided) |
| Pearson Chi-Square | 4.029^a^ | 8 | .854 | .859 |  |
| Likelihood Ratio | 3.932 | 8 | .863 | .871 |  |
| Fisher's Exact Test | 3.831 |  |  | .877 |  |
| Linear-by-Linear Association | .549 | 1 | .459 | .^b^ | .^b^ |
| N of Valid Cases | 409 |  |  |  |  |

| a. 1 cells (6.7%) have expected count less than 5. The minimum expected count is 3.52. |
| --- |
| b. Cannot be computed because there is insufficient memory. |

**6. It was possible to interact with the teacher privately in multiple ways during or after online teaching * E learning skills**

| **Crosstab** | | | | | |
| --- | --- | --- | --- | --- | --- |
|  | | | E learning skills | | Total |
|  |  |  | 1 | 2 |  |
| 6. It was possible to interact with the teacher privately in multiple ways during or after online teaching | 1 | Count | 16 | 8 | 24 |
|  |  | % within E learning skills | 4.9% | 9.6% | 5.9% |
|  | 2 | Count | 49 | 21 | 70 |
|  |  | % within E learning skills | 15.0% | 25.3% | 17.1% |
|  | 3 | Count | 90 | 26 | 116 |
|  |  | % within E learning skills | 27.6% | 31.3% | 28.4% |
|  | 4 | Count | 140 | 22 | 162 |
|  |  | % within E learning skills | 42.9% | 26.5% | 39.6% |
|  | 5 | Count | 31 | 6 | 37 |
|  |  | % within E learning skills | 9.5% | 7.2% | 9.0% |
| Total | | Count | 326 | 83 | 409 |
|  |  | % within E learning skills | 100.0% | 100.0% | 100.0% |

| **Chi-Square Tests** | | | | | | |
| --- | --- | --- | --- | --- | --- | --- |
|  | Value | df | Asymp. Sig. (2-sided) | Exact Sig. (2-sided) | Exact Sig. (1-sided) | Point Probability |
| Pearson Chi-Square | 11.817^a^ | 4 | .019 | .018 |  |  |
| Likelihood Ratio | 11.599 | 4 | .021 | .024 |  |  |
| Fisher's Exact Test | 11.830 |  |  | .017 |  |  |
| Linear-by-Linear Association | 10.132^b^ | 1 | .001 | .002 | .001 | .000 |
| N of Valid Cases | 409 |  |  |  |  |  |

| a. 1 cells (10.0%) have expected count less than 5. The minimum expected count is 4.87. |
| --- |
| b. The standardized statistic is -3.183. |

**7. It was possible for me to access online classes anytime and from anywhere * Age**

| **Crosstab** | | | | | |
| --- | --- | --- | --- | --- | --- |
|  | | | Age | | Total |
|  |  |  | 1 | 2 |  |
| 7. It was possible for me to access online classes anytime and from anywhere | 1 | Count | 32 | 14 | 46 |
|  |  | % within Age | 10.7% | 12.8% | 11.2% |
|  | 2 | Count | 48 | 32 | 80 |
|  |  | % within Age | 16.0% | 29.4% | 19.6% |
|  | 3 | Count | 55 | 11 | 66 |
|  |  | % within Age | 18.3% | 10.1% | 16.1% |
|  | 4 | Count | 107 | 41 | 148 |
|  |  | % within Age | 35.7% | 37.6% | 36.2% |
|  | 5 | Count | 58 | 11 | 69 |
|  |  | % within Age | 19.3% | 10.1% | 16.9% |
| Total | | Count | 300 | 109 | 409 |
|  |  | % within Age | 100.0% | 100.0% | 100.0% |

| **Chi-Square Tests** | | | | | | |
| --- | --- | --- | --- | --- | --- | --- |
|  | Value | df | Asymp. Sig. (2-sided) | Exact Sig. (2-sided) | Exact Sig. (1-sided) | Point Probability |
| Pearson Chi-Square | 15.127^a^ | 4 | .004 | .004 |  |  |
| Likelihood Ratio | 15.327 | 4 | .004 | .004 |  |  |
| Fisher's Exact Test | 14.971 |  |  | .005 |  |  |
| Linear-by-Linear Association | 5.830^b^ | 1 | .016 | .017 | .009 | .002 |
| N of Valid Cases | 409 |  |  |  |  |  |

| a. 0 cells (0.0%) have expected count less than 5. The minimum expected count is 12.26. |
| --- |
| b. The standardized statistic is -2.415. |

**7. It was possible for me to access online classes anytime and from anywhere * Course of study**

| **Crosstab** | | | | | |
| --- | --- | --- | --- | --- | --- |
|  | | | Course of study | | Total |
|  |  |  | 1 | 2 |  |
| 7. It was possible for me to access online classes anytime and from anywhere | 1 | Count | 32 | 14 | 46 |
|  |  | % within Course of study | 9.9% | 16.3% | 11.2% |
|  | 2 | Count | 56 | 24 | 80 |
|  |  | % within Course of study | 17.3% | 27.9% | 19.6% |
|  | 3 | Count | 53 | 13 | 66 |
|  |  | % within Course of study | 16.4% | 15.1% | 16.1% |
|  | 4 | Count | 122 | 26 | 148 |
|  |  | % within Course of study | 37.8% | 30.2% | 36.2% |
|  | 5 | Count | 60 | 9 | 69 |
|  |  | % within Course of study | 18.6% | 10.5% | 16.9% |
| Total | | Count | 323 | 86 | 409 |
|  |  | % within Course of study | 100.0% | 100.0% | 100.0% |

| **Chi-Square Tests** | | | | | | |
| --- | --- | --- | --- | --- | --- | --- |
|  | Value | df | Asymp. Sig. (2-sided) | Exact Sig. (2-sided) | Exact Sig. (1-sided) | Point Probability |
| Pearson Chi-Square | 10.116^a^ | 4 | .039 | .038 |  |  |
| Likelihood Ratio | 9.932 | 4 | .042 | .044 |  |  |
| Fisher's Exact Test | 9.902 |  |  | .041 |  |  |
| Linear-by-Linear Association | 9.357^b^ | 1 | .002 | .002 | .001 | .000 |
| N of Valid Cases | 409 |  |  |  |  |  |

| a. 0 cells (0.0%) have expected count less than 5. The minimum expected count is 9.67. |
| --- |
| b. The standardized statistic is -3.059. |

**7. It was possible for me to access online classes anytime and from anywhere * Place**

| **Crosstab** | | | | | | |
| --- | --- | --- | --- | --- | --- | --- |
|  | | | Place | | | Total |
|  |  |  | 1 | 2 | 3 |  |
| 7. It was possible for me to access online classes anytime and from anywhere | 1 | Count | 20 | 4 | 22 | 46 |
|  |  | % within Place | 12.5% | 6.7% | 11.6% | 11.2% |
|  | 2 | Count | 35 | 13 | 32 | 80 |
|  |  | % within Place | 21.9% | 21.7% | 16.9% | 19.6% |
|  | 3 | Count | 32 | 12 | 22 | 66 |
|  |  | % within Place | 20.0% | 20.0% | 11.6% | 16.1% |
|  | 4 | Count | 57 | 23 | 68 | 148 |
|  |  | % within Place | 35.6% | 38.3% | 36.0% | 36.2% |
|  | 5 | Count | 16 | 8 | 45 | 69 |
|  |  | % within Place | 10.0% | 13.3% | 23.8% | 16.9% |
| Total | | Count | 160 | 60 | 189 | 409 |
|  |  | % within Place | 100.0% | 100.0% | 100.0% | 100.0% |

| **Chi-Square Tests** | | | | | |
| --- | --- | --- | --- | --- | --- |
|  | Value | df | Asymp. Sig. (2-sided) | Exact Sig. (2-sided) | Exact Sig. (1-sided) |
| Pearson Chi-Square | 17.422^a^ | 8 | .026 | .^b^ |  |
| Likelihood Ratio | 17.920 | 8 | .022 | .^b^ |  |
| Fisher's Exact Test | .^b^ |  |  | .^b^ |  |
| Linear-by-Linear Association | 6.433 | 1 | .011 | .^b^ | .^b^ |
| N of Valid Cases | 409 |  |  |  |  |

| a. 0 cells (0.0%) have expected count less than 5. The minimum expected count is 6.75. |
| --- |
| b. Cannot be computed because there is insufficient memory. |

**7. It was possible for me to access online classes anytime and from anywhere * E learning skills**

| **Crosstab** | | | | | |
| --- | --- | --- | --- | --- | --- |
|  | | | E learning skills | | Total |
|  |  |  | 1 | 2 |  |
| 7. It was possible for me to access online classes anytime and from anywhere | 1 | Count | 28 | 18 | 46 |
|  |  | % within E learning skills | 8.6% | 21.7% | 11.2% |
|  | 2 | Count | 55 | 25 | 80 |
|  |  | % within E learning skills | 16.9% | 30.1% | 19.6% |
|  | 3 | Count | 52 | 14 | 66 |
|  |  | % within E learning skills | 16.0% | 16.9% | 16.1% |
|  | 4 | Count | 128 | 20 | 148 |
|  |  | % within E learning skills | 39.3% | 24.1% | 36.2% |
|  | 5 | Count | 63 | 6 | 69 |
|  |  | % within E learning skills | 19.3% | 7.2% | 16.9% |
| Total | | Count | 326 | 83 | 409 |
|  |  | % within E learning skills | 100.0% | 100.0% | 100.0% |

| **Chi-Square Tests** | | | | | | |
| --- | --- | --- | --- | --- | --- | --- |
|  | Value | df | Asymp. Sig. (2-sided) | Exact Sig. (2-sided) | Exact Sig. (1-sided) | Point Probability |
| Pearson Chi-Square | 26.006^a^ | 4 | .000 | .000 |  |  |
| Likelihood Ratio | 25.474 | 4 | .000 | .000 |  |  |
| Fisher's Exact Test | 25.208 |  |  | .000 |  |  |
| Linear-by-Linear Association | 25.544^b^ | 1 | .000 | .000 | .000 | .000 |
| N of Valid Cases | 409 |  |  |  |  |  |

| a. 0 cells (0.0%) have expected count less than 5. The minimum expected count is 9.33. |
| --- |
| b. The standardized statistic is -5.054. |

**8. I feel online teaching will be very useful in teaching theory subjects where there is no practical aspects (like languages, history, political science) * Age**

| **Crosstab** | | | | | |
| --- | --- | --- | --- | --- | --- |
|  | | | Age | | Total |
|  |  |  | 1 | 2 |  |
| 8. I feel online teaching will be very useful in teaching theory subjects where there is no practical aspects (like languages, history, political science) | 1 | Count | 28 | 7 | 35 |
|  |  | % within Age | 9.3% | 6.4% | 8.6% |
|  | 2 | Count | 52 | 23 | 75 |
|  |  | % within Age | 17.3% | 21.1% | 18.3% |
|  | 3 | Count | 59 | 18 | 77 |
|  |  | % within Age | 19.7% | 16.5% | 18.8% |
|  | 4 | Count | 117 | 43 | 160 |
|  |  | % within Age | 39.0% | 39.4% | 39.1% |
|  | 5 | Count | 44 | 18 | 62 |
|  |  | % within Age | 14.7% | 16.5% | 15.2% |
| Total | | Count | 300 | 109 | 409 |
|  |  | % within Age | 100.0% | 100.0% | 100.0% |

| **Chi-Square Tests** | | | | | | |
| --- | --- | --- | --- | --- | --- | --- |
|  | Value | df | Asymp. Sig. (2-sided) | Exact Sig. (2-sided) | Exact Sig. (1-sided) | Point Probability |
| Pearson Chi-Square | 2.017^a^ | 4 | .733 | .736 |  |  |
| Likelihood Ratio | 2.057 | 4 | .725 | .731 |  |  |
| Fisher's Exact Test | 1.967 |  |  | .746 |  |  |
| Linear-by-Linear Association | .218^b^ | 1 | .641 | .672 | .339 | .034 |
| N of Valid Cases | 409 |  |  |  |  |  |

| a. 0 cells (0.0%) have expected count less than 5. The minimum expected count is 9.33. |
| --- |
| b. The standardized statistic is .467. |

**8. I feel online teaching will be very useful in teaching theory subjects where there is no practical aspects (like languages, history, political science) * Course of study**

| **Crosstab** | | | | | |
| --- | --- | --- | --- | --- | --- |
|  | | | Course of study | | Total |
|  |  |  | 1 | 2 |  |
| 8. I feel online teaching will be very useful in teaching theory subjects where there is no practical aspects (like languages, history, political science) | 1 | Count | 26 | 9 | 35 |
|  |  | % within Course of study | 8.0% | 10.5% | 8.6% |
|  | 2 | Count | 56 | 19 | 75 |
|  |  | % within Course of study | 17.3% | 22.1% | 18.3% |
|  | 3 | Count | 61 | 16 | 77 |
|  |  | % within Course of study | 18.9% | 18.6% | 18.8% |
|  | 4 | Count | 132 | 28 | 160 |
|  |  | % within Course of study | 40.9% | 32.6% | 39.1% |
|  | 5 | Count | 48 | 14 | 62 |
|  |  | % within Course of study | 14.9% | 16.3% | 15.2% |
| Total | | Count | 323 | 86 | 409 |
|  |  | % within Course of study | 100.0% | 100.0% | 100.0% |

| **Chi-Square Tests** | | | | | | |
| --- | --- | --- | --- | --- | --- | --- |
|  | Value | df | Asymp. Sig. (2-sided) | Exact Sig. (2-sided) | Exact Sig. (1-sided) | Point Probability |
| Pearson Chi-Square | 2.592^a^ | 4 | .628 | .634 |  |  |
| Likelihood Ratio | 2.585 | 4 | .630 | .638 |  |  |
| Fisher's Exact Test | 2.815 |  |  | .591 |  |  |
| Linear-by-Linear Association | 1.091^b^ | 1 | .296 | .307 | .160 | .023 |
| N of Valid Cases | 409 |  |  |  |  |  |

| a. 0 cells (0.0%) have expected count less than 5. The minimum expected count is 7.36. |
| --- |
| b. The standardized statistic is -1.045. |

**8. I feel online teaching will be very useful in teaching theory subjects where there is no practical aspects (like languages, history, political science) * Place**

| **Crosstab** | | | | | | |
| --- | --- | --- | --- | --- | --- | --- |
|  | | | Place | | | Total |
|  |  |  | 1 | 2 | 3 |  |
| 8. I feel online teaching will be very useful in teaching theory subjects where there is no practical aspects (like languages, history, political science) | 1 | Count | 13 | 4 | 18 | 35 |
|  |  | % within Place | 8.1% | 6.7% | 9.5% | 8.6% |
|  | 2 | Count | 40 | 5 | 30 | 75 |
|  |  | % within Place | 25.0% | 8.3% | 15.9% | 18.3% |
|  | 3 | Count | 28 | 17 | 32 | 77 |
|  |  | % within Place | 17.5% | 28.3% | 16.9% | 18.8% |
|  | 4 | Count | 56 | 27 | 77 | 160 |
|  |  | % within Place | 35.0% | 45.0% | 40.7% | 39.1% |
|  | 5 | Count | 23 | 7 | 32 | 62 |
|  |  | % within Place | 14.4% | 11.7% | 16.9% | 15.2% |
| Total | | Count | 160 | 60 | 189 | 409 |
|  |  | % within Place | 100.0% | 100.0% | 100.0% | 100.0% |

| **Chi-Square Tests** | | | | | |
| --- | --- | --- | --- | --- | --- |
|  | Value | df | Asymp. Sig. (2-sided) | Exact Sig. (2-sided) | Exact Sig. (1-sided) |
| Pearson Chi-Square | 13.947^a^ | 8 | .083 | .^b^ |  |
| Likelihood Ratio | 14.090 | 8 | .079 | .^b^ |  |
| Fisher's Exact Test | .^b^ |  |  | .^b^ |  |
| Linear-by-Linear Association | 1.735 | 1 | .188 | .^b^ | .^b^ |
| N of Valid Cases | 409 |  |  |  |  |

| a. 0 cells (0.0%) have expected count less than 5. The minimum expected count is 5.13. |
| --- |
| b. Cannot be computed because there is insufficient memory. |

**8. I feel online teaching will be very useful in teaching theory subjects where there is no practical aspects (like languages, history, political science) * E learning skills**

| **Crosstab** | | | | | |
| --- | --- | --- | --- | --- | --- |
|  | | | E learning skills | | Total |
|  |  |  | 1 | 2 |  |
| 8. I feel online teaching will be very useful in teaching theory subjects where there is no practical aspects (like languages, history, political science) | 1 | Count | 24 | 11 | 35 |
|  |  | % within E learning skills | 7.4% | 13.3% | 8.6% |
|  | 2 | Count | 53 | 22 | 75 |
|  |  | % within E learning skills | 16.3% | 26.5% | 18.3% |
|  | 3 | Count | 66 | 11 | 77 |
|  |  | % within E learning skills | 20.2% | 13.3% | 18.8% |
|  | 4 | Count | 131 | 29 | 160 |
|  |  | % within E learning skills | 40.2% | 34.9% | 39.1% |
|  | 5 | Count | 52 | 10 | 62 |
|  |  | % within E learning skills | 16.0% | 12.0% | 15.2% |
| Total | | Count | 326 | 83 | 409 |
|  |  | % within E learning skills | 100.0% | 100.0% | 100.0% |

| **Chi-Square Tests** | | | | | | |
| --- | --- | --- | --- | --- | --- | --- |
|  | Value | df | Asymp. Sig. (2-sided) | Exact Sig. (2-sided) | Exact Sig. (1-sided) | Point Probability |
| Pearson Chi-Square | 9.320^a^ | 4 | .054 | .053 |  |  |
| Likelihood Ratio | 8.902 | 4 | .064 | .068 |  |  |
| Fisher's Exact Test | 8.956 |  |  | .060 |  |  |
| Linear-by-Linear Association | 5.769^b^ | 1 | .016 | .017 | .010 | .002 |
| N of Valid Cases | 409 |  |  |  |  |  |

| a. 0 cells (0.0%) have expected count less than 5. The minimum expected count is 7.10. |
| --- |
| b. The standardized statistic is -2.402. |

**9. I feel online teaching will be very useful in mass teaching (by one teacher) in the future. * Age**

| **Crosstab** | | | | | |
| --- | --- | --- | --- | --- | --- |
|  | | | Age | | Total |
|  |  |  | 1 | 2 |  |
| 9. I feel online teaching will be very useful in mass teaching (by one teacher) in the future. | 1 | Count | 40 | 11 | 51 |
|  |  | % within Age | 13.3% | 10.1% | 12.5% |
|  | 2 | Count | 56 | 27 | 83 |
|  |  | % within Age | 18.7% | 24.8% | 20.3% |
|  | 3 | Count | 65 | 24 | 89 |
|  |  | % within Age | 21.7% | 22.0% | 21.8% |
|  | 4 | Count | 100 | 38 | 138 |
|  |  | % within Age | 33.3% | 34.9% | 33.7% |
|  | 5 | Count | 39 | 9 | 48 |
|  |  | % within Age | 13.0% | 8.3% | 11.7% |
| Total | | Count | 300 | 109 | 409 |
|  |  | % within Age | 100.0% | 100.0% | 100.0% |

| **Chi-Square Tests** | | | | | | |
| --- | --- | --- | --- | --- | --- | --- |
|  | Value | df | Asymp. Sig. (2-sided) | Exact Sig. (2-sided) | Exact Sig. (1-sided) | Point Probability |
| Pearson Chi-Square | 3.734^a^ | 4 | .443 | .446 |  |  |
| Likelihood Ratio | 3.821 | 4 | .431 | .438 |  |  |
| Fisher's Exact Test | 3.625 |  |  | .460 |  |  |
| Linear-by-Linear Association | .307^b^ | 1 | .580 | .584 | .305 | .031 |
| N of Valid Cases | 409 |  |  |  |  |  |

| a. 0 cells (0.0%) have expected count less than 5. The minimum expected count is 12.79. |
| --- |
| b. The standardized statistic is -.554. |

**9. I feel online teaching will be very useful in mass teaching (by one teacher) in the future. * Course of study**

| **Crosstab** | | | | | |
| --- | --- | --- | --- | --- | --- |
|  | | | Course of study | | Total |
|  |  |  | 1 | 2 |  |
| 9. I feel online teaching will be very useful in mass teaching (by one teacher) in the future. | 1 | Count | 39 | 12 | 51 |
|  |  | % within Course of study | 12.1% | 14.0% | 12.5% |
|  | 2 | Count | 59 | 24 | 83 |
|  |  | % within Course of study | 18.3% | 27.9% | 20.3% |
|  | 3 | Count | 72 | 17 | 89 |
|  |  | % within Course of study | 22.3% | 19.8% | 21.8% |
|  | 4 | Count | 114 | 24 | 138 |
|  |  | % within Course of study | 35.3% | 27.9% | 33.7% |
|  | 5 | Count | 39 | 9 | 48 |
|  |  | % within Course of study | 12.1% | 10.5% | 11.7% |
| Total | | Count | 323 | 86 | 409 |
|  |  | % within Course of study | 100.0% | 100.0% | 100.0% |

| **Chi-Square Tests** | | | | | | |
| --- | --- | --- | --- | --- | --- | --- |
|  | Value | df | Asymp. Sig. (2-sided) | Exact Sig. (2-sided) | Exact Sig. (1-sided) | Point Probability |
| Pearson Chi-Square | 4.750^a^ | 4 | .314 | .315 |  |  |
| Likelihood Ratio | 4.568 | 4 | .335 | .343 |  |  |
| Fisher's Exact Test | 4.616 |  |  | .327 |  |  |
| Linear-by-Linear Association | 2.613^b^ | 1 | .106 | .113 | .059 | .011 |
| N of Valid Cases | 409 |  |  |  |  |  |

| a. 0 cells (0.0%) have expected count less than 5. The minimum expected count is 10.09. |
| --- |
| b. The standardized statistic is -1.616. |

**9. I feel online teaching will be very useful in mass teaching (by one teacher) in the future. * Place**

| **Crosstab** | | | | | | |
| --- | --- | --- | --- | --- | --- | --- |
|  | | | Place | | | Total |
|  |  |  | 1 | 2 | 3 |  |
| 9. I feel online teaching will be very useful in mass teaching (by one teacher) in the future. | 1 | Count | 14 | 8 | 29 | 51 |
|  |  | % within Place | 8.8% | 13.3% | 15.3% | 12.5% |
|  | 2 | Count | 44 | 8 | 31 | 83 |
|  |  | % within Place | 27.5% | 13.3% | 16.4% | 20.3% |
|  | 3 | Count | 32 | 16 | 41 | 89 |
|  |  | % within Place | 20.0% | 26.7% | 21.7% | 21.8% |
|  | 4 | Count | 53 | 24 | 61 | 138 |
|  |  | % within Place | 33.1% | 40.0% | 32.3% | 33.7% |
|  | 5 | Count | 17 | 4 | 27 | 48 |
|  |  | % within Place | 10.6% | 6.7% | 14.3% | 11.7% |
| Total | | Count | 160 | 60 | 189 | 409 |
|  |  | % within Place | 100.0% | 100.0% | 100.0% | 100.0% |

| **Chi-Square Tests** | | | | | |
| --- | --- | --- | --- | --- | --- |
|  | Value | df | Asymp. Sig. (2-sided) | Exact Sig. (2-sided) | Exact Sig. (1-sided) |
| Pearson Chi-Square | 14.257^a^ | 8 | .075 | .^b^ |  |
| Likelihood Ratio | 14.370 | 8 | .073 | .^b^ |  |
| Fisher's Exact Test | .^b^ |  |  | .^b^ |  |
| Linear-by-Linear Association | .109 | 1 | .741 | .^b^ | .^b^ |
| N of Valid Cases | 409 |  |  |  |  |

| a. 0 cells (0.0%) have expected count less than 5. The minimum expected count is 7.04. |
| --- |
| b. Cannot be computed because there is insufficient memory. |

**9. I feel online teaching will be very useful in mass teaching (by one teacher) in the future. * E learning skills**

| **Crosstab** | | | | | |
| --- | --- | --- | --- | --- | --- |
|  | | | E learning skills | | Total |
|  |  |  | 1 | 2 |  |
| 9. I feel online teaching will be very useful in mass teaching (by one teacher) in the future. | 1 | Count | 34 | 17 | 51 |
|  |  | % within E learning skills | 10.4% | 20.5% | 12.5% |
|  | 2 | Count | 65 | 18 | 83 |
|  |  | % within E learning skills | 19.9% | 21.7% | 20.3% |
|  | 3 | Count | 73 | 16 | 89 |
|  |  | % within E learning skills | 22.4% | 19.3% | 21.8% |
|  | 4 | Count | 114 | 24 | 138 |
|  |  | % within E learning skills | 35.0% | 28.9% | 33.7% |
|  | 5 | Count | 40 | 8 | 48 |
|  |  | % within E learning skills | 12.3% | 9.6% | 11.7% |
| Total | | Count | 326 | 83 | 409 |
|  |  | % within E learning skills | 100.0% | 100.0% | 100.0% |

| **Chi-Square Tests** | | | | | | |
| --- | --- | --- | --- | --- | --- | --- |
|  | Value | df | Asymp. Sig. (2-sided) | Exact Sig. (2-sided) | Exact Sig. (1-sided) | Point Probability |
| Pearson Chi-Square | 6.865^a^ | 4 | .143 | .143 |  |  |
| Likelihood Ratio | 6.283 | 4 | .179 | .186 |  |  |
| Fisher's Exact Test | 6.344 |  |  | .172 |  |  |
| Linear-by-Linear Association | 4.859^b^ | 1 | .027 | .031 | .016 | .004 |
| N of Valid Cases | 409 |  |  |  |  |  |

| a. 0 cells (0.0%) have expected count less than 5. The minimum expected count is 9.74. |
| --- |
| b. The standardized statistic is -2.204. |

**1. I had problems with the internet connection and speed * Age**

| **Crosstab** | | | | | |
| --- | --- | --- | --- | --- | --- |
|  | | | Age | | Total |
|  |  |  | 1 | 2 |  |
| 1. I had problems with the internet connection and speed | 1 | Count | 14 | 0 | 14 |
|  |  | % within Age | 4.7% | 0.0% | 3.4% |
|  | 2 | Count | 46 | 16 | 62 |
|  |  | % within Age | 15.3% | 14.7% | 15.2% |
|  | 3 | Count | 43 | 10 | 53 |
|  |  | % within Age | 14.3% | 9.2% | 13.0% |
|  | 4 | Count | 133 | 56 | 189 |
|  |  | % within Age | 44.3% | 51.4% | 46.2% |
|  | 5 | Count | 64 | 27 | 91 |
|  |  | % within Age | 21.3% | 24.8% | 22.2% |
| Total | | Count | 300 | 109 | 409 |
|  |  | % within Age | 100.0% | 100.0% | 100.0% |

| **Chi-Square Tests** | | | | | | |
| --- | --- | --- | --- | --- | --- | --- |
|  | Value | df | Asymp. Sig. (2-sided) | Exact Sig. (2-sided) | Exact Sig. (1-sided) | Point Probability |
| Pearson Chi-Square | 8.034^a^ | 4 | .090 | .089 |  |  |
| Likelihood Ratio | 11.721 | 4 | .020 | .023 |  |  |
| Fisher's Exact Test | 8.778 |  |  | .064 |  |  |
| Linear-by-Linear Association | 3.900^b^ | 1 | .048 | .049 | .026 | .006 |
| N of Valid Cases | 409 |  |  |  |  |  |

| a. 1 cells (10.0%) have expected count less than 5. The minimum expected count is 3.73. |
| --- |
| b. The standardized statistic is 1.975. |

**1. I had problems with the internet connection and speed * Course of study**

| **Crosstab** | | | | | |
| --- | --- | --- | --- | --- | --- |
|  | | | Course of study | | Total |
|  |  |  | 1 | 2 |  |
| 1. I had problems with the internet connection and speed | 1 | Count | 13 | 1 | 14 |
|  |  | % within Course of study | 4.0% | 1.2% | 3.4% |
|  | 2 | Count | 48 | 14 | 62 |
|  |  | % within Course of study | 14.9% | 16.3% | 15.2% |
|  | 3 | Count | 45 | 8 | 53 |
|  |  | % within Course of study | 13.9% | 9.3% | 13.0% |
|  | 4 | Count | 143 | 46 | 189 |
|  |  | % within Course of study | 44.3% | 53.5% | 46.2% |
|  | 5 | Count | 74 | 17 | 91 |
|  |  | % within Course of study | 22.9% | 19.8% | 22.2% |
| Total | | Count | 323 | 86 | 409 |
|  |  | % within Course of study | 100.0% | 100.0% | 100.0% |

| **Chi-Square Tests** | | | | | | |
| --- | --- | --- | --- | --- | --- | --- |
|  | Value | df | Asymp. Sig. (2-sided) | Exact Sig. (2-sided) | Exact Sig. (1-sided) | Point Probability |
| Pearson Chi-Square | 4.388^a^ | 4 | .356 | .359 |  |  |
| Likelihood Ratio | 4.870 | 4 | .301 | .315 |  |  |
| Fisher's Exact Test | 3.968 |  |  | .409 |  |  |
| Linear-by-Linear Association | .304^b^ | 1 | .582 | .615 | .313 | .039 |
| N of Valid Cases | 409 |  |  |  |  |  |

| a. 1 cells (10.0%) have expected count less than 5. The minimum expected count is 2.94. |
| --- |
| b. The standardized statistic is .551. |

**1. I had problems with the internet connection and speed * Place**

| **Crosstab** | | | | | | |
| --- | --- | --- | --- | --- | --- | --- |
|  | | | Place | | | Total |
|  |  |  | 1 | 2 | 3 |  |
| 1. I had problems with the internet connection and speed | 1 | Count | 5 | 0 | 9 | 14 |
|  |  | % within Place | 3.1% | 0.0% | 4.8% | 3.4% |
|  | 2 | Count | 20 | 8 | 34 | 62 |
|  |  | % within Place | 12.5% | 13.3% | 18.0% | 15.2% |
|  | 3 | Count | 23 | 13 | 17 | 53 |
|  |  | % within Place | 14.4% | 21.7% | 9.0% | 13.0% |
|  | 4 | Count | 70 | 27 | 92 | 189 |
|  |  | % within Place | 43.8% | 45.0% | 48.7% | 46.2% |
|  | 5 | Count | 42 | 12 | 37 | 91 |
|  |  | % within Place | 26.2% | 20.0% | 19.6% | 22.2% |
| Total | | Count | 160 | 60 | 189 | 409 |
|  |  | % within Place | 100.0% | 100.0% | 100.0% | 100.0% |

| **Chi-Square Tests** | | | | | |
| --- | --- | --- | --- | --- | --- |
|  | Value | df | Asymp. Sig. (2-sided) | Exact Sig. (2-sided) | Exact Sig. (1-sided) |
| Pearson Chi-Square | 13.384^a^ | 8 | .099 | .^b^ |  |
| Likelihood Ratio | 14.967 | 8 | .060 | .^b^ |  |
| Fisher's Exact Test | .^b^ |  |  | .^b^ |  |
| Linear-by-Linear Association | 2.203 | 1 | .138 | .^b^ | .^b^ |
| N of Valid Cases | 409 |  |  |  |  |

| a. 1 cells (6.7%) have expected count less than 5. The minimum expected count is 2.05. |
| --- |
| b. Cannot be computed because there is insufficient memory. |

**1. I had problems with the internet connection and speed * E learning skills**

| **Crosstab** | | | | | |
| --- | --- | --- | --- | --- | --- |
|  | | | E learning skills | | Total |
|  |  |  | 1 | 2 |  |
| 1. I had problems with the internet connection and speed | 1 | Count | 10 | 4 | 14 |
|  |  | % within E learning skills | 3.1% | 4.8% | 3.4% |
|  | 2 | Count | 52 | 10 | 62 |
|  |  | % within E learning skills | 16.0% | 12.0% | 15.2% |
|  | 3 | Count | 46 | 7 | 53 |
|  |  | % within E learning skills | 14.1% | 8.4% | 13.0% |
|  | 4 | Count | 157 | 32 | 189 |
|  |  | % within E learning skills | 48.2% | 38.6% | 46.2% |
|  | 5 | Count | 61 | 30 | 91 |
|  |  | % within E learning skills | 18.7% | 36.1% | 22.2% |
| Total | | Count | 326 | 83 | 409 |
|  |  | % within E learning skills | 100.0% | 100.0% | 100.0% |

| **Chi-Square Tests** | | | | | | |
| --- | --- | --- | --- | --- | --- | --- |
|  | Value | df | Asymp. Sig. (2-sided) | Exact Sig. (2-sided) | Exact Sig. (1-sided) | Point Probability |
| Pearson Chi-Square | 13.260^a^ | 4 | .010 | .010 |  |  |
| Likelihood Ratio | 12.436 | 4 | .014 | .017 |  |  |
| Fisher's Exact Test | 12.415 |  |  | .012 |  |  |
| Linear-by-Linear Association | 3.718^b^ | 1 | .054 | .061 | .029 | .007 |
| N of Valid Cases | 409 |  |  |  |  |  |

| a. 1 cells (10.0%) have expected count less than 5. The minimum expected count is 2.84. |
| --- |
| b. The standardized statistic is 1.928. |

**2. I used to have problems with the video streaming due to slow internet * Age**

| **Crosstab** | | | | | |
| --- | --- | --- | --- | --- | --- |
|  | | | Age | | Total |
|  |  |  | 1 | 2 |  |
| 2. I used to have problems with the video streaming due to slow internet | 1 | Count | 13 | 0 | 13 |
|  |  | % within Age | 4.3% | 0.0% | 3.2% |
|  | 2 | Count | 50 | 25 | 75 |
|  |  | % within Age | 16.7% | 22.9% | 18.3% |
|  | 3 | Count | 38 | 4 | 42 |
|  |  | % within Age | 12.7% | 3.7% | 10.3% |
|  | 4 | Count | 143 | 59 | 202 |
|  |  | % within Age | 47.7% | 54.1% | 49.4% |
|  | 5 | Count | 56 | 21 | 77 |
|  |  | % within Age | 18.7% | 19.3% | 18.8% |
| Total | | Count | 300 | 109 | 409 |
|  |  | % within Age | 100.0% | 100.0% | 100.0% |

| **Chi-Square Tests** | | | | | | |
| --- | --- | --- | --- | --- | --- | --- |
|  | Value | df | Asymp. Sig. (2-sided) | Exact Sig. (2-sided) | Exact Sig. (1-sided) | Point Probability |
| Pearson Chi-Square | 13.430^a^ | 4 | .009 | .009 |  |  |
| Likelihood Ratio | 18.087 | 4 | .001 | .001 |  |  |
| Fisher's Exact Test | 14.757 |  |  | .005 |  |  |
| Linear-by-Linear Association | .690^b^ | 1 | .406 | .410 | .219 | .030 |
| N of Valid Cases | 409 |  |  |  |  |  |

| a. 1 cells (10.0%) have expected count less than 5. The minimum expected count is 3.46. |
| --- |
| b. The standardized statistic is .831. |

**2. I used to have problems with the video streaming due to slow internet * Course of study**

| **Crosstab** | | | | | |
| --- | --- | --- | --- | --- | --- |
|  | | | Course of study | | Total |
|  |  |  | 1 | 2 |  |
| 2. I used to have problems with the video streaming due to slow internet | 1 | Count | 13 | 0 | 13 |
|  |  | % within Course of study | 4.0% | 0.0% | 3.2% |
|  | 2 | Count | 58 | 17 | 75 |
|  |  | % within Course of study | 18.0% | 19.8% | 18.3% |
|  | 3 | Count | 37 | 5 | 42 |
|  |  | % within Course of study | 11.5% | 5.8% | 10.3% |
|  | 4 | Count | 155 | 47 | 202 |
|  |  | % within Course of study | 48.0% | 54.7% | 49.4% |
|  | 5 | Count | 60 | 17 | 77 |
|  |  | % within Course of study | 18.6% | 19.8% | 18.8% |
| Total | | Count | 323 | 86 | 409 |
|  |  | % within Course of study | 100.0% | 100.0% | 100.0% |

| **Chi-Square Tests** | | | | | | |
| --- | --- | --- | --- | --- | --- | --- |
|  | Value | df | Asymp. Sig. (2-sided) | Exact Sig. (2-sided) | Exact Sig. (1-sided) | Point Probability |
| Pearson Chi-Square | 6.349^a^ | 4 | .175 | .172 |  |  |
| Likelihood Ratio | 9.303 | 4 | .054 | .064 |  |  |
| Fisher's Exact Test | 6.455 |  |  | .160 |  |  |
| Linear-by-Linear Association | 1.354^b^ | 1 | .245 | .263 | .134 | .023 |
| N of Valid Cases | 409 |  |  |  |  |  |

| a. 1 cells (10.0%) have expected count less than 5. The minimum expected count is 2.73. |
| --- |
| b. The standardized statistic is 1.164. |

**2. I used to have problems with the video streaming due to slow internet * Place**

| **Crosstab** | | | | | | |
| --- | --- | --- | --- | --- | --- | --- |
|  | | | Place | | | Total |
|  |  |  | 1 | 2 | 3 |  |
| 2. I used to have problems with the video streaming due to slow internet | 1 | Count | 3 | 1 | 9 | 13 |
|  |  | % within Place | 1.9% | 1.7% | 4.8% | 3.2% |
|  | 2 | Count | 28 | 9 | 38 | 75 |
|  |  | % within Place | 17.5% | 15.0% | 20.1% | 18.3% |
|  | 3 | Count | 18 | 7 | 17 | 42 |
|  |  | % within Place | 11.2% | 11.7% | 9.0% | 10.3% |
|  | 4 | Count | 79 | 32 | 91 | 202 |
|  |  | % within Place | 49.4% | 53.3% | 48.1% | 49.4% |
|  | 5 | Count | 32 | 11 | 34 | 77 |
|  |  | % within Place | 20.0% | 18.3% | 18.0% | 18.8% |
| Total | | Count | 160 | 60 | 189 | 409 |
|  |  | % within Place | 100.0% | 100.0% | 100.0% | 100.0% |

| **Chi-Square Tests** | | | | | | |
| --- | --- | --- | --- | --- | --- | --- |
|  | Value | df | Asymp. Sig. (2-sided) | Exact Sig. (2-sided) | Exact Sig. (1-sided) | Point Probability |
| Pearson Chi-Square | 4.532^a^ | 8 | .806 | .813 |  |  |
| Likelihood Ratio | 4.586 | 8 | .801 | .816 |  |  |
| Fisher's Exact Test | 4.103 |  |  | .854 |  |  |
| Linear-by-Linear Association | 1.427^b^ | 1 | .232 | .234 | .121 | .010 |
| N of Valid Cases | 409 |  |  |  |  |  |

| a. 1 cells (6.7%) have expected count less than 5. The minimum expected count is 1.91. |
| --- |
| b. The standardized statistic is -1.194. |

**2. I used to have problems with the video streaming due to slow internet * E learning skills**

| **Crosstab** | | | | | |
| --- | --- | --- | --- | --- | --- |
|  | | | E learning skills | | Total |
|  |  |  | 1 | 2 |  |
| 2. I used to have problems with the video streaming due to slow internet | 1 | Count | 10 | 3 | 13 |
|  |  | % within E learning skills | 3.1% | 3.6% | 3.2% |
|  | 2 | Count | 67 | 8 | 75 |
|  |  | % within E learning skills | 20.6% | 9.6% | 18.3% |
|  | 3 | Count | 34 | 8 | 42 |
|  |  | % within E learning skills | 10.4% | 9.6% | 10.3% |
|  | 4 | Count | 161 | 41 | 202 |
|  |  | % within E learning skills | 49.4% | 49.4% | 49.4% |
|  | 5 | Count | 54 | 23 | 77 |
|  |  | % within E learning skills | 16.6% | 27.7% | 18.8% |
| Total | | Count | 326 | 83 | 409 |
|  |  | % within E learning skills | 100.0% | 100.0% | 100.0% |

| **Chi-Square Tests** | | | | | | |
| --- | --- | --- | --- | --- | --- | --- |
|  | Value | df | Asymp. Sig. (2-sided) | Exact Sig. (2-sided) | Exact Sig. (1-sided) | Point Probability |
| Pearson Chi-Square | 8.766^a^ | 4 | .067 | .065 |  |  |
| Likelihood Ratio | 9.047 | 4 | .060 | .070 |  |  |
| Fisher's Exact Test | 8.934 |  |  | .057 |  |  |
| Linear-by-Linear Association | 5.827^b^ | 1 | .016 | .017 | .008 | .002 |
| N of Valid Cases | 409 |  |  |  |  |  |

| a. 1 cells (10.0%) have expected count less than 5. The minimum expected count is 2.64. |
| --- |
| b. The standardized statistic is 2.414. |

**3. I had problems in interacting with the teacher * Age**

| **Crosstab** | | | | | |
| --- | --- | --- | --- | --- | --- |
|  | | | Age | | Total |
|  |  |  | 1 | 2 |  |
| 3. I had problems in interacting with the teacher | 1 | Count | 21 | 3 | 24 |
|  |  | % within Age | 7.0% | 2.8% | 5.9% |
|  | 2 | Count | 97 | 31 | 128 |
|  |  | % within Age | 32.3% | 28.4% | 31.3% |
|  | 3 | Count | 66 | 25 | 91 |
|  |  | % within Age | 22.0% | 22.9% | 22.2% |
|  | 4 | Count | 92 | 41 | 133 |
|  |  | % within Age | 30.7% | 37.6% | 32.5% |
|  | 5 | Count | 24 | 9 | 33 |
|  |  | % within Age | 8.0% | 8.3% | 8.1% |
| Total | | Count | 300 | 109 | 409 |
|  |  | % within Age | 100.0% | 100.0% | 100.0% |

| **Chi-Square Tests** | | | | | | |
| --- | --- | --- | --- | --- | --- | --- |
|  | Value | df | Asymp. Sig. (2-sided) | Exact Sig. (2-sided) | Exact Sig. (1-sided) | Point Probability |
| Pearson Chi-Square | 4.070^a^ | 4 | .397 | .400 |  |  |
| Likelihood Ratio | 4.450 | 4 | .348 | .358 |  |  |
| Fisher's Exact Test | 4.020 |  |  | .403 |  |  |
| Linear-by-Linear Association | 2.635^b^ | 1 | .105 | .113 | .058 | .011 |
| N of Valid Cases | 409 |  |  |  |  |  |

| a. 0 cells (0.0%) have expected count less than 5. The minimum expected count is 6.40. |
| --- |
| b. The standardized statistic is 1.623. |

**3. I had problems in interacting with the teacher * Course of study**

| **Crosstab** | | | | | |
| --- | --- | --- | --- | --- | --- |
|  | | | Course of study | | Total |
|  |  |  | 1 | 2 |  |
| 3. I had problems in interacting with the teacher | 1 | Count | 20 | 4 | 24 |
|  |  | % within Course of study | 6.2% | 4.7% | 5.9% |
|  | 2 | Count | 104 | 24 | 128 |
|  |  | % within Course of study | 32.2% | 27.9% | 31.3% |
|  | 3 | Count | 76 | 15 | 91 |
|  |  | % within Course of study | 23.5% | 17.4% | 22.2% |
|  | 4 | Count | 97 | 36 | 133 |
|  |  | % within Course of study | 30.0% | 41.9% | 32.5% |
|  | 5 | Count | 26 | 7 | 33 |
|  |  | % within Course of study | 8.0% | 8.1% | 8.1% |
| Total | | Count | 323 | 86 | 409 |
|  |  | % within Course of study | 100.0% | 100.0% | 100.0% |

| **Chi-Square Tests** | | | | | | |
| --- | --- | --- | --- | --- | --- | --- |
|  | Value | df | Asymp. Sig. (2-sided) | Exact Sig. (2-sided) | Exact Sig. (1-sided) | Point Probability |
| Pearson Chi-Square | 4.729^a^ | 4 | .316 | .318 |  |  |
| Likelihood Ratio | 4.646 | 4 | .326 | .334 |  |  |
| Fisher's Exact Test | 4.463 |  |  | .342 |  |  |
| Linear-by-Linear Association | 2.134^b^ | 1 | .144 | .149 | .080 | .015 |
| N of Valid Cases | 409 |  |  |  |  |  |

| a. 0 cells (0.0%) have expected count less than 5. The minimum expected count is 5.05. |
| --- |
| b. The standardized statistic is 1.461. |

**3. I had problems in interacting with the teacher * Place**

| **Crosstab** | | | | | | |
| --- | --- | --- | --- | --- | --- | --- |
|  | | | Place | | | Total |
|  |  |  | 1 | 2 | 3 |  |
| 3. I had problems in interacting with the teacher | 1 | Count | 5 | 1 | 18 | 24 |
|  |  | % within Place | 3.1% | 1.7% | 9.5% | 5.9% |
|  | 2 | Count | 42 | 20 | 66 | 128 |
|  |  | % within Place | 26.2% | 33.3% | 34.9% | 31.3% |
|  | 3 | Count | 42 | 14 | 35 | 91 |
|  |  | % within Place | 26.2% | 23.3% | 18.5% | 22.2% |
|  | 4 | Count | 61 | 21 | 51 | 133 |
|  |  | % within Place | 38.1% | 35.0% | 27.0% | 32.5% |
|  | 5 | Count | 10 | 4 | 19 | 33 |
|  |  | % within Place | 6.2% | 6.7% | 10.1% | 8.1% |
| Total | | Count | 160 | 60 | 189 | 409 |
|  |  | % within Place | 100.0% | 100.0% | 100.0% | 100.0% |

| **Chi-Square Tests** | | | | | |
| --- | --- | --- | --- | --- | --- |
|  | Value | df | Asymp. Sig. (2-sided) | Exact Sig. (2-sided) | Exact Sig. (1-sided) |
| Pearson Chi-Square | 17.866^a^ | 8 | .022 | .^b^ |  |
| Likelihood Ratio | 18.398 | 8 | .018 | .^b^ |  |
| Fisher's Exact Test | .^b^ |  |  | .^b^ |  |
| Linear-by-Linear Association | 4.590 | 1 | .032 | .^b^ | .^b^ |
| N of Valid Cases | 409 |  |  |  |  |

| a. 2 cells (13.3%) have expected count less than 5. The minimum expected count is 3.52. |
| --- |
| b. Cannot be computed because there is insufficient memory. |

**3. I had problems in interacting with the teacher * E learning skills**

| **Crosstab** | | | | | |
| --- | --- | --- | --- | --- | --- |
|  | | | E learning skills | | Total |
|  |  |  | 1 | 2 |  |
| 3. I had problems in interacting with the teacher | 1 | Count | 18 | 6 | 24 |
|  |  | % within E learning skills | 5.5% | 7.2% | 5.9% |
|  | 2 | Count | 111 | 17 | 128 |
|  |  | % within E learning skills | 34.0% | 20.5% | 31.3% |
|  | 3 | Count | 71 | 20 | 91 |
|  |  | % within E learning skills | 21.8% | 24.1% | 22.2% |
|  | 4 | Count | 102 | 31 | 133 |
|  |  | % within E learning skills | 31.3% | 37.3% | 32.5% |
|  | 5 | Count | 24 | 9 | 33 |
|  |  | % within E learning skills | 7.4% | 10.8% | 8.1% |
| Total | | Count | 326 | 83 | 409 |
|  |  | % within E learning skills | 100.0% | 100.0% | 100.0% |

| **Chi-Square Tests** | | | | | | |
| --- | --- | --- | --- | --- | --- | --- |
|  | Value | df | Asymp. Sig. (2-sided) | Exact Sig. (2-sided) | Exact Sig. (1-sided) | Point Probability |
| Pearson Chi-Square | 6.121^a^ | 4 | .190 | .189 |  |  |
| Likelihood Ratio | 6.417 | 4 | .170 | .181 |  |  |
| Fisher's Exact Test | 6.660 |  |  | .151 |  |  |
| Linear-by-Linear Association | 2.973^b^ | 1 | .085 | .092 | .048 | .010 |
| N of Valid Cases | 409 |  |  |  |  |  |

| a. 1 cells (10.0%) have expected count less than 5. The minimum expected count is 4.87. |
| --- |
| b. The standardized statistic is 1.724. |

**4. I had confusion with logging in and operating the online process * Age**

| **Crosstab** | | | | | |
| --- | --- | --- | --- | --- | --- |
|  | | | Age | | Total |
|  |  |  | 1 | 2 |  |
| 4. I had confusion with logging in and operating the online process | 1 | Count | 57 | 5 | 62 |
|  |  | % within Age | 19.0% | 4.6% | 15.2% |
|  | 2 | Count | 139 | 54 | 193 |
|  |  | % within Age | 46.3% | 49.5% | 47.2% |
|  | 3 | Count | 43 | 21 | 64 |
|  |  | % within Age | 14.3% | 19.3% | 15.6% |
|  | 4 | Count | 51 | 21 | 72 |
|  |  | % within Age | 17.0% | 19.3% | 17.6% |
|  | 5 | Count | 10 | 8 | 18 |
|  |  | % within Age | 3.3% | 7.3% | 4.4% |
| Total | | Count | 300 | 109 | 409 |
|  |  | % within Age | 100.0% | 100.0% | 100.0% |

| **Chi-Square Tests** | | | | | | |
| --- | --- | --- | --- | --- | --- | --- |
|  | Value | df | Asymp. Sig. (2-sided) | Exact Sig. (2-sided) | Exact Sig. (1-sided) | Point Probability |
| Pearson Chi-Square | 15.522^a^ | 4 | .004 | .004 |  |  |
| Likelihood Ratio | 18.011 | 4 | .001 | .001 |  |  |
| Fisher's Exact Test | 17.562 |  |  | .001 |  |  |
| Linear-by-Linear Association | 8.790^b^ | 1 | .003 | .003 | .002 | .001 |
| N of Valid Cases | 409 |  |  |  |  |  |

| a. 1 cells (10.0%) have expected count less than 5. The minimum expected count is 4.80. |
| --- |
| b. The standardized statistic is 2.965. |

**4. I had confusion with logging in and operating the online process * Course of study**

| **Crosstab** | | | | | |
| --- | --- | --- | --- | --- | --- |
|  | | | Course of study | | Total |
|  |  |  | 1 | 2 |  |
| 4. I had confusion with logging in and operating the online process | 1 | Count | 59 | 3 | 62 |
|  |  | % within Course of study | 18.3% | 3.5% | 15.2% |
|  | 2 | Count | 149 | 44 | 193 |
|  |  | % within Course of study | 46.1% | 51.2% | 47.2% |
|  | 3 | Count | 47 | 17 | 64 |
|  |  | % within Course of study | 14.6% | 19.8% | 15.6% |
|  | 4 | Count | 54 | 18 | 72 |
|  |  | % within Course of study | 16.7% | 20.9% | 17.6% |
|  | 5 | Count | 14 | 4 | 18 |
|  |  | % within Course of study | 4.3% | 4.7% | 4.4% |
| Total | | Count | 323 | 86 | 409 |
|  |  | % within Course of study | 100.0% | 100.0% | 100.0% |

| **Chi-Square Tests** | | | | | | |
| --- | --- | --- | --- | --- | --- | --- |
|  | Value | df | Asymp. Sig. (2-sided) | Exact Sig. (2-sided) | Exact Sig. (1-sided) | Point Probability |
| Pearson Chi-Square | 12.030^a^ | 4 | .017 | .017 |  |  |
| Likelihood Ratio | 15.331 | 4 | .004 | .005 |  |  |
| Fisher's Exact Test | 14.551 |  |  | .005 |  |  |
| Linear-by-Linear Association | 4.998^b^ | 1 | .025 | .029 | .015 | .004 |
| N of Valid Cases | 409 |  |  |  |  |  |

| a. 1 cells (10.0%) have expected count less than 5. The minimum expected count is 3.78. |
| --- |
| b. The standardized statistic is 2.236. |

**4. I had confusion with logging in and operating the online process * Place**

| **Crosstab** | | | | | | |
| --- | --- | --- | --- | --- | --- | --- |
|  | | | Place | | | Total |
|  |  |  | 1 | 2 | 3 |  |
| 4. I had confusion with logging in and operating the online process | 1 | Count | 15 | 4 | 43 | 62 |
|  |  | % within Place | 9.4% | 6.7% | 22.8% | 15.2% |
|  | 2 | Count | 73 | 28 | 92 | 193 |
|  |  | % within Place | 45.6% | 46.7% | 48.7% | 47.2% |
|  | 3 | Count | 27 | 15 | 22 | 64 |
|  |  | % within Place | 16.9% | 25.0% | 11.6% | 15.6% |
|  | 4 | Count | 38 | 12 | 22 | 72 |
|  |  | % within Place | 23.8% | 20.0% | 11.6% | 17.6% |
|  | 5 | Count | 7 | 1 | 10 | 18 |
|  |  | % within Place | 4.4% | 1.7% | 5.3% | 4.4% |
| Total | | Count | 160 | 60 | 189 | 409 |
|  |  | % within Place | 100.0% | 100.0% | 100.0% | 100.0% |

| **Chi-Square Tests** | | | | | |
| --- | --- | --- | --- | --- | --- |
|  | Value | df | Asymp. Sig. (2-sided) | Exact Sig. (2-sided) | Exact Sig. (1-sided) |
| Pearson Chi-Square | 28.002^a^ | 8 | .000 | .^b^ |  |
| Likelihood Ratio | 28.500 | 8 | .000 | .^b^ |  |
| Fisher's Exact Test | .^b^ |  |  | .^b^ |  |
| Linear-by-Linear Association | 12.112 | 1 | .001 | .^b^ | .^b^ |
| N of Valid Cases | 409 |  |  |  |  |

| a. 1 cells (6.7%) have expected count less than 5. The minimum expected count is 2.64. |
| --- |
| b. Cannot be computed because there is insufficient memory. |

**4. I had confusion with logging in and operating the online process * E learning skills**

| **Crosstab** | | | | | |
| --- | --- | --- | --- | --- | --- |
|  | | | E learning skills | | Total |
|  |  |  | 1 | 2 |  |
| 4. I had confusion with logging in and operating the online process | 1 | Count | 54 | 8 | 62 |
|  |  | % within E learning skills | 16.6% | 9.6% | 15.2% |
|  | 2 | Count | 162 | 31 | 193 |
|  |  | % within E learning skills | 49.7% | 37.3% | 47.2% |
|  | 3 | Count | 51 | 13 | 64 |
|  |  | % within E learning skills | 15.6% | 15.7% | 15.6% |
|  | 4 | Count | 50 | 22 | 72 |
|  |  | % within E learning skills | 15.3% | 26.5% | 17.6% |
|  | 5 | Count | 9 | 9 | 18 |
|  |  | % within E learning skills | 2.8% | 10.8% | 4.4% |
| Total | | Count | 326 | 83 | 409 |
|  |  | % within E learning skills | 100.0% | 100.0% | 100.0% |

| **Chi-Square Tests** | | | | | | |
| --- | --- | --- | --- | --- | --- | --- |
|  | Value | df | Asymp. Sig. (2-sided) | Exact Sig. (2-sided) | Exact Sig. (1-sided) | Point Probability |
| Pearson Chi-Square | 18.738^a^ | 4 | .001 | .001 |  |  |
| Likelihood Ratio | 16.654 | 4 | .002 | .003 |  |  |
| Fisher's Exact Test | 16.890 |  |  | .002 |  |  |
| Linear-by-Linear Association | 16.174^b^ | 1 | .000 | .000 | .000 | .000 |
| N of Valid Cases | 409 |  |  |  |  |  |

| a. 1 cells (10.0%) have expected count less than 5. The minimum expected count is 3.65. |
| --- |
| b. The standardized statistic is 4.022. |

**5. I had problems with sound due to slow internet * Age**

| **Crosstab** | | | | | |
| --- | --- | --- | --- | --- | --- |
|  | | | Age | | Total |
|  |  |  | 1 | 2 |  |
| 5. I had problems with sound due to slow internet | 1 | Count | 22 | 1 | 23 |
|  |  | % within Age | 7.3% | 0.9% | 5.6% |
|  | 2 | Count | 62 | 21 | 83 |
|  |  | % within Age | 20.7% | 19.3% | 20.3% |
|  | 3 | Count | 51 | 8 | 59 |
|  |  | % within Age | 17.0% | 7.3% | 14.4% |
|  | 4 | Count | 113 | 60 | 173 |
|  |  | % within Age | 37.7% | 55.0% | 42.3% |
|  | 5 | Count | 52 | 19 | 71 |
|  |  | % within Age | 17.3% | 17.4% | 17.4% |
| Total | | Count | 300 | 109 | 409 |
|  |  | % within Age | 100.0% | 100.0% | 100.0% |

| **Chi-Square Tests** | | | | | | |
| --- | --- | --- | --- | --- | --- | --- |
|  | Value | df | Asymp. Sig. (2-sided) | Exact Sig. (2-sided) | Exact Sig. (1-sided) | Point Probability |
| Pearson Chi-Square | 16.812^a^ | 4 | .002 | .002 |  |  |
| Likelihood Ratio | 19.473 | 4 | .001 | .001 |  |  |
| Fisher's Exact Test | 17.734 |  |  | .001 |  |  |
| Linear-by-Linear Association | 6.029^b^ | 1 | .014 | .016 | .007 | .002 |
| N of Valid Cases | 409 |  |  |  |  |  |

| a. 0 cells (0.0%) have expected count less than 5. The minimum expected count is 6.13. |
| --- |
| b. The standardized statistic is 2.455. |

**5. I had problems with sound due to slow internet * Course of study**

| **Crosstab** | | | | | |
| --- | --- | --- | --- | --- | --- |
|  | | | Course of study | | Total |
|  |  |  | 1 | 2 |  |
| 5. I had problems with sound due to slow internet | 1 | Count | 21 | 2 | 23 |
|  |  | % within Course of study | 6.5% | 2.3% | 5.6% |
|  | 2 | Count | 65 | 18 | 83 |
|  |  | % within Course of study | 20.1% | 20.9% | 20.3% |
|  | 3 | Count | 51 | 8 | 59 |
|  |  | % within Course of study | 15.8% | 9.3% | 14.4% |
|  | 4 | Count | 128 | 45 | 173 |
|  |  | % within Course of study | 39.6% | 52.3% | 42.3% |
|  | 5 | Count | 58 | 13 | 71 |
|  |  | % within Course of study | 18.0% | 15.1% | 17.4% |
| Total | | Count | 323 | 86 | 409 |
|  |  | % within Course of study | 100.0% | 100.0% | 100.0% |

| **Chi-Square Tests** | | | | | | |
| --- | --- | --- | --- | --- | --- | --- |
|  | Value | df | Asymp. Sig. (2-sided) | Exact Sig. (2-sided) | Exact Sig. (1-sided) | Point Probability |
| Pearson Chi-Square | 7.013^a^ | 4 | .135 | .135 |  |  |
| Likelihood Ratio | 7.562 | 4 | .109 | .116 |  |  |
| Fisher's Exact Test | 6.671 |  |  | .150 |  |  |
| Linear-by-Linear Association | 1.073^b^ | 1 | .300 | .320 | .163 | .025 |
| N of Valid Cases | 409 |  |  |  |  |  |

| a. 1 cells (10.0%) have expected count less than 5. The minimum expected count is 4.84. |
| --- |
| b. The standardized statistic is 1.036. |

**5. I had problems with sound due to slow internet * Place**

| **Crosstab** | | | | | | |
| --- | --- | --- | --- | --- | --- | --- |
|  | | | Place | | | Total |
|  |  |  | 1 | 2 | 3 |  |
| 5. I had problems with sound due to slow internet | 1 | Count | 6 | 3 | 14 | 23 |
|  |  | % within Place | 3.8% | 5.0% | 7.4% | 5.6% |
|  | 2 | Count | 29 | 11 | 43 | 83 |
|  |  | % within Place | 18.1% | 18.3% | 22.8% | 20.3% |
|  | 3 | Count | 18 | 14 | 27 | 59 |
|  |  | % within Place | 11.2% | 23.3% | 14.3% | 14.4% |
|  | 4 | Count | 81 | 22 | 70 | 173 |
|  |  | % within Place | 50.6% | 36.7% | 37.0% | 42.3% |
|  | 5 | Count | 26 | 10 | 35 | 71 |
|  |  | % within Place | 16.2% | 16.7% | 18.5% | 17.4% |
| Total | | Count | 160 | 60 | 189 | 409 |
|  |  | % within Place | 100.0% | 100.0% | 100.0% | 100.0% |

| **Chi-Square Tests** | | | | | |
| --- | --- | --- | --- | --- | --- |
|  | Value | df | Asymp. Sig. (2-sided) | Exact Sig. (2-sided) | Exact Sig. (1-sided) |
| Pearson Chi-Square | 12.164^a^ | 8 | .144 | .^b^ |  |
| Likelihood Ratio | 11.684 | 8 | .166 | .^b^ |  |
| Fisher's Exact Test | .^b^ |  |  | .^b^ |  |
| Linear-by-Linear Association | 2.813 | 1 | .093 | .^b^ | .^b^ |
| N of Valid Cases | 409 |  |  |  |  |

| a. 1 cells (6.7%) have expected count less than 5. The minimum expected count is 3.37. |
| --- |
| b. Cannot be computed because there is insufficient memory. |

**5. I had problems with sound due to slow internet * E learning skills**

| **Crosstab** | | | | | |
| --- | --- | --- | --- | --- | --- |
|  | | | E learning skills | | Total |
|  |  |  | 1 | 2 |  |
| 5. I had problems with sound due to slow internet | 1 | Count | 16 | 7 | 23 |
|  |  | % within E learning skills | 4.9% | 8.4% | 5.6% |
|  | 2 | Count | 69 | 14 | 83 |
|  |  | % within E learning skills | 21.2% | 16.9% | 20.3% |
|  | 3 | Count | 51 | 8 | 59 |
|  |  | % within E learning skills | 15.6% | 9.6% | 14.4% |
|  | 4 | Count | 142 | 31 | 173 |
|  |  | % within E learning skills | 43.6% | 37.3% | 42.3% |
|  | 5 | Count | 48 | 23 | 71 |
|  |  | % within E learning skills | 14.7% | 27.7% | 17.4% |
| Total | | Count | 326 | 83 | 409 |
|  |  | % within E learning skills | 100.0% | 100.0% | 100.0% |

| **Chi-Square Tests** | | | | | | |
| --- | --- | --- | --- | --- | --- | --- |
|  | Value | df | Asymp. Sig. (2-sided) | Exact Sig. (2-sided) | Exact Sig. (1-sided) | Point Probability |
| Pearson Chi-Square | 10.749^a^ | 4 | .030 | .029 |  |  |
| Likelihood Ratio | 10.099 | 4 | .039 | .043 |  |  |
| Fisher's Exact Test | 10.183 |  |  | .035 |  |  |
| Linear-by-Linear Association | 1.427^b^ | 1 | .232 | .244 | .127 | .021 |
| N of Valid Cases | 409 |  |  |  |  |  |

| a. 1 cells (10.0%) have expected count less than 5. The minimum expected count is 4.67. |
| --- |
| b. The standardized statistic is 1.195. |

**6. Joint or group study is difficult in online learning * Age**

| **Crosstab** | | | | | |
| --- | --- | --- | --- | --- | --- |
|  | | | Age | | Total |
|  |  |  | 1 | 2 |  |
| 6. Joint or group study is difficult in online learning | 1 | Count | 14 | 1 | 15 |
|  |  | % within Age | 4.7% | 0.9% | 3.7% |
|  | 2 | Count | 61 | 14 | 75 |
|  |  | % within Age | 20.3% | 12.8% | 18.3% |
|  | 3 | Count | 52 | 14 | 66 |
|  |  | % within Age | 17.3% | 12.8% | 16.1% |
|  | 4 | Count | 119 | 53 | 172 |
|  |  | % within Age | 39.7% | 48.6% | 42.1% |
|  | 5 | Count | 54 | 27 | 81 |
|  |  | % within Age | 18.0% | 24.8% | 19.8% |
| Total | | Count | 300 | 109 | 409 |
|  |  | % within Age | 100.0% | 100.0% | 100.0% |

| **Chi-Square Tests** | | | | | | |
| --- | --- | --- | --- | --- | --- | --- |
|  | Value | df | Asymp. Sig. (2-sided) | Exact Sig. (2-sided) | Exact Sig. (1-sided) | Point Probability |
| Pearson Chi-Square | 9.884^a^ | 4 | .042 | .042 |  |  |
| Likelihood Ratio | 10.902 | 4 | .028 | .032 |  |  |
| Fisher's Exact Test | 9.760 |  |  | .042 |  |  |
| Linear-by-Linear Association | 9.117^b^ | 1 | .003 | .003 | .001 | .000 |
| N of Valid Cases | 409 |  |  |  |  |  |

| a. 1 cells (10.0%) have expected count less than 5. The minimum expected count is 4.00. |
| --- |
| b. The standardized statistic is 3.019. |

**6. Joint or group study is difficult in online learning * Course of study**

| **Crosstab** | | | | | |
| --- | --- | --- | --- | --- | --- |
|  | | | Course of study | | Total |
|  |  |  | 1 | 2 |  |
| 6. Joint or group study is difficult in online learning | 1 | Count | 12 | 3 | 15 |
|  |  | % within Course of study | 3.7% | 3.5% | 3.7% |
|  | 2 | Count | 62 | 13 | 75 |
|  |  | % within Course of study | 19.2% | 15.1% | 18.3% |
|  | 3 | Count | 56 | 10 | 66 |
|  |  | % within Course of study | 17.3% | 11.6% | 16.1% |
|  | 4 | Count | 135 | 37 | 172 |
|  |  | % within Course of study | 41.8% | 43.0% | 42.1% |
|  | 5 | Count | 58 | 23 | 81 |
|  |  | % within Course of study | 18.0% | 26.7% | 19.8% |
| Total | | Count | 323 | 86 | 409 |
|  |  | % within Course of study | 100.0% | 100.0% | 100.0% |

| **Chi-Square Tests** | | | | | | |
| --- | --- | --- | --- | --- | --- | --- |
|  | Value | df | Asymp. Sig. (2-sided) | Exact Sig. (2-sided) | Exact Sig. (1-sided) | Point Probability |
| Pearson Chi-Square | 4.670^a^ | 4 | .323 | .324 |  |  |
| Likelihood Ratio | 4.620 | 4 | .329 | .339 |  |  |
| Fisher's Exact Test | 4.498 |  |  | .340 |  |  |
| Linear-by-Linear Association | 3.001^b^ | 1 | .083 | .090 | .046 | .010 |
| N of Valid Cases | 409 |  |  |  |  |  |

| a. 1 cells (10.0%) have expected count less than 5. The minimum expected count is 3.15. |
| --- |
| b. The standardized statistic is 1.732. |

**6. Joint or group study is difficult in online learning * Place**

| **Crosstab** | | | | | | |
| --- | --- | --- | --- | --- | --- | --- |
|  | | | Place | | | Total |
|  |  |  | 1 | 2 | 3 |  |
| 6. Joint or group study is difficult in online learning | 1 | Count | 2 | 1 | 12 | 15 |
|  |  | % within Place | 1.2% | 1.7% | 6.3% | 3.7% |
|  | 2 | Count | 27 | 15 | 33 | 75 |
|  |  | % within Place | 16.9% | 25.0% | 17.5% | 18.3% |
|  | 3 | Count | 17 | 12 | 37 | 66 |
|  |  | % within Place | 10.6% | 20.0% | 19.6% | 16.1% |
|  | 4 | Count | 78 | 21 | 73 | 172 |
|  |  | % within Place | 48.8% | 35.0% | 38.6% | 42.1% |
|  | 5 | Count | 36 | 11 | 34 | 81 |
|  |  | % within Place | 22.5% | 18.3% | 18.0% | 19.8% |
| Total | | Count | 160 | 60 | 189 | 409 |
|  |  | % within Place | 100.0% | 100.0% | 100.0% | 100.0% |

| **Chi-Square Tests** | | | | | |
| --- | --- | --- | --- | --- | --- |
|  | Value | df | Asymp. Sig. (2-sided) | Exact Sig. (2-sided) | Exact Sig. (1-sided) |
| Pearson Chi-Square | 17.494^a^ | 8 | .025 | .^b^ |  |
| Likelihood Ratio | 17.978 | 8 | .021 | .^b^ |  |
| Fisher's Exact Test | .^b^ |  |  | .^b^ |  |
| Linear-by-Linear Association | 6.140 | 1 | .013 | .^b^ | .^b^ |
| N of Valid Cases | 409 |  |  |  |  |

| a. 1 cells (6.7%) have expected count less than 5. The minimum expected count is 2.20. |
| --- |
| b. Cannot be computed because there is insufficient memory. |

**6. Joint or group study is difficult in online learning * E learning skills**

| **Crosstab** | | | | | |
| --- | --- | --- | --- | --- | --- |
|  | | | E learning skills | | Total |
|  |  |  | 1 | 2 |  |
| 6. Joint or group study is difficult in online learning | 1 | Count | 11 | 4 | 15 |
|  |  | % within E learning skills | 3.4% | 4.8% | 3.7% |
|  | 2 | Count | 64 | 11 | 75 |
|  |  | % within E learning skills | 19.6% | 13.3% | 18.3% |
|  | 3 | Count | 56 | 10 | 66 |
|  |  | % within E learning skills | 17.2% | 12.0% | 16.1% |
|  | 4 | Count | 137 | 35 | 172 |
|  |  | % within E learning skills | 42.0% | 42.2% | 42.1% |
|  | 5 | Count | 58 | 23 | 81 |
|  |  | % within E learning skills | 17.8% | 27.7% | 19.8% |
| Total | | Count | 326 | 83 | 409 |
|  |  | % within E learning skills | 100.0% | 100.0% | 100.0% |

| **Chi-Square Tests** | | | | | | |
| --- | --- | --- | --- | --- | --- | --- |
|  | Value | df | Asymp. Sig. (2-sided) | Exact Sig. (2-sided) | Exact Sig. (1-sided) | Point Probability |
| Pearson Chi-Square | 6.211^a^ | 4 | .184 | .183 |  |  |
| Likelihood Ratio | 6.115 | 4 | .191 | .206 |  |  |
| Fisher's Exact Test | 6.139 |  |  | .184 |  |  |
| Linear-by-Linear Association | 2.958^b^ | 1 | .085 | .086 | .047 | .010 |
| N of Valid Cases | 409 |  |  |  |  |  |

| a. 1 cells (10.0%) have expected count less than 5. The minimum expected count is 3.04. |
| --- |
| b. The standardized statistic is 1.720. |

**7. Online learning is not suitable for subjects like mathematics, accounts, laboratory based subjects (biology, chemistry, physics) * Age**

| **Crosstab** | | | | | |
| --- | --- | --- | --- | --- | --- |
|  | | | Age | | Total |
|  |  |  | 1 | 2 |  |
| 7. Online learning is not suitable for subjects like mathematics, accounts, laboratory based subjects (biology, chemistry, physics) | 1 | Count | 14 | 1 | 15 |
|  |  | % within Age | 4.7% | 0.9% | 3.7% |
|  | 2 | Count | 40 | 12 | 52 |
|  |  | % within Age | 13.3% | 11.0% | 12.7% |
|  | 3 | Count | 58 | 16 | 74 |
|  |  | % within Age | 19.3% | 14.7% | 18.1% |
|  | 4 | Count | 92 | 45 | 137 |
|  |  | % within Age | 30.7% | 41.3% | 33.5% |
|  | 5 | Count | 96 | 35 | 131 |
|  |  | % within Age | 32.0% | 32.1% | 32.0% |
| Total | | Count | 300 | 109 | 409 |
|  |  | % within Age | 100.0% | 100.0% | 100.0% |

| **Chi-Square Tests** | | | | | | |
| --- | --- | --- | --- | --- | --- | --- |
|  | Value | df | Asymp. Sig. (2-sided) | Exact Sig. (2-sided) | Exact Sig. (1-sided) | Point Probability |
| Pearson Chi-Square | 7.053^a^ | 4 | .133 | .132 |  |  |
| Likelihood Ratio | 7.901 | 4 | .095 | .102 |  |  |
| Fisher's Exact Test | 6.836 |  |  | .142 |  |  |
| Linear-by-Linear Association | 2.641^b^ | 1 | .104 | .105 | .056 | .011 |
| N of Valid Cases | 409 |  |  |  |  |  |

| a. 1 cells (10.0%) have expected count less than 5. The minimum expected count is 4.00. |
| --- |
| b. The standardized statistic is 1.625. |

**7. Online learning is not suitable for subjects like mathematics, accounts, laboratory based subjects (biology, chemistry, physics) * Course of study**

| **Crosstab** | | | | | |
| --- | --- | --- | --- | --- | --- |
|  | | | Course of study | | Total |
|  |  |  | 1 | 2 |  |
| 7. Online learning is not suitable for subjects like mathematics, accounts, laboratory based subjects (biology, chemistry, physics) | 1 | Count | 13 | 2 | 15 |
|  |  | % within Course of study | 4.0% | 2.3% | 3.7% |
|  | 2 | Count | 44 | 8 | 52 |
|  |  | % within Course of study | 13.6% | 9.3% | 12.7% |
|  | 3 | Count | 62 | 12 | 74 |
|  |  | % within Course of study | 19.2% | 14.0% | 18.1% |
|  | 4 | Count | 105 | 32 | 137 |
|  |  | % within Course of study | 32.5% | 37.2% | 33.5% |
|  | 5 | Count | 99 | 32 | 131 |
|  |  | % within Course of study | 30.7% | 37.2% | 32.0% |
| Total | | Count | 323 | 86 | 409 |
|  |  | % within Course of study | 100.0% | 100.0% | 100.0% |

| **Chi-Square Tests** | | | | | | |
| --- | --- | --- | --- | --- | --- | --- |
|  | Value | df | Asymp. Sig. (2-sided) | Exact Sig. (2-sided) | Exact Sig. (1-sided) | Point Probability |
| Pearson Chi-Square | 3.923^a^ | 4 | .416 | .421 |  |  |
| Likelihood Ratio | 4.082 | 4 | .395 | .406 |  |  |
| Fisher's Exact Test | 3.586 |  |  | .463 |  |  |
| Linear-by-Linear Association | 3.427^b^ | 1 | .064 | .069 | .035 | .008 |
| N of Valid Cases | 409 |  |  |  |  |  |

| a. 1 cells (10.0%) have expected count less than 5. The minimum expected count is 3.15. |
| --- |
| b. The standardized statistic is 1.851. |

**7. Online learning is not suitable for subjects like mathematics, accounts, laboratory based subjects (biology, chemistry, physics) * Place**

| **Crosstab** | | | | | | |
| --- | --- | --- | --- | --- | --- | --- |
|  | | | Place | | | Total |
|  |  |  | 1 | 2 | 3 |  |
| 7. Online learning is not suitable for subjects like mathematics, accounts, laboratory based subjects (biology, chemistry, physics) | 1 | Count | 5 | 1 | 9 | 15 |
|  |  | % within Place | 3.1% | 1.7% | 4.8% | 3.7% |
|  | 2 | Count | 22 | 8 | 22 | 52 |
|  |  | % within Place | 13.8% | 13.3% | 11.6% | 12.7% |
|  | 3 | Count | 26 | 12 | 36 | 74 |
|  |  | % within Place | 16.2% | 20.0% | 19.0% | 18.1% |
|  | 4 | Count | 62 | 19 | 56 | 137 |
|  |  | % within Place | 38.8% | 31.7% | 29.6% | 33.5% |
|  | 5 | Count | 45 | 20 | 66 | 131 |
|  |  | % within Place | 28.1% | 33.3% | 34.9% | 32.0% |
| Total | | Count | 160 | 60 | 189 | 409 |
|  |  | % within Place | 100.0% | 100.0% | 100.0% | 100.0% |

| **Chi-Square Tests** | | | | | |
| --- | --- | --- | --- | --- | --- |
|  | Value | df | Asymp. Sig. (2-sided) | Exact Sig. (2-sided) | Exact Sig. (1-sided) |
| Pearson Chi-Square | 5.750^a^ | 8 | .675 | .681 |  |
| Likelihood Ratio | 5.868 | 8 | .662 | .682 |  |
| Fisher's Exact Test | 5.506 |  |  | .705 |  |
| Linear-by-Linear Association | .068 | 1 | .794 | .^b^ | .^b^ |
| N of Valid Cases | 409 |  |  |  |  |

| a. 1 cells (6.7%) have expected count less than 5. The minimum expected count is 2.20. |
| --- |
| b. Cannot be computed because there is insufficient memory. |

**7. Online learning is not suitable for subjects like mathematics, accounts, laboratory based subjects (biology, chemistry, physics) * E learning skills**

| **Crosstab** | | | | | |
| --- | --- | --- | --- | --- | --- |
|  | | | E learning skills | | Total |
|  |  |  | 1 | 2 |  |
| 7. Online learning is not suitable for subjects like mathematics, accounts, laboratory based subjects (biology, chemistry, physics) | 1 | Count | 11 | 4 | 15 |
|  |  | % within E learning skills | 3.4% | 4.8% | 3.7% |
|  | 2 | Count | 48 | 4 | 52 |
|  |  | % within E learning skills | 14.7% | 4.8% | 12.7% |
|  | 3 | Count | 61 | 13 | 74 |
|  |  | % within E learning skills | 18.7% | 15.7% | 18.1% |
|  | 4 | Count | 109 | 28 | 137 |
|  |  | % within E learning skills | 33.4% | 33.7% | 33.5% |
|  | 5 | Count | 97 | 34 | 131 |
|  |  | % within E learning skills | 29.8% | 41.0% | 32.0% |
| Total | | Count | 326 | 83 | 409 |
|  |  | % within E learning skills | 100.0% | 100.0% | 100.0% |

| **Chi-Square Tests** | | | | | | |
| --- | --- | --- | --- | --- | --- | --- |
|  | Value | df | Asymp. Sig. (2-sided) | Exact Sig. (2-sided) | Exact Sig. (1-sided) | Point Probability |
| Pearson Chi-Square | 8.418^a^ | 4 | .077 | .076 |  |  |
| Likelihood Ratio | 9.474 | 4 | .050 | .059 |  |  |
| Fisher's Exact Test | 9.018 |  |  | .056 |  |  |
| Linear-by-Linear Association | 4.525^b^ | 1 | .033 | .035 | .018 | .004 |
| N of Valid Cases | 409 |  |  |  |  |  |

| a. 1 cells (10.0%) have expected count less than 5. The minimum expected count is 3.04. |
| --- |
| b. The standardized statistic is 2.127. |

**8. Online learning can cause isolation and lack of belonging and support * Age**

| **Crosstab** | | | | | |
| --- | --- | --- | --- | --- | --- |
|  | | | Age | | Total |
|  |  |  | 1 | 2 |  |
| 8. Online learning can cause isolation and lack of belonging and support | 1 | Count | 17 | 3 | 20 |
|  |  | % within Age | 5.7% | 2.8% | 4.9% |
|  | 2 | Count | 45 | 16 | 61 |
|  |  | % within Age | 15.0% | 14.7% | 14.9% |
|  | 3 | Count | 79 | 22 | 101 |
|  |  | % within Age | 26.3% | 20.2% | 24.7% |
|  | 4 | Count | 99 | 45 | 144 |
|  |  | % within Age | 33.0% | 41.3% | 35.2% |
|  | 5 | Count | 60 | 23 | 83 |
|  |  | % within Age | 20.0% | 21.1% | 20.3% |
| Total | | Count | 300 | 109 | 409 |
|  |  | % within Age | 100.0% | 100.0% | 100.0% |

| **Chi-Square Tests** | | | | | | |
| --- | --- | --- | --- | --- | --- | --- |
|  | Value | df | Asymp. Sig. (2-sided) | Exact Sig. (2-sided) | Exact Sig. (1-sided) | Point Probability |
| Pearson Chi-Square | 4.225^a^ | 4 | .376 | .379 |  |  |
| Likelihood Ratio | 4.401 | 4 | .354 | .364 |  |  |
| Fisher's Exact Test | 4.032 |  |  | .401 |  |  |
| Linear-by-Linear Association | 1.769^b^ | 1 | .183 | .194 | .100 | .017 |
| N of Valid Cases | 409 |  |  |  |  |  |

| a. 0 cells (0.0%) have expected count less than 5. The minimum expected count is 5.33. |
| --- |
| b. The standardized statistic is 1.330. |

**8. Online learning can cause isolation and lack of belonging and support * Course of study**

| **Crosstab** | | | | | |
| --- | --- | --- | --- | --- | --- |
|  | | | Course of study | | Total |
|  |  |  | 1 | 2 |  |
| 8. Online learning can cause isolation and lack of belonging and support | 1 | Count | 14 | 6 | 20 |
|  |  | % within Course of study | 4.3% | 7.0% | 4.9% |
|  | 2 | Count | 50 | 11 | 61 |
|  |  | % within Course of study | 15.5% | 12.8% | 14.9% |
|  | 3 | Count | 85 | 16 | 101 |
|  |  | % within Course of study | 26.3% | 18.6% | 24.7% |
|  | 4 | Count | 106 | 38 | 144 |
|  |  | % within Course of study | 32.8% | 44.2% | 35.2% |
|  | 5 | Count | 68 | 15 | 83 |
|  |  | % within Course of study | 21.1% | 17.4% | 20.3% |
| Total | | Count | 323 | 86 | 409 |
|  |  | % within Course of study | 100.0% | 100.0% | 100.0% |

| **Chi-Square Tests** | | | | | | |
| --- | --- | --- | --- | --- | --- | --- |
|  | Value | df | Asymp. Sig. (2-sided) | Exact Sig. (2-sided) | Exact Sig. (1-sided) | Point Probability |
| Pearson Chi-Square | 5.864^a^ | 4 | .210 | .210 |  |  |
| Likelihood Ratio | 5.788 | 4 | .216 | .226 |  |  |
| Fisher's Exact Test | 5.801 |  |  | .210 |  |  |
| Linear-by-Linear Association | .013^b^ | 1 | .909 | .914 | .478 | .043 |
| N of Valid Cases | 409 |  |  |  |  |  |

| a. 1 cells (10.0%) have expected count less than 5. The minimum expected count is 4.21. |
| --- |
| b. The standardized statistic is .114. |

**8. Online learning can cause isolation and lack of belonging and support * Place**

| **Crosstab** | | | | | | |
| --- | --- | --- | --- | --- | --- | --- |
|  | | | Place | | | Total |
|  |  |  | 1 | 2 | 3 |  |
| 8. Online learning can cause isolation and lack of belonging and support | 1 | Count | 5 | 1 | 14 | 20 |
|  |  | % within Place | 3.1% | 1.7% | 7.4% | 4.9% |
|  | 2 | Count | 25 | 11 | 25 | 61 |
|  |  | % within Place | 15.6% | 18.3% | 13.2% | 14.9% |
|  | 3 | Count | 42 | 15 | 44 | 101 |
|  |  | % within Place | 26.2% | 25.0% | 23.3% | 24.7% |
|  | 4 | Count | 60 | 22 | 62 | 144 |
|  |  | % within Place | 37.5% | 36.7% | 32.8% | 35.2% |
|  | 5 | Count | 28 | 11 | 44 | 83 |
|  |  | % within Place | 17.5% | 18.3% | 23.3% | 20.3% |
| Total | | Count | 160 | 60 | 189 | 409 |
|  |  | % within Place | 100.0% | 100.0% | 100.0% | 100.0% |

| **Chi-Square Tests** | | | | | |
| --- | --- | --- | --- | --- | --- |
|  | Value | df | Asymp. Sig. (2-sided) | Exact Sig. (2-sided) | Exact Sig. (1-sided) |
| Pearson Chi-Square | 8.086^a^ | 8 | .425 | .^b^ |  |
| Likelihood Ratio | 8.319 | 8 | .403 | .^b^ |  |
| Fisher's Exact Test | .^b^ |  |  | .^b^ |  |
| Linear-by-Linear Association | .003 | 1 | .955 | .^b^ | .^b^ |
| N of Valid Cases | 409 |  |  |  |  |

| a. 1 cells (6.7%) have expected count less than 5. The minimum expected count is 2.93. |
| --- |
| b. Cannot be computed because there is insufficient memory. |

**8. Online learning can cause isolation and lack of belonging and support * E learning skills**

| **Crosstab** | | | | | |
| --- | --- | --- | --- | --- | --- |
|  | | | E learning skills | | Total |
|  |  |  | 1 | 2 |  |
| 8. Online learning can cause isolation and lack of belonging and support | 1 | Count | 15 | 5 | 20 |
|  |  | % within E learning skills | 4.6% | 6.0% | 4.9% |
|  | 2 | Count | 53 | 8 | 61 |
|  |  | % within E learning skills | 16.3% | 9.6% | 14.9% |
|  | 3 | Count | 82 | 19 | 101 |
|  |  | % within E learning skills | 25.2% | 22.9% | 24.7% |
|  | 4 | Count | 111 | 33 | 144 |
|  |  | % within E learning skills | 34.0% | 39.8% | 35.2% |
|  | 5 | Count | 65 | 18 | 83 |
|  |  | % within E learning skills | 19.9% | 21.7% | 20.3% |
| Total | | Count | 326 | 83 | 409 |
|  |  | % within E learning skills | 100.0% | 100.0% | 100.0% |

| **Chi-Square Tests** | | | | | | |
| --- | --- | --- | --- | --- | --- | --- |
|  | Value | df | Asymp. Sig. (2-sided) | Exact Sig. (2-sided) | Exact Sig. (1-sided) | Point Probability |
| Pearson Chi-Square | 3.067^a^ | 4 | .547 | .553 |  |  |
| Likelihood Ratio | 3.248 | 4 | .517 | .527 |  |  |
| Fisher's Exact Test | 3.204 |  |  | .528 |  |  |
| Linear-by-Linear Association | .891^b^ | 1 | .345 | .351 | .188 | .028 |
| N of Valid Cases | 409 |  |  |  |  |  |

| a. 1 cells (10.0%) have expected count less than 5. The minimum expected count is 4.06. |
| --- |
| b. The standardized statistic is .944. |

**1. I prefer the classroom teaching over online teaching because there is more involvement with teachers and classmates. This is not there in online teaching * Age**

| **Crosstab** | | | | | |
| --- | --- | --- | --- | --- | --- |
|  | | | Age | | Total |
|  |  |  | 1 | 2 |  |
| 1. I prefer the classroom teaching over online teaching because there is more involvement with teachers and classmates. This is not there in online teaching | 1 | Count | 12 | 3 | 15 |
|  |  | % within Age | 4.0% | 2.8% | 3.7% |
|  | 2 | Count | 15 | 10 | 25 |
|  |  | % within Age | 5.0% | 9.2% | 6.1% |
|  | 3 | Count | 33 | 15 | 48 |
|  |  | % within Age | 11.0% | 13.8% | 11.7% |
|  | 4 | Count | 133 | 47 | 180 |
|  |  | % within Age | 44.3% | 43.1% | 44.0% |
|  | 5 | Count | 107 | 34 | 141 |
|  |  | % within Age | 35.7% | 31.2% | 34.5% |
| Total | | Count | 300 | 109 | 409 |
|  |  | % within Age | 100.0% | 100.0% | 100.0% |

| **Chi-Square Tests** | | | | | | |
| --- | --- | --- | --- | --- | --- | --- |
|  | Value | df | Asymp. Sig. (2-sided) | Exact Sig. (2-sided) | Exact Sig. (1-sided) | Point Probability |
| Pearson Chi-Square | 3.629^a^ | 4 | .459 | .462 |  |  |
| Likelihood Ratio | 3.459 | 4 | .484 | .499 |  |  |
| Fisher's Exact Test | 3.578 |  |  | .466 |  |  |
| Linear-by-Linear Association | 1.079^b^ | 1 | .299 | .324 | .163 | .025 |
| N of Valid Cases | 409 |  |  |  |  |  |

| a. 1 cells (10.0%) have expected count less than 5. The minimum expected count is 4.00. |
| --- |
| b. The standardized statistic is -1.039. |

**1. I prefer the classroom teaching over online teaching because there is more involvement with teachers and classmates. This is not there in online teaching * Course of study**

| **Crosstab** | | | | | |
| --- | --- | --- | --- | --- | --- |
|  | | | Course of study | | Total |
|  |  |  | 1 | 2 |  |
| 1. I prefer the classroom teaching over online teaching because there is more involvement with teachers and classmates. This is not there in online teaching | 1 | Count | 12 | 3 | 15 |
|  |  | % within Course of study | 3.7% | 3.5% | 3.7% |
|  | 2 | Count | 18 | 7 | 25 |
|  |  | % within Course of study | 5.6% | 8.1% | 6.1% |
|  | 3 | Count | 39 | 9 | 48 |
|  |  | % within Course of study | 12.1% | 10.5% | 11.7% |
|  | 4 | Count | 139 | 41 | 180 |
|  |  | % within Course of study | 43.0% | 47.7% | 44.0% |
|  | 5 | Count | 115 | 26 | 141 |
|  |  | % within Course of study | 35.6% | 30.2% | 34.5% |
| Total | | Count | 323 | 86 | 409 |
|  |  | % within Course of study | 100.0% | 100.0% | 100.0% |

| **Chi-Square Tests** | | | | | | |
| --- | --- | --- | --- | --- | --- | --- |
|  | Value | df | Asymp. Sig. (2-sided) | Exact Sig. (2-sided) | Exact Sig. (1-sided) | Point Probability |
| Pearson Chi-Square | 1.792^a^ | 4 | .774 | .779 |  |  |
| Likelihood Ratio | 1.758 | 4 | .780 | .789 |  |  |
| Fisher's Exact Test | 1.900 |  |  | .760 |  |  |
| Linear-by-Linear Association | .441^b^ | 1 | .507 | .513 | .270 | .037 |
| N of Valid Cases | 409 |  |  |  |  |  |

| a. 1 cells (10.0%) have expected count less than 5. The minimum expected count is 3.15. |
| --- |
| b. The standardized statistic is -.664. |

**1. I prefer the classroom teaching over online teaching because there is more involvement with teachers and classmates. This is not there in online teaching * Place**

| **Crosstab** | | | | | | |
| --- | --- | --- | --- | --- | --- | --- |
|  | | | Place | | | Total |
|  |  |  | 1 | 2 | 3 |  |
| 1. I prefer the classroom teaching over online teaching because there is more involvement with teachers and classmates. This is not there in online teaching | 1 | Count | 7 | 4 | 4 | 15 |
|  |  | % within Place | 4.4% | 6.7% | 2.1% | 3.7% |
|  | 2 | Count | 7 | 5 | 13 | 25 |
|  |  | % within Place | 4.4% | 8.3% | 6.9% | 6.1% |
|  | 3 | Count | 20 | 8 | 20 | 48 |
|  |  | % within Place | 12.5% | 13.3% | 10.6% | 11.7% |
|  | 4 | Count | 69 | 26 | 85 | 180 |
|  |  | % within Place | 43.1% | 43.3% | 45.0% | 44.0% |
|  | 5 | Count | 57 | 17 | 67 | 141 |
|  |  | % within Place | 35.6% | 28.3% | 35.4% | 34.5% |
| Total | | Count | 160 | 60 | 189 | 409 |
|  |  | % within Place | 100.0% | 100.0% | 100.0% | 100.0% |

| **Chi-Square Tests** | | | | | | |
| --- | --- | --- | --- | --- | --- | --- |
|  | Value | df | Asymp. Sig. (2-sided) | Exact Sig. (2-sided) | Exact Sig. (1-sided) | Point Probability |
| Pearson Chi-Square | 5.655^a^ | 8 | .686 | .692 |  |  |
| Likelihood Ratio | 5.650 | 8 | .686 | .709 |  |  |
| Fisher's Exact Test | 6.118 |  |  | .633 |  |  |
| Linear-by-Linear Association | .141^b^ | 1 | .707 | .713 | .363 | .020 |
| N of Valid Cases | 409 |  |  |  |  |  |

| a. 2 cells (13.3%) have expected count less than 5. The minimum expected count is 2.20. |
| --- |
| b. The standardized statistic is .376. |

**1. I prefer the classroom teaching over online teaching because there is more involvement with teachers and classmates. This is not there in online teaching * E learning skills**

| **Crosstab** | | | | | |
| --- | --- | --- | --- | --- | --- |
|  | | | E learning skills | | Total |
|  |  |  | 1 | 2 |  |
| 1. I prefer the classroom teaching over online teaching because there is more involvement with teachers and classmates. This is not there in online teaching | 1 | Count | 11 | 4 | 15 |
|  |  | % within E learning skills | 3.4% | 4.8% | 3.7% |
|  | 2 | Count | 21 | 4 | 25 |
|  |  | % within E learning skills | 6.4% | 4.8% | 6.1% |
|  | 3 | Count | 40 | 8 | 48 |
|  |  | % within E learning skills | 12.3% | 9.6% | 11.7% |
|  | 4 | Count | 143 | 37 | 180 |
|  |  | % within E learning skills | 43.9% | 44.6% | 44.0% |
|  | 5 | Count | 111 | 30 | 141 |
|  |  | % within E learning skills | 34.0% | 36.1% | 34.5% |
| Total | | Count | 326 | 83 | 409 |
|  |  | % within E learning skills | 100.0% | 100.0% | 100.0% |

| **Chi-Square Tests** | | | | | | |
| --- | --- | --- | --- | --- | --- | --- |
|  | Value | df | Asymp. Sig. (2-sided) | Exact Sig. (2-sided) | Exact Sig. (1-sided) | Point Probability |
| Pearson Chi-Square | 1.144^a^ | 4 | .887 | .888 |  |  |
| Likelihood Ratio | 1.154 | 4 | .886 | .889 |  |  |
| Fisher's Exact Test | 1.169 |  |  | .888 |  |  |
| Linear-by-Linear Association | .084^b^ | 1 | .772 | .810 | .415 | .047 |
| N of Valid Cases | 409 |  |  |  |  |  |

| a. 1 cells (10.0%) have expected count less than 5. The minimum expected count is 3.04. |
| --- |
| b. The standardized statistic is .290. |

**2. I am very much used to classroom studying and feel online teaching/learning is not very useful. * Age**

| **Crosstab** | | | | | |
| --- | --- | --- | --- | --- | --- |
|  | | | Age | | Total |
|  |  |  | 1 | 2 |  |
| 2. I am very much used to classroom studying and feel online teaching/learning is not very useful. | 1 | Count | 16 | 5 | 21 |
|  |  | % within Age | 5.3% | 4.6% | 5.1% |
|  | 2 | Count | 54 | 29 | 83 |
|  |  | % within Age | 18.0% | 26.6% | 20.3% |
|  | 3 | Count | 63 | 18 | 81 |
|  |  | % within Age | 21.0% | 16.5% | 19.8% |
|  | 4 | Count | 70 | 25 | 95 |
|  |  | % within Age | 23.3% | 22.9% | 23.2% |
|  | 5 | Count | 97 | 32 | 129 |
|  |  | % within Age | 32.3% | 29.4% | 31.5% |
| Total | | Count | 300 | 109 | 409 |
|  |  | % within Age | 100.0% | 100.0% | 100.0% |

| **Chi-Square Tests** | | | | | | |
| --- | --- | --- | --- | --- | --- | --- |
|  | Value | df | Asymp. Sig. (2-sided) | Exact Sig. (2-sided) | Exact Sig. (1-sided) | Point Probability |
| Pearson Chi-Square | 4.047^a^ | 4 | .400 | .402 |  |  |
| Likelihood Ratio | 3.922 | 4 | .417 | .425 |  |  |
| Fisher's Exact Test | 3.884 |  |  | .421 |  |  |
| Linear-by-Linear Association | .908^b^ | 1 | .341 | .353 | .182 | .022 |
| N of Valid Cases | 409 |  |  |  |  |  |

| a. 0 cells (0.0%) have expected count less than 5. The minimum expected count is 5.60. |
| --- |
| b. The standardized statistic is -.953. |

**2. I am very much used to classroom studying and feel online teaching/learning is not very useful. * Course of study**

| **Crosstab** | | | | | |
| --- | --- | --- | --- | --- | --- |
|  | | | Course of study | | Total |
|  |  |  | 1 | 2 |  |
| 2. I am very much used to classroom studying and feel online teaching/learning is not very useful. | 1 | Count | 15 | 6 | 21 |
|  |  | % within Course of study | 4.6% | 7.0% | 5.1% |
|  | 2 | Count | 66 | 17 | 83 |
|  |  | % within Course of study | 20.4% | 19.8% | 20.3% |
|  | 3 | Count | 71 | 10 | 81 |
|  |  | % within Course of study | 22.0% | 11.6% | 19.8% |
|  | 4 | Count | 74 | 21 | 95 |
|  |  | % within Course of study | 22.9% | 24.4% | 23.2% |
|  | 5 | Count | 97 | 32 | 129 |
|  |  | % within Course of study | 30.0% | 37.2% | 31.5% |
| Total | | Count | 323 | 86 | 409 |
|  |  | % within Course of study | 100.0% | 100.0% | 100.0% |

| **Chi-Square Tests** | | | | | | |
| --- | --- | --- | --- | --- | --- | --- |
|  | Value | df | Asymp. Sig. (2-sided) | Exact Sig. (2-sided) | Exact Sig. (1-sided) | Point Probability |
| Pearson Chi-Square | 5.587^a^ | 4 | .232 | .232 |  |  |
| Likelihood Ratio | 5.973 | 4 | .201 | .211 |  |  |
| Fisher's Exact Test | 5.954 |  |  | .198 |  |  |
| Linear-by-Linear Association | .599^b^ | 1 | .439 | .443 | .235 | .029 |
| N of Valid Cases | 409 |  |  |  |  |  |

| a. 1 cells (10.0%) have expected count less than 5. The minimum expected count is 4.42. |
| --- |
| b. The standardized statistic is .774. |

**2. I am very much used to classroom studying and feel online teaching/learning is not very useful. * Place**

| **Crosstab** | | | | | | |
| --- | --- | --- | --- | --- | --- | --- |
|  | | | Place | | | Total |
|  |  |  | 1 | 2 | 3 |  |
| 2. I am very much used to classroom studying and feel online teaching/learning is not very useful. | 1 | Count | 7 | 3 | 11 | 21 |
|  |  | % within Place | 4.4% | 5.0% | 5.8% | 5.1% |
|  | 2 | Count | 29 | 14 | 40 | 83 |
|  |  | % within Place | 18.1% | 23.3% | 21.2% | 20.3% |
|  | 3 | Count | 29 | 15 | 37 | 81 |
|  |  | % within Place | 18.1% | 25.0% | 19.6% | 19.8% |
|  | 4 | Count | 36 | 12 | 47 | 95 |
|  |  | % within Place | 22.5% | 20.0% | 24.9% | 23.2% |
|  | 5 | Count | 59 | 16 | 54 | 129 |
|  |  | % within Place | 36.9% | 26.7% | 28.6% | 31.5% |
| Total | | Count | 160 | 60 | 189 | 409 |
|  |  | % within Place | 100.0% | 100.0% | 100.0% | 100.0% |

| **Chi-Square Tests** | | | | | |
| --- | --- | --- | --- | --- | --- |
|  | Value | df | Asymp. Sig. (2-sided) | Exact Sig. (2-sided) | Exact Sig. (1-sided) |
| Pearson Chi-Square | 5.068^a^ | 8 | .750 | .755 |  |
| Likelihood Ratio | 4.991 | 8 | .759 | .769 |  |
| Fisher's Exact Test | 5.064 |  |  | .754 |  |
| Linear-by-Linear Association | 2.109 | 1 | .146 | .^b^ | .^b^ |
| N of Valid Cases | 409 |  |  |  |  |

| a. 1 cells (6.7%) have expected count less than 5. The minimum expected count is 3.08. |
| --- |
| b. Cannot be computed because there is insufficient memory. |

**2. I am very much used to classroom studying and feel online teaching/learning is not very useful. * E learning skills**

| **Crosstab** | | | | | |
| --- | --- | --- | --- | --- | --- |
|  | | | E learning skills | | Total |
|  |  |  | 1 | 2 |  |
| 2. I am very much used to classroom studying and feel online teaching/learning is not very useful. | 1 | Count | 15 | 6 | 21 |
|  |  | % within E learning skills | 4.6% | 7.2% | 5.1% |
|  | 2 | Count | 72 | 11 | 83 |
|  |  | % within E learning skills | 22.1% | 13.3% | 20.3% |
|  | 3 | Count | 70 | 11 | 81 |
|  |  | % within E learning skills | 21.5% | 13.3% | 19.8% |
|  | 4 | Count | 68 | 27 | 95 |
|  |  | % within E learning skills | 20.9% | 32.5% | 23.2% |
|  | 5 | Count | 101 | 28 | 129 |
|  |  | % within E learning skills | 31.0% | 33.7% | 31.5% |
| Total | | Count | 326 | 83 | 409 |
|  |  | % within E learning skills | 100.0% | 100.0% | 100.0% |

| **Chi-Square Tests** | | | | | | |
| --- | --- | --- | --- | --- | --- | --- |
|  | Value | df | Asymp. Sig. (2-sided) | Exact Sig. (2-sided) | Exact Sig. (1-sided) | Point Probability |
| Pearson Chi-Square | 9.729^a^ | 4 | .045 | .044 |  |  |
| Likelihood Ratio | 9.833 | 4 | .043 | .049 |  |  |
| Fisher's Exact Test | 9.755 |  |  | .042 |  |  |
| Linear-by-Linear Association | 1.786^b^ | 1 | .181 | .189 | .099 | .016 |
| N of Valid Cases | 409 |  |  |  |  |  |

| a. 1 cells (10.0%) have expected count less than 5. The minimum expected count is 4.26. |
| --- |
| b. The standardized statistic is 1.337. |

**3. Unlike in classroom interactive teaching, online teaching will not help develop a student's overall personality and communication * Age**

| **Crosstab** | | | | | |
| --- | --- | --- | --- | --- | --- |
|  | | | Age | | Total |
|  |  |  | 1 | 2 |  |
| 3. Unlike in classroom interactive teaching, online teaching will not help develop a student's overall personality and communication | 1 | Count | 10 | 5 | 15 |
|  |  | % within Age | 3.3% | 4.6% | 3.7% |
|  | 2 | Count | 40 | 14 | 54 |
|  |  | % within Age | 13.3% | 12.8% | 13.2% |
|  | 3 | Count | 43 | 17 | 60 |
|  |  | % within Age | 14.3% | 15.6% | 14.7% |
|  | 4 | Count | 95 | 36 | 131 |
|  |  | % within Age | 31.7% | 33.0% | 32.0% |
|  | 5 | Count | 112 | 37 | 149 |
|  |  | % within Age | 37.3% | 33.9% | 36.4% |
| Total | | Count | 300 | 109 | 409 |
|  |  | % within Age | 100.0% | 100.0% | 100.0% |

| **Chi-Square Tests** | | | | | | |
| --- | --- | --- | --- | --- | --- | --- |
|  | Value | df | Asymp. Sig. (2-sided) | Exact Sig. (2-sided) | Exact Sig. (1-sided) | Point Probability |
| Pearson Chi-Square | .742^a^ | 4 | .946 | .947 |  |  |
| Likelihood Ratio | .729 | 4 | .948 | .949 |  |  |
| Fisher's Exact Test | .922 |  |  | .928 |  |  |
| Linear-by-Linear Association | .328^b^ | 1 | .567 | .597 | .299 | .032 |
| N of Valid Cases | 409 |  |  |  |  |  |

| a. 1 cells (10.0%) have expected count less than 5. The minimum expected count is 4.00. |
| --- |
| b. The standardized statistic is -.573. |

**3. Unlike in classroom interactive teaching, online teaching will not help develop a student's overall personality and communication * Course of study**

| **Crosstab** | | | | | |
| --- | --- | --- | --- | --- | --- |
|  | | | Course of study | | Total |
|  |  |  | 1 | 2 |  |
| 3. Unlike in classroom interactive teaching, online teaching will not help develop a student's overall personality and communication | 1 | Count | 11 | 4 | 15 |
|  |  | % within Course of study | 3.4% | 4.7% | 3.7% |
|  | 2 | Count | 42 | 12 | 54 |
|  |  | % within Course of study | 13.0% | 14.0% | 13.2% |
|  | 3 | Count | 48 | 12 | 60 |
|  |  | % within Course of study | 14.9% | 14.0% | 14.7% |
|  | 4 | Count | 104 | 27 | 131 |
|  |  | % within Course of study | 32.2% | 31.4% | 32.0% |
|  | 5 | Count | 118 | 31 | 149 |
|  |  | % within Course of study | 36.5% | 36.0% | 36.4% |
| Total | | Count | 323 | 86 | 409 |
|  |  | % within Course of study | 100.0% | 100.0% | 100.0% |

| **Chi-Square Tests** | | | | | | |
| --- | --- | --- | --- | --- | --- | --- |
|  | Value | df | Asymp. Sig. (2-sided) | Exact Sig. (2-sided) | Exact Sig. (1-sided) | Point Probability |
| Pearson Chi-Square | .390^a^ | 4 | .983 | .985 |  |  |
| Likelihood Ratio | .373 | 4 | .985 | .986 |  |  |
| Fisher's Exact Test | .602 |  |  | .971 |  |  |
| Linear-by-Linear Association | .137^b^ | 1 | .711 | .715 | .372 | .038 |
| N of Valid Cases | 409 |  |  |  |  |  |

| a. 1 cells (10.0%) have expected count less than 5. The minimum expected count is 3.15. |
| --- |
| b. The standardized statistic is -.370. |

**3. Unlike in classroom interactive teaching, online teaching will not help develop a student's overall personality and communication * Place**

| **Crosstab** | | | | | | |
| --- | --- | --- | --- | --- | --- | --- |
|  | | | Place | | | Total |
|  |  |  | 1 | 2 | 3 |  |
| 3. Unlike in classroom interactive teaching, online teaching will not help develop a student's overall personality and communication | 1 | Count | 4 | 3 | 8 | 15 |
|  |  | % within Place | 2.5% | 5.0% | 4.2% | 3.7% |
|  | 2 | Count | 21 | 6 | 27 | 54 |
|  |  | % within Place | 13.1% | 10.0% | 14.3% | 13.2% |
|  | 3 | Count | 27 | 10 | 23 | 60 |
|  |  | % within Place | 16.9% | 16.7% | 12.2% | 14.7% |
|  | 4 | Count | 52 | 19 | 60 | 131 |
|  |  | % within Place | 32.5% | 31.7% | 31.7% | 32.0% |
|  | 5 | Count | 56 | 22 | 71 | 149 |
|  |  | % within Place | 35.0% | 36.7% | 37.6% | 36.4% |
| Total | | Count | 160 | 60 | 189 | 409 |
|  |  | % within Place | 100.0% | 100.0% | 100.0% | 100.0% |

| **Chi-Square Tests** | | | | | | |
| --- | --- | --- | --- | --- | --- | --- |
|  | Value | df | Asymp. Sig. (2-sided) | Exact Sig. (2-sided) | Exact Sig. (1-sided) | Point Probability |
| Pearson Chi-Square | 3.360^a^ | 8 | .910 | .914 |  |  |
| Likelihood Ratio | 3.455 | 8 | .903 | .909 |  |  |
| Fisher's Exact Test | 3.586 |  |  | .898 |  |  |
| Linear-by-Linear Association | .000^b^ | 1 | .983 | 1.000 | .501 | .018 |
| N of Valid Cases | 409 |  |  |  |  |  |

| a. 1 cells (6.7%) have expected count less than 5. The minimum expected count is 2.20. |
| --- |
| b. The standardized statistic is -.021. |

**3. Unlike in classroom interactive teaching, online teaching will not help develop a student's overall personality and communication * E learning skills**

| **Crosstab** | | | | | |
| --- | --- | --- | --- | --- | --- |
|  | | | E learning skills | | Total |
|  |  |  | 1 | 2 |  |
| 3. Unlike in classroom interactive teaching, online teaching will not help develop a student's overall personality and communication | 1 | Count | 11 | 4 | 15 |
|  |  | % within E learning skills | 3.4% | 4.8% | 3.7% |
|  | 2 | Count | 48 | 6 | 54 |
|  |  | % within E learning skills | 14.7% | 7.2% | 13.2% |
|  | 3 | Count | 45 | 15 | 60 |
|  |  | % within E learning skills | 13.8% | 18.1% | 14.7% |
|  | 4 | Count | 102 | 29 | 131 |
|  |  | % within E learning skills | 31.3% | 34.9% | 32.0% |
|  | 5 | Count | 120 | 29 | 149 |
|  |  | % within E learning skills | 36.8% | 34.9% | 36.4% |
| Total | | Count | 326 | 83 | 409 |
|  |  | % within E learning skills | 100.0% | 100.0% | 100.0% |

| **Chi-Square Tests** | | | | | | |
| --- | --- | --- | --- | --- | --- | --- |
|  | Value | df | Asymp. Sig. (2-sided) | Exact Sig. (2-sided) | Exact Sig. (1-sided) | Point Probability |
| Pearson Chi-Square | 4.352^a^ | 4 | .360 | .362 |  |  |
| Likelihood Ratio | 4.704 | 4 | .319 | .331 |  |  |
| Fisher's Exact Test | 4.676 |  |  | .318 |  |  |
| Linear-by-Linear Association | .100^b^ | 1 | .752 | .792 | .400 | .040 |
| N of Valid Cases | 409 |  |  |  |  |  |

| a. 1 cells (10.0%) have expected count less than 5. The minimum expected count is 3.04. |
| --- |
| b. The standardized statistic is .316. |

**4. Online learning is very similar to watching YouTube lectures. The only difference in Zoom, your teacher is taking class * Age**

| **Crosstab** | | | | | |
| --- | --- | --- | --- | --- | --- |
|  | | | Age | | Total |
|  |  |  | 1 | 2 |  |
| 4. Online learning is very similar to watching YouTube lectures. The only difference in Zoom, your teacher is taking class | 1 | Count | 10 | 3 | 13 |
|  |  | % within Age | 3.3% | 2.8% | 3.2% |
|  | 2 | Count | 44 | 16 | 60 |
|  |  | % within Age | 14.7% | 14.7% | 14.7% |
|  | 3 | Count | 52 | 14 | 66 |
|  |  | % within Age | 17.3% | 12.8% | 16.1% |
|  | 4 | Count | 61 | 29 | 90 |
|  |  | % within Age | 20.3% | 26.6% | 22.0% |
|  | 5 | Count | 133 | 47 | 180 |
|  |  | % within Age | 44.3% | 43.1% | 44.0% |
| Total | | Count | 300 | 109 | 409 |
|  |  | % within Age | 100.0% | 100.0% | 100.0% |

| **Chi-Square Tests** | | | | | | |
| --- | --- | --- | --- | --- | --- | --- |
|  | Value | df | Asymp. Sig. (2-sided) | Exact Sig. (2-sided) | Exact Sig. (1-sided) | Point Probability |
| Pearson Chi-Square | 2.540^a^ | 4 | .638 | .646 |  |  |
| Likelihood Ratio | 2.535 | 4 | .638 | .648 |  |  |
| Fisher's Exact Test | 2.472 |  |  | .656 |  |  |
| Linear-by-Linear Association | .136^b^ | 1 | .712 | .747 | .376 | .035 |
| N of Valid Cases | 409 |  |  |  |  |  |

| a. 1 cells (10.0%) have expected count less than 5. The minimum expected count is 3.46. |
| --- |
| b. The standardized statistic is .369. |

**4. Online learning is very similar to watching YouTube lectures. The only difference in Zoom, your teacher is taking class * Course of study**

| **Crosstab** | | | | | |
| --- | --- | --- | --- | --- | --- |
|  | | | Course of study | | Total |
|  |  |  | 1 | 2 |  |
| 4. Online learning is very similar to watching YouTube lectures. The only difference in Zoom, your teacher is taking class | 1 | Count | 9 | 4 | 13 |
|  |  | % within Course of study | 2.8% | 4.7% | 3.2% |
|  | 2 | Count | 52 | 8 | 60 |
|  |  | % within Course of study | 16.1% | 9.3% | 14.7% |
|  | 3 | Count | 56 | 10 | 66 |
|  |  | % within Course of study | 17.3% | 11.6% | 16.1% |
|  | 4 | Count | 68 | 22 | 90 |
|  |  | % within Course of study | 21.1% | 25.6% | 22.0% |
|  | 5 | Count | 138 | 42 | 180 |
|  |  | % within Course of study | 42.7% | 48.8% | 44.0% |
| Total | | Count | 323 | 86 | 409 |
|  |  | % within Course of study | 100.0% | 100.0% | 100.0% |

| **Chi-Square Tests** | | | | | | |
| --- | --- | --- | --- | --- | --- | --- |
|  | Value | df | Asymp. Sig. (2-sided) | Exact Sig. (2-sided) | Exact Sig. (1-sided) | Point Probability |
| Pearson Chi-Square | 5.463^a^ | 4 | .243 | .244 |  |  |
| Likelihood Ratio | 5.710 | 4 | .222 | .241 |  |  |
| Fisher's Exact Test | 5.618 |  |  | .224 |  |  |
| Linear-by-Linear Association | 1.820^b^ | 1 | .177 | .193 | .096 | .016 |
| N of Valid Cases | 409 |  |  |  |  |  |

| a. 1 cells (10.0%) have expected count less than 5. The minimum expected count is 2.73. |
| --- |
| b. The standardized statistic is 1.349. |

**4. Online learning is very similar to watching YouTube lectures. The only difference in Zoom, your teacher is taking class * Place**

| **Crosstab** | | | | | | |
| --- | --- | --- | --- | --- | --- | --- |
|  | | | Place | | | Total |
|  |  |  | 1 | 2 | 3 |  |
| 4. Online learning is very similar to watching YouTube lectures. The only difference in Zoom, your teacher is taking class | 1 | Count | 5 | 1 | 7 | 13 |
|  |  | % within Place | 3.1% | 1.7% | 3.7% | 3.2% |
|  | 2 | Count | 22 | 4 | 34 | 60 |
|  |  | % within Place | 13.8% | 6.7% | 18.0% | 14.7% |
|  | 3 | Count | 34 | 8 | 24 | 66 |
|  |  | % within Place | 21.2% | 13.3% | 12.7% | 16.1% |
|  | 4 | Count | 29 | 19 | 42 | 90 |
|  |  | % within Place | 18.1% | 31.7% | 22.2% | 22.0% |
|  | 5 | Count | 70 | 28 | 82 | 180 |
|  |  | % within Place | 43.8% | 46.7% | 43.4% | 44.0% |
| Total | | Count | 160 | 60 | 189 | 409 |
|  |  | % within Place | 100.0% | 100.0% | 100.0% | 100.0% |

| **Chi-Square Tests** | | | | | | |
| --- | --- | --- | --- | --- | --- | --- |
|  | Value | df | Asymp. Sig. (2-sided) | Exact Sig. (2-sided) | Exact Sig. (1-sided) | Point Probability |
| Pearson Chi-Square | 12.757^a^ | 8 | .120 | .^b^ |  |  |
| Likelihood Ratio | 13.074 | 8 | .109 | .^b^ |  |  |
| Fisher's Exact Test | .^b^ |  |  | .^b^ |  |  |
| Linear-by-Linear Association | .045^c^ | 1 | .831 | .842 | .425 | .017 |
| N of Valid Cases | 409 |  |  |  |  |  |

| a. 1 cells (6.7%) have expected count less than 5. The minimum expected count is 1.91. |
| --- |
| b. Cannot be computed because there is insufficient memory. |
| c. The standardized statistic is -.213. |

**4. Online learning is very similar to watching YouTube lectures. The only difference in Zoom, your teacher is taking class * E learning skills**

| **Crosstab** | | | | | |
| --- | --- | --- | --- | --- | --- |
|  | | | E learning skills | | Total |
|  |  |  | 1 | 2 |  |
| 4. Online learning is very similar to watching YouTube lectures. The only difference in Zoom, your teacher is taking class | 1 | Count | 10 | 3 | 13 |
|  |  | % within E learning skills | 3.1% | 3.6% | 3.2% |
|  | 2 | Count | 55 | 5 | 60 |
|  |  | % within E learning skills | 16.9% | 6.0% | 14.7% |
|  | 3 | Count | 56 | 10 | 66 |
|  |  | % within E learning skills | 17.2% | 12.0% | 16.1% |
|  | 4 | Count | 67 | 23 | 90 |
|  |  | % within E learning skills | 20.6% | 27.7% | 22.0% |
|  | 5 | Count | 138 | 42 | 180 |
|  |  | % within E learning skills | 42.3% | 50.6% | 44.0% |
| Total | | Count | 326 | 83 | 409 |
|  |  | % within E learning skills | 100.0% | 100.0% | 100.0% |

| **Chi-Square Tests** | | | | | | |
| --- | --- | --- | --- | --- | --- | --- |
|  | Value | df | Asymp. Sig. (2-sided) | Exact Sig. (2-sided) | Exact Sig. (1-sided) | Point Probability |
| Pearson Chi-Square | 9.016^a^ | 4 | .061 | .059 |  |  |
| Likelihood Ratio | 10.143 | 4 | .038 | .044 |  |  |
| Fisher's Exact Test | 9.639 |  |  | .042 |  |  |
| Linear-by-Linear Association | 5.051^b^ | 1 | .025 | .025 | .013 | .003 |
| N of Valid Cases | 409 |  |  |  |  |  |

| a. 1 cells (10.0%) have expected count less than 5. The minimum expected count is 2.64. |
| --- |
| b. The standardized statistic is 2.247. |

**5. Online teaching/learning is extremely useful during calamities like during the coronavirus pandemic * Age**

| **Crosstab** | | | | | |
| --- | --- | --- | --- | --- | --- |
|  | | | Age | | Total |
|  |  |  | 1 | 2 |  |
| 5. Online teaching/learning is extremely useful during calamities like during the coronavirus pandemic | 1 | Count | 12 | 4 | 16 |
|  |  | % within Age | 4.0% | 3.7% | 3.9% |
|  | 2 | Count | 11 | 3 | 14 |
|  |  | % within Age | 3.7% | 2.8% | 3.4% |
|  | 3 | Count | 31 | 19 | 50 |
|  |  | % within Age | 10.3% | 17.4% | 12.2% |
|  | 4 | Count | 101 | 40 | 141 |
|  |  | % within Age | 33.7% | 36.7% | 34.5% |
|  | 5 | Count | 145 | 43 | 188 |
|  |  | % within Age | 48.3% | 39.4% | 46.0% |
| Total | | Count | 300 | 109 | 409 |
|  |  | % within Age | 100.0% | 100.0% | 100.0% |

| **Chi-Square Tests** | | | | | | |
| --- | --- | --- | --- | --- | --- | --- |
|  | Value | df | Asymp. Sig. (2-sided) | Exact Sig. (2-sided) | Exact Sig. (1-sided) | Point Probability |
| Pearson Chi-Square | 5.098^a^ | 4 | .277 | .279 |  |  |
| Likelihood Ratio | 4.915 | 4 | .296 | .313 |  |  |
| Fisher's Exact Test | 4.968 |  |  | .286 |  |  |
| Linear-by-Linear Association | 1.318^b^ | 1 | .251 | .253 | .138 | .022 |
| N of Valid Cases | 409 |  |  |  |  |  |

| a. 2 cells (20.0%) have expected count less than 5. The minimum expected count is 3.73. |
| --- |
| b. The standardized statistic is -1.148. |

**5. Online teaching/learning is extremely useful during calamities like during the coronavirus pandemic * Course of study**

| **Crosstab** | | | | | |
| --- | --- | --- | --- | --- | --- |
|  | | | Course of study | | Total |
|  |  |  | 1 | 2 |  |
| 5. Online teaching/learning is extremely useful during calamities like during the coronavirus pandemic | 1 | Count | 10 | 6 | 16 |
|  |  | % within Course of study | 3.1% | 7.0% | 3.9% |
|  | 2 | Count | 11 | 3 | 14 |
|  |  | % within Course of study | 3.4% | 3.5% | 3.4% |
|  | 3 | Count | 34 | 16 | 50 |
|  |  | % within Course of study | 10.5% | 18.6% | 12.2% |
|  | 4 | Count | 109 | 32 | 141 |
|  |  | % within Course of study | 33.7% | 37.2% | 34.5% |
|  | 5 | Count | 159 | 29 | 188 |
|  |  | % within Course of study | 49.2% | 33.7% | 46.0% |
| Total | | Count | 323 | 86 | 409 |
|  |  | % within Course of study | 100.0% | 100.0% | 100.0% |

| **Chi-Square Tests** | | | | | | |
| --- | --- | --- | --- | --- | --- | --- |
|  | Value | df | Asymp. Sig. (2-sided) | Exact Sig. (2-sided) | Exact Sig. (1-sided) | Point Probability |
| Pearson Chi-Square | 10.030^a^ | 4 | .040 | .039 |  |  |
| Likelihood Ratio | 9.586 | 4 | .048 | .062 |  |  |
| Fisher's Exact Test | 10.142 |  |  | .032 |  |  |
| Linear-by-Linear Association | 8.098^b^ | 1 | .004 | .005 | .003 | .001 |
| N of Valid Cases | 409 |  |  |  |  |  |

| a. 2 cells (20.0%) have expected count less than 5. The minimum expected count is 2.94. |
| --- |
| b. The standardized statistic is -2.846. |

**5. Online teaching/learning is extremely useful during calamities like during the coronavirus pandemic * Place**

| **Crosstab** | | | | | | |
| --- | --- | --- | --- | --- | --- | --- |
|  | | | Place | | | Total |
|  |  |  | 1 | 2 | 3 |  |
| 5. Online teaching/learning is extremely useful during calamities like during the coronavirus pandemic | 1 | Count | 6 | 2 | 8 | 16 |
|  |  | % within Place | 3.8% | 3.3% | 4.2% | 3.9% |
|  | 2 | Count | 7 | 3 | 4 | 14 |
|  |  | % within Place | 4.4% | 5.0% | 2.1% | 3.4% |
|  | 3 | Count | 24 | 7 | 19 | 50 |
|  |  | % within Place | 15.0% | 11.7% | 10.1% | 12.2% |
|  | 4 | Count | 43 | 22 | 76 | 141 |
|  |  | % within Place | 26.9% | 36.7% | 40.2% | 34.5% |
|  | 5 | Count | 80 | 26 | 82 | 188 |
|  |  | % within Place | 50.0% | 43.3% | 43.4% | 46.0% |
| Total | | Count | 160 | 60 | 189 | 409 |
|  |  | % within Place | 100.0% | 100.0% | 100.0% | 100.0% |

| **Chi-Square Tests** | | | | | | |
| --- | --- | --- | --- | --- | --- | --- |
|  | Value | df | Asymp. Sig. (2-sided) | Exact Sig. (2-sided) | Exact Sig. (1-sided) | Point Probability |
| Pearson Chi-Square | 9.166^a^ | 8 | .329 | .328 |  |  |
| Likelihood Ratio | 9.326 | 8 | .316 | .359 |  |  |
| Fisher's Exact Test | 9.526 |  |  | .283 |  |  |
| Linear-by-Linear Association | .019^b^ | 1 | .892 | .896 | .456 | .021 |
| N of Valid Cases | 409 |  |  |  |  |  |

| a. 2 cells (13.3%) have expected count less than 5. The minimum expected count is 2.05. |
| --- |
| b. The standardized statistic is .136. |

**5. Online teaching/learning is extremely useful during calamities like during the coronavirus pandemic * E learning skills**

| **Crosstab** | | | | | |
| --- | --- | --- | --- | --- | --- |
|  | | | E learning skills | | Total |
|  |  |  | 1 | 2 |  |
| 5. Online teaching/learning is extremely useful during calamities like during the coronavirus pandemic | 1 | Count | 11 | 5 | 16 |
|  |  | % within E learning skills | 3.4% | 6.0% | 3.9% |
|  | 2 | Count | 9 | 5 | 14 |
|  |  | % within E learning skills | 2.8% | 6.0% | 3.4% |
|  | 3 | Count | 35 | 15 | 50 |
|  |  | % within E learning skills | 10.7% | 18.1% | 12.2% |
|  | 4 | Count | 118 | 23 | 141 |
|  |  | % within E learning skills | 36.2% | 27.7% | 34.5% |
|  | 5 | Count | 153 | 35 | 188 |
|  |  | % within E learning skills | 46.9% | 42.2% | 46.0% |
| Total | | Count | 326 | 83 | 409 |
|  |  | % within E learning skills | 100.0% | 100.0% | 100.0% |

| **Chi-Square Tests** | | | | | | |
| --- | --- | --- | --- | --- | --- | --- |
|  | Value | df | Asymp. Sig. (2-sided) | Exact Sig. (2-sided) | Exact Sig. (1-sided) | Point Probability |
| Pearson Chi-Square | 7.867^a^ | 4 | .097 | .094 |  |  |
| Likelihood Ratio | 7.274 | 4 | .122 | .151 |  |  |
| Fisher's Exact Test | 8.035 |  |  | .081 |  |  |
| Linear-by-Linear Association | 4.448^b^ | 1 | .035 | .036 | .023 | .005 |
| N of Valid Cases | 409 |  |  |  |  |  |

| a. 2 cells (20.0%) have expected count less than 5. The minimum expected count is 2.84. |
| --- |
| b. The standardized statistic is -2.109. |
